# Design synthesis, characterization, molecular docking and antimicrobial evaluation of novel heterocycles with acrylonitrile and anthracene moieties

**DOI:** 10.1038/s41598-025-03272-5

**Published:** 2025-06-03

**Authors:** Aya. I. Hassaballah, A. K. El-ziaty, Marwa M. Gado, Hayam A. E. Sayed, Mahmoud Kamal, Rania S. Ali

**Affiliations:** 1 Department of Chemistry, Faculty of Science, Ain Shams University, Abbassia, Cairo, 11566 Egypt; 2 Department of Microbiology, Faculty of Science, Ain Shams University, Abbassia, Cairo, 11566 Egypt; 3 Department of Entomology, Faculty of Science, Ain Shams University, Abbassia, Cairo, 11566 Egypt; 4 Department of Basic Science, Faculty of Technology and Education, Helwan University, Cairo, Egypt

**Keywords:** 2-Cyanoacryloyl chloride, Anthracene, Acrylonitrile, Benzo[d]thiazole, Benzo[d]imidazole, Molecular docking, Antimicrobial resistance, PBP2a inhibitors, Microbiology, Applied microbiology, Bacteria, Chemistry publishing, Organic chemistry

## Abstract

The synthon 3-(anthracen-9-yl)-2-cyanoacryloyl chloride **4** was produced and exploited in the creation of a wide variety of highly reactive heterocyclic compounds, by its interaction with diverse nitrogen nucleophiles. Using spectral and elemental analysis, the structures of each synthesized heterocycles were fully investigated. Ten of the thirteen novel heterocycles showed encouraging efficacy against antibiotic-resistant bacteria (MRSA). Among these, compounds **6**, **7**, **10**, **13b**, and **14** demonstrated the highest antibacterial activity, showing inhibition zones near 4 cm. However, molecular docking studies revealed varied binding affinities for Penicillin-Binding Protein 2a (PBP2a), a crucial target in MRSA resistance. Some compounds, such as **7**, **10**, and **14**, displayed higher binding affinities and interaction stability within the PBP2a active site compared to the co-crystallized quinazolinone ligand. In contrast, compounds **6** and **13b** exhibited lower docking scores but still showed substantial antimicrobial activity, with **6** showing the lowest MIC (9.7 μg/100 μL) and MBC (78.125 μg/100 μL) values. The docking analysis revealed key interactions, including hydrogen bonding and π-stacking, particularly with residues like **Lys 273**, **Lys 316**, and **Arg 298**, which were identified as interacting with the co-crystallized ligand within the crystal structure of **PBP2a**. These residues are essential for the enzymatic activity of PBP2a. These findings suggest that the synthesized compounds could serve as promising anti-MRSA agents, highlighting the importance of integrating molecular docking with biological assays to identify effective therapeutic candidates.

## Introduction

In the previous years of this century, research efforts have focused on developing new, simple procedures and techniques for the synthesis of several innovative heterocyclic ring systems with antibacterial applications utilizing easily accessible starting materials.

**Fig. 1:**
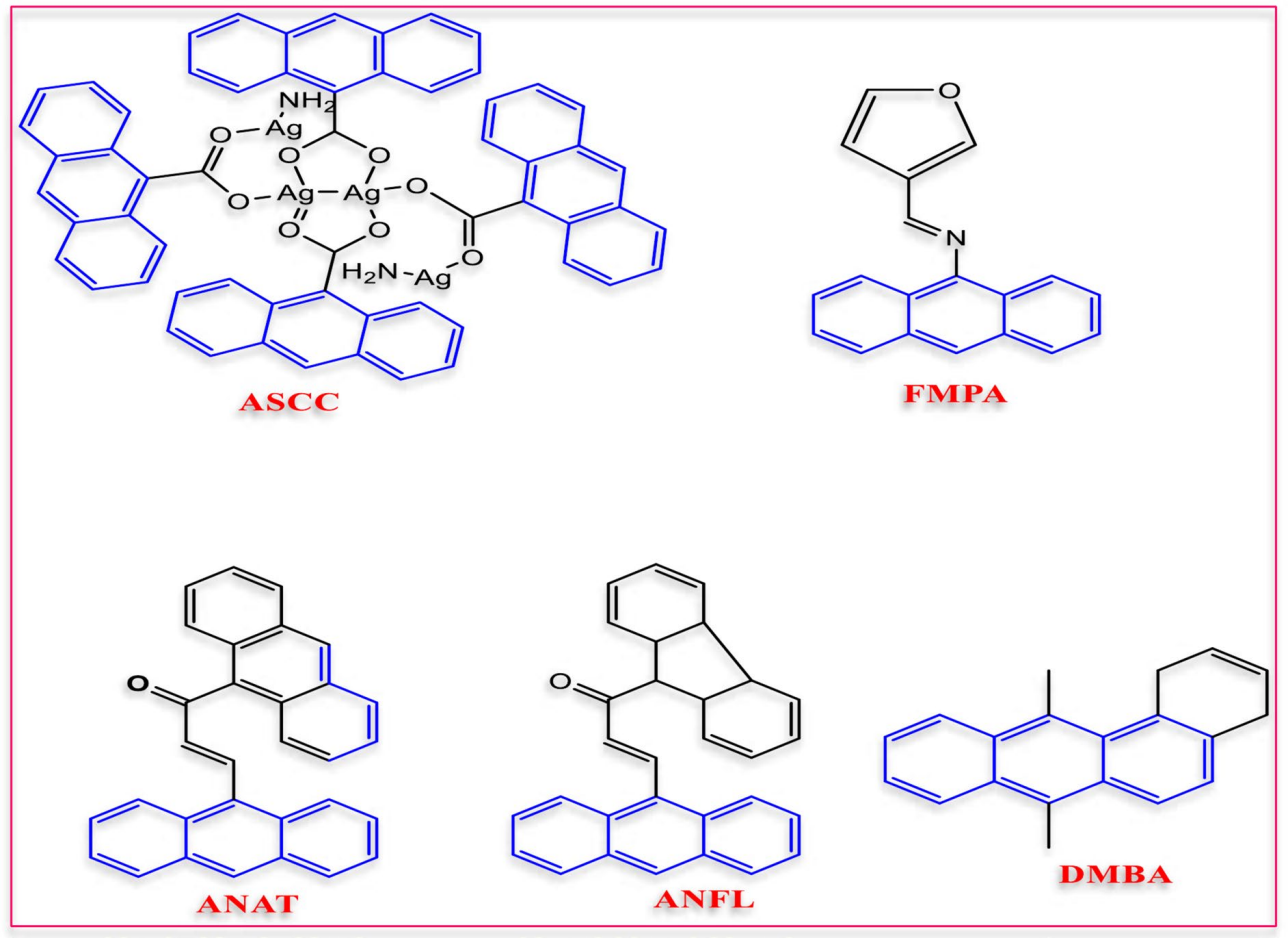
Antimicrobial compounds containing anthracene moiety.

**Fig. 2:**
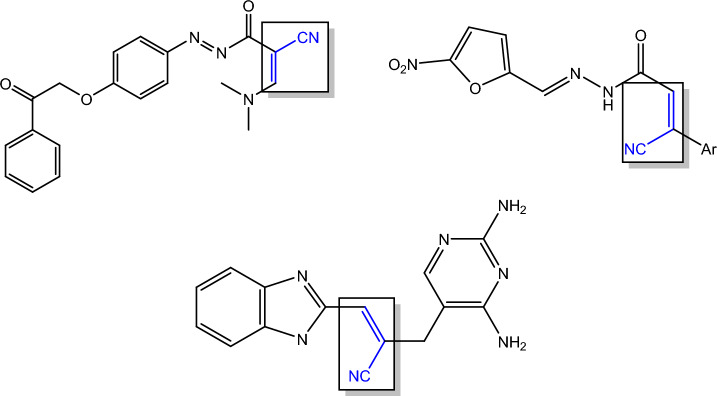
The acrylonitrile moiety as antimicrobial agent.

Acrylonitrile moiety is considered an important starting material in the synthesis of many remarkable heterocyclic ring systems, as they are highly reactive compounds. Furthermore, the recent widespread utilization of 2-cyanoacryloyl chloride derivatives in the design and synthesis of highly crucial products utilized in pharmacological applications, like precursors for pharmaceutical intermediates^1–3^, anti-HIV, antiviral, anticancer, antimicrobial, antidepressant, and antioxidant^4–10^. Recently, the biological efficacy of anthracene and its derivatives, including their, antibiotic, anticancer^11,12^, antibacterial^13–15^ and insecticidal properties^16,17^ has drawn a lot of interest^18–21^. Antimicrobial compounds having acrylonitrile and anthracene moieties are depicted in Figs. 1 and 2.

According to the World Health Organization (WHO) (2021), antimicrobial resistance (AMR) is a global health and development threat^22–25^. The incurability of patients leads to longer hospitalizations which need more expensive medicines, in addition to increased death rates and disability. The absence of effective antimicrobials normally will lead to failure in treating various infections, especially during chemotherapy and major surgery.

Methicillin-resistant *Staphylococcus aureus* (MRSA) as well as *E. coli* are listed on the priority pathogens list according to the WHO report 2024, where both resist many antibiotics, so they are representing challenging infections to treat and control there for there is an urgent need for new effective antimicrobial compounds to come over this issue. Where anthracenes and their derivatives are well known antimicrobial agents that can affect both Gram positive and Gram-negative pathogens^22–25^ his study targeting synthesis a new derivatives which can combat these health threaten pathogens.

(WHO) has reported that there are many bacterial pathogens that confer resistance against many different antibiotics; including the methicillin-resistant bacteria *Staphylococcus aureus* (MRSA*)* which is a common cause of infections both in the community and in health-care facilities. It was reported that patients with (MRSA) infections have a 64% death rate more than patients with drug-sensitive infections. Also, *Escherichia coli* represents a global risk as the last defense line against carbapenem resistant Enterobacteriaceae i.e. *E. coli* was colistin, but lately colistin resistance bacteria have been reported in several countries^22–25^.

So, based on the World Health Organization’s global action plan on antimicrobial resistance^26^ the discovery and synthesis of novel antimicrobials is urgently needed. The remarkable potential of anthracene and acrylonitrile as antibacterial^27^, antifungal^28^, anticancer^29^, and antioxidant^30^ has been highlighted in a number of publications that have been published in the literature. Given this information, we may say that these derivatives make great anti-MRSA candidates.

The previous literature survey motivated us to synthesize new derivatives within these categories. Therefore, the objective of this study was to design novel heterocyclic systems incorporating anthracene and acrylonitrile moieties, evaluate their antibacterial and antimicrobial efficacy, and investigate their potential binding interactions with Penicillin-Binding Protein 2a (PBP2a) through molecular docking. By building on previous work, this research continues the synthesis of heterocyclic systems, biological evaluation, and computational analysis, aiming to identify promising anti-MRSA agents with strong PBP2a inhibitory activity^31–49^.

## Results and discussion

### Chemistry

The focus of our present study is the synthesis and the anti-microbial evaluation of novel heterocyclic compounds bearing anthracene and acrylonitrile moieties. 3-(anthracen-9-yl)-2-cyanoacryloyl chloride **4** was prepared and used as a building block to create new heterocyclic systems.

Structure of compound **4** was determined using spectral data. The ^1^H-NMR spectrum revealed CH= at 9.26 ppm, the IR spectrum showed the existence of a carbonyl group at 1737 cm^−1^ and a cyano group at 2224 cm^−1^ in addition the ^13^CNMR spectrum supported the suggested structure (cf. Experimental section).

**Fig. 3:**
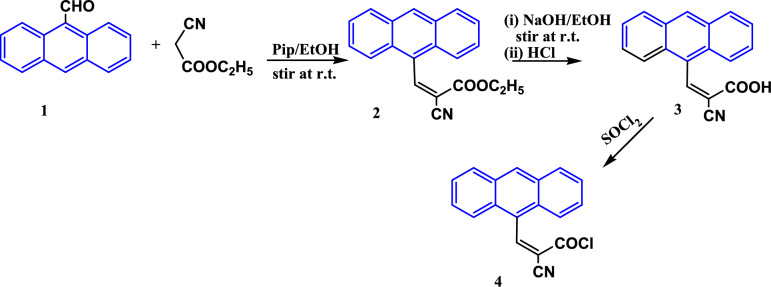
Synthesis of 3-(anthracen-9-yl)-2-cyanoacryloyl chloride **4**.

Synthesis of 3-(anthracen-9-yl)-2-cyanoacryloyl chloride **4** was carried out via the hydrolysis of arylidene **2**^50–53^ by ethanolic sodium hydroxide solution (10%) to give the acid **3**^54–56^ which was treated with thionyl chloride in a water bath to afford the acryloyl chloride derivative **4** in a good yield (88.5%), as shown in Fig. 3.

Aiming to construct novel heterocyclic compounds with anticipated antimicrobial efficiency, the reaction of the acid chloride **4** with different binucleophiles was carried out.

Treatment of acid chloride **4** with hydrazine hydrate at 0° temp for one hour, unfortunately the pyrazalone **5** was not obtained, the product is the acrylamide derivatives, its structure was confirmed from spectral data, its IR spectrum showed absorption bands for C=O at 1720 cm^−1^, C≡N at 2228 cm^−1^, and NH at 3424 cm^−1^ and the ^1^H-NMR spectrum showed olefinic protons and one exchangeable singlet signal for NH proton at 9.3 ppm (cf. Experimental section).

**Fig. 4:**
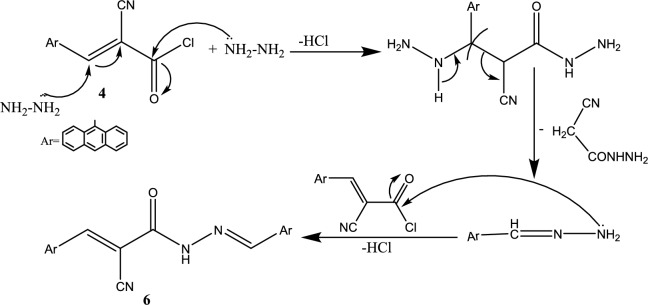
A suggested mechanism for synthesis of *N*′-phenylacrylohydrazide **6**.

The proposed mechanism for the synthesis of the acrylamide derivative **6** was clarified in Fig. 4.

**Fig. 5:**
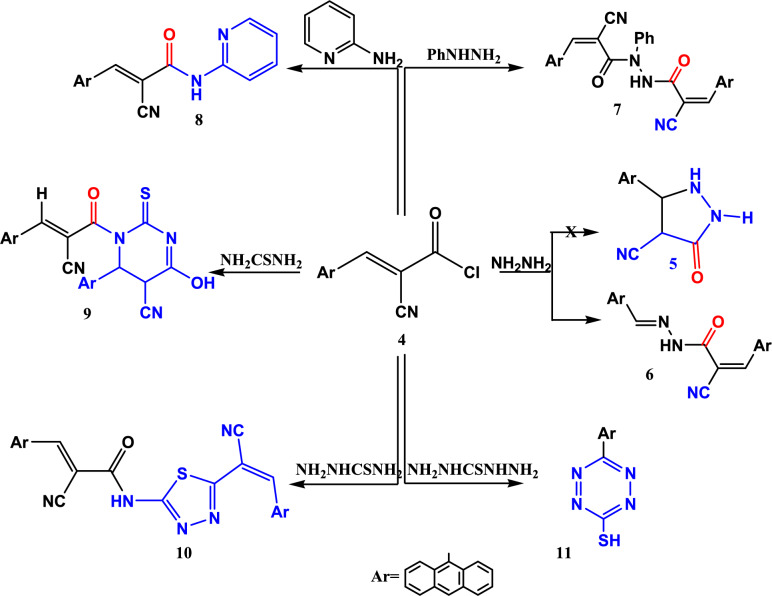
Reactions of acid chloride **4** with different binucleophiles.

*N*-phenylacrylohydrazide derivative **7** was obtained in good yield (77%) by the reaction of two mole of the acid chloride **4** with one mole of phenyl hydrazine (Fig. 5). The structure of **7** was confirmed from its IR spectral date which showed absorption of two C=O groups at 1691 and 1671 cm^−1^, CN group at 2222 cm^−1^ and NH group at 3245 cm^−1^, and its ^1^H-NMR spectrum retained the CH groups at 9.15 and 8.81 ppm and the NH proton at 10.88 ppm. (cf. experimental section).

The reactions of acid chloride 4 with 1,3-binucleophiles were studied in this work. So, treatment of the acid chloride 4 with 2-aminopyridine in 1,4 dioxane and the presence of TEA as a base at room temp afforded the acrylamide derivative 8 (Fig. 5) which structure was elucidated from its spectral date. The IR showed the stretching absorption band of cyano group at 2222 cm^−1^, NH band at 3148 cm^−1^, and carbonyl group at 1665 cm^−1^, and ^1^HNMR clarified the presence of olefinic proton at 9.14 ppm (cf. experimental section).

When compound **4** allowed to react with thiourea, the pyrimidine thione 9 was obtained and when reacted with thiosemicarbazide, we got thiopyrazole derivatives 10 (Fig. 5). The structures of compounds 9 and 10 were confirmed from their spectral and elemental analyses. (cf. Experimental section).

Tetrazine-3-thiol **11** was obtained from the reaction of **4** with thiocarbohydrazide as a 1,4-binucleophile (Fig. 5), and its structure was confirmed by spectral and elemental analysis. In the IR spectrum C=N appeared at 1619 cm^−1^. Meanwhile, its ^1^H-NMR spectrum retained the multiplate signals of aromatic protons at 7.78–8.66 ppm and the SH proton at 3.31 ppm (cf. Experimental section).

The reaction of acryloyl chloride 4 with 1,2 diamino benzene, 2-aminothiophenol, anthranilic acid, 1,2 diamino ethane and ethanol amine as 1,4 binucleophiles to give the novel heterocyclic systems (13–16).

**Fig. 6:**
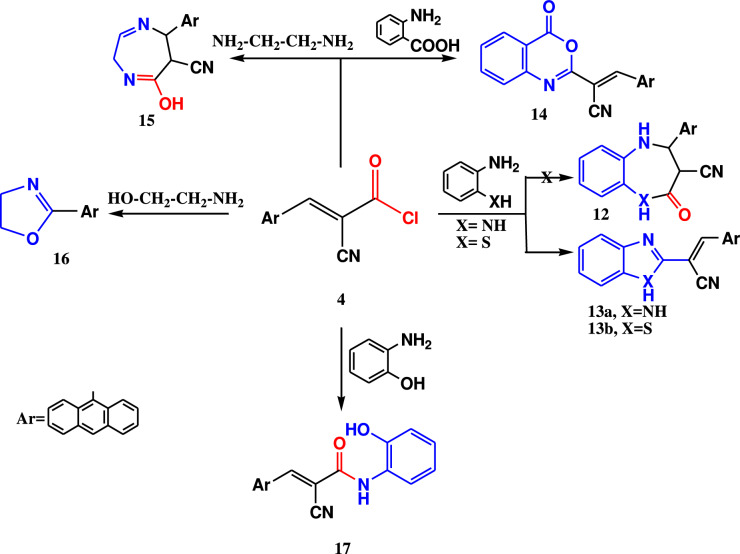
Reactions of 2-cyanoacryloyl chloride **4** with 1,4-binucleophiles.

The structures of these new synthesized compounds were confirmed from their spectral and elemental analysis (cf. Experimental section). While 2-hydroxyphenyl acrylamide derivative **17** was produced when it reacted with 2-amino phenol as a binucleophile (Fig. 6), the structure was confirmed by spectral and elemental analysis. IR spectrum of compound **17** showed C=O and C≡N signals appears at 1681 and 2226 cm^−1^. Meanwhile, its ^1^H-NMR spectrum retained the olefinic proton singlet signal at 9.19 ppm and OH proton appeared at 9.82 ppm (cf. Experimental section).

**Fig. 7:**
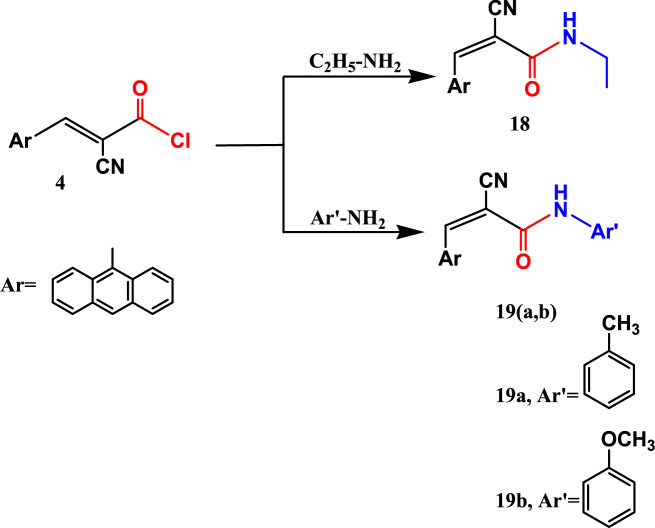
Reactions of acid chloride **4** with mono-nucleophiles.

In case of reactions of acid chloride 4 with mono nucleophiles such as ethyl amine, 4-toluidine, and 4-methoxy aniline in dioxane as a solvent and drops of TEA as a catalyst at room temperature, the reaction prompted the acrylamide derivatives 18, 19a, and 19b as green crystals. The elemental and spectral data of compounds 18, 19a and 19b confirmed the structure of these derivatives, (cf. Experimental section) (Fig. 7).

### Antimicrobial activity

**Fig. 8:**
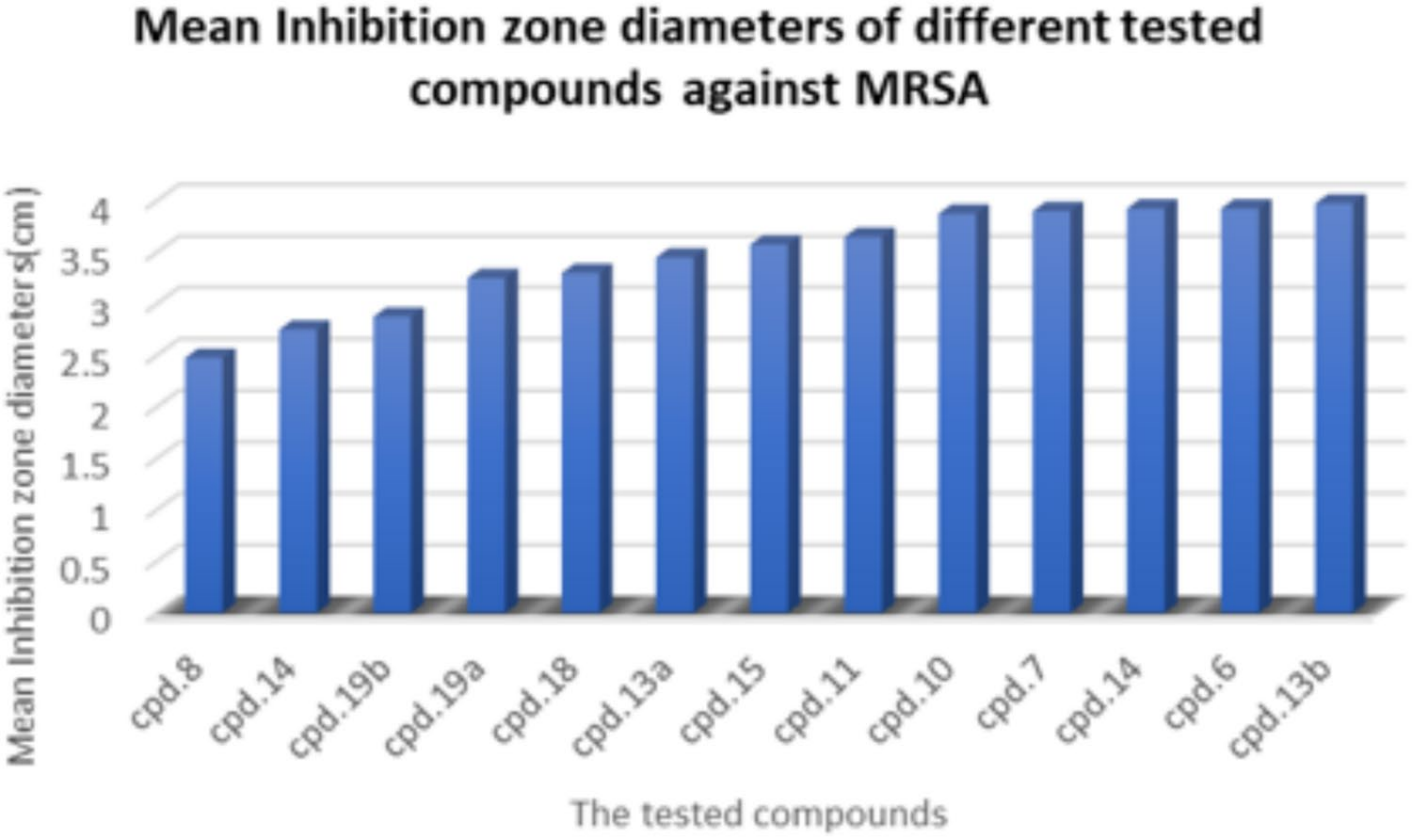
The mean inhibition zone diameters of different tested compounds against MRSA.

**Fig. 9:**
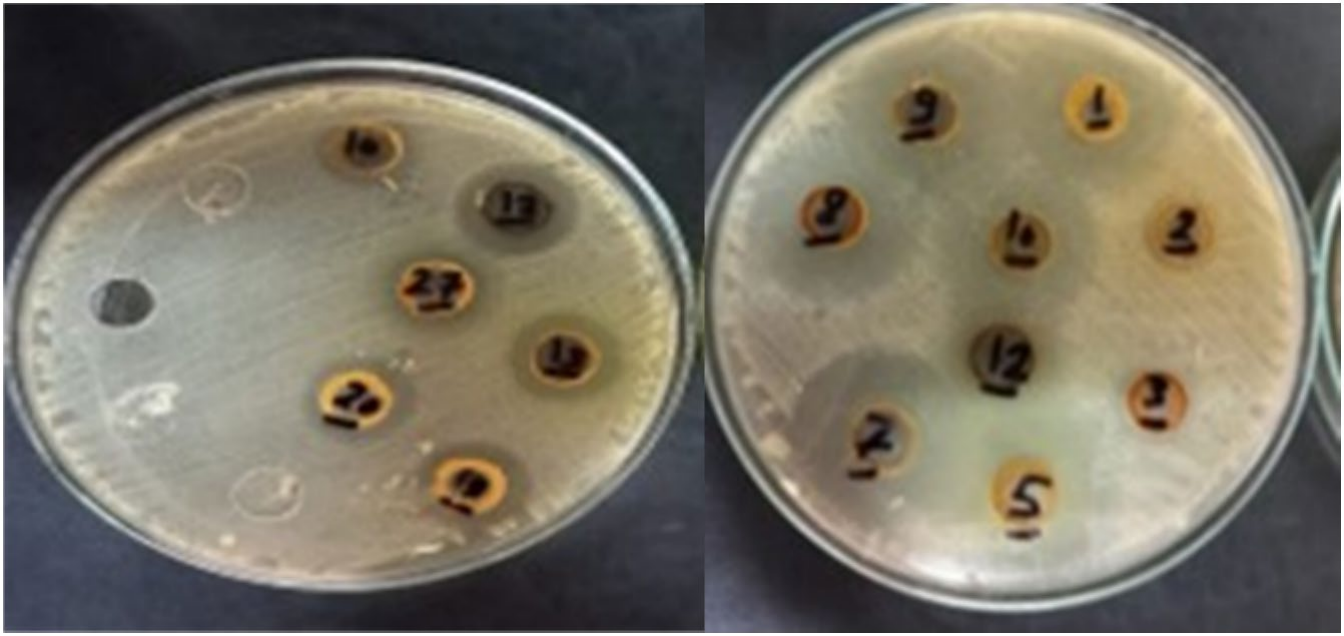
Different inhibition zones formed after treatment with tested compounds.

Upon screening of antimicrobial activity of the different synthesized compounds, different effects are obtained as shown in Table 1 and Fig. 8 (cf. Figures File). All the tested compounds are effective with varying degrees against the gram-positive bacteria MRSA, whereas the gram-negative *E. coli* shows complete resistance against all of them. The tested compounds can be categorized into three distinct groups based on the diameter of their inhibition zones with MRSA. The first group shows the highest activity, and is comprised of 5 compounds (**6**, **7**, **10**, **13b** and **14**). These give inhibition zone diameter near 4 cm; where the highest activity within this group is observed for compound number **6** and **13b**. The second group shows a moderate activity, which represents another five compounds (**11**, **13a**, **15**, **18** and **19a**). These give inhibition zones range between 3.3 and 3.65 cm with the highest activity of 3.65 ± 0.1 cm for compound **11**. On the other hand, the last group, containing three compounds (**8**, **17** and **19b**), shows the lowest antibacterial activity (below 3 cm). Figure 9 represents different appeared inhibition zones.

**Table 1:** Screening of antibacterial activity for the different compounds.

Compound	The mean diameter of inhibition zone (mm) ± std	
	Tested bacteria	
	MRSA	*E. coli*
**6**	4 ± 0.1	0
**7**	3.9 ± 0.1	0
**8**	2.5 ± 0.1	0
**10**	3.9 ± 0.1	0
**11**	3.65 ± 0.1	0
**13a**	3.45 ± 0.1	0
**13b**	4 ± 0.1	0
**14**	3.9 ± 0.1	0
**15**	3.6 ± 0.1	0
**17**	2.75 ± 0.1	0
**18**	3.3 ± 0.2	0
**19a**	3.3 ± 0.2	0
**19b**	2.9 ± 0.1	0

**Fig. 10:**
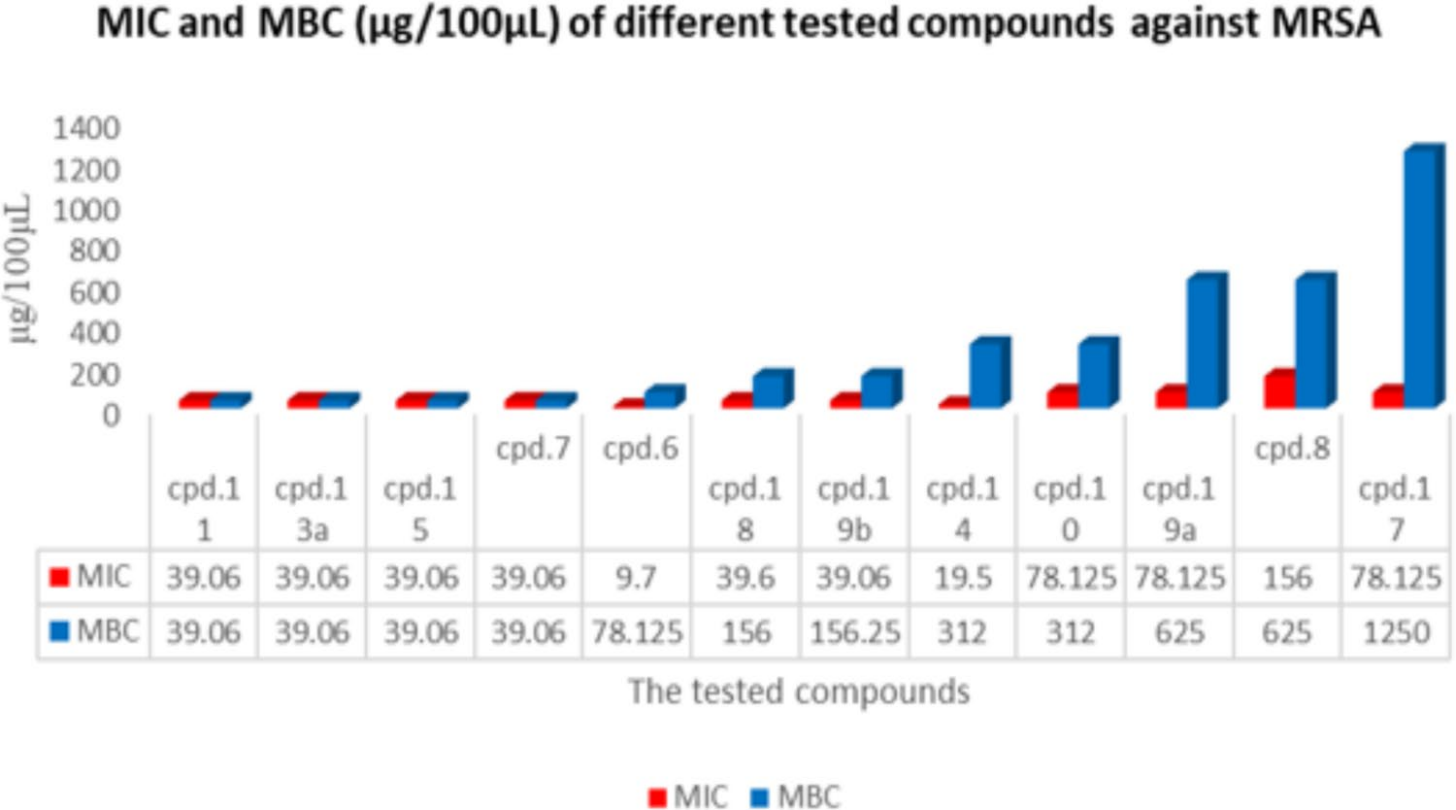
MIC and MBC (µg/100 µL DMSO) for different compounds against MRSA.

Further investigation of the antimicrobial activity of the tested compounds includes MIC and MBC determination for each compound. The results appear slightly different (as shown in Tables 2, 3 and Fig. 10 (cf. Figures File) where compounds **7**, **11**, **13a** and **15** appear to be regrouped together as the best compounds. They show the same lowest MIC and MBC values (39.06 μg/100 μL). Although compounds **7** and **8** show lower MIC values (9.7 μg/100 μL), they reveal higher MBC values (78.125 μg/100 μL). Therefore, they are considered weaker than the priory mentioned compounds. However, these six compounds seem to be the most potent tested compounds as they had MBC values less than 100 μg/100 μL.

**Table 2:** MIC and MBC values for each tested compound against MRSA (μg/100 μL).

Compound	MIC (μg/100 μL)	MBC (μg/100 μL)
**6**	9.70	78.125
**7**	39.08	39.06
**8**	156	625
**10**	78.125	312
**11**	39.06	39.06
**13a**	39.08	39.06
**13b**	9.70	78.125
**14**	19.50	312
**15**	39.06	39.06
**17**	78.125	1250
**18**	39.60	156
**19a**	78.125	625
**19b**	39.06	156.25

**Table 3:** MIC and MBC values for each tested compound against MRSA (μg/100 μL).

Compound	MIC (μg/100 μL)	MBC (μg/100 μL)	MBC/MIC (tolerance level)	The mode of action
**6**	9.7	78.125	> 8	Static
**7**	39.08	39.06	< 1	Cidal
**8**	156	625	4	Cidal
**10**	78.125	312	< 4	Cidal
**11**	39.06	39.06	1	Cidal
**13a**	39.08	39.06	< 1	Cidal
**13b**	9.7	78.125	> 8	Static
**14**	19.5	312	16	Static
**15**	39.06	39.06	1	Cidal
**17**	78.125	1250	16	Static
**18**	39.6	156	< 4	Cidal
**19a**	78.125	625	8	Static
**19b**	39.06	156.25	4	Cidal

Compounds (**10**, **14**, **18** and **19b**) reveal moderated activity in comparison with other tested compounds as they give MBC values, which range between 156 and 312 μg/100 μL. On the other hand, the compounds (**8**, **17** and **19a**) are the least promising as they possess the highest MBC values (625, 625 and 1250 μg/100 μL) respectively.

Finally, according to the tolerance level as shown in Table 3, the tested compound can be categorized according to their mode of action into two categories; compounds that showed a bactericidal effect (**7**, **8**, **10**, **11**, **13a**, **15**, **18**, **19b**); and those exhibited bacteriostatic effects (6, **13b**, **14**, **17**, **19a**). with the priority for compounds **7**, **11**, **13a** and **15** where they showed their killing activity at very low concentration (39.06 μg/100 μL).

## Future work

Ten out of the thirteen tested compounds show promise in fighting antibiotic resistance MRSA. As a result, further screening is recommended using more antibiotic resistance pathogens especially local isolates that cover pathogenic gram-positive and gram-negative bacteria, as well as the pathogenic yeast fungi and the cytotoxicity testing for each compound for safety evaluation.

### Molecular docking of synthesized compounds to PBP2a

Molecular docking studies were performed to assess the potential of the synthesized compounds as inhibitors of Penicillin-Binding Protein 2a (PBP2a) in Methicillin-Resistant *Staphylococcus aureus* (MRSA). Inhibiting PBP2a, a critical enzyme involved in bacterial cell wall biosynthesis, interferes with cell wall formation, ultimately leading to bacterial lysis and cell death^1^. The docking results, presented in Table 4 and further detailed in Supplementary Data File, demonstrated that several compounds exhibited a strong binding affinity for PBP2a, particularly with key active site residues such as Lys 273, Lys 316, and Arg 298. The interactions, including hydrogen bonding and π-stacking, closely mirrored those seen with the co-crystallized quinazolinone ligand (CCL), indicating the potential of these compounds as effective inhibitors.

**Table 4:** Molecular docking data for the 14 synthesized organic compounds docked against penicillin-binding protein 2a (PBP2a) from methicillin-resistant *Staphylococcus aureus* (MRSA), using the crystal structure of PBP2a (PDB ID: 4CJN).

Code	S	RMSD	Interaction type: interacting residues
**6**	− 5.98	1.40	H-acceptor: LYS 273 (B) and LYS 316 (B), pi-H: ASN 104 (B)
**7**	− 6.32	1.48	H-acceptor: LYS 273 (B), pi-H: ASN 146 (B)
**8**	− 5.92	1.85	H-acceptor: LYS 273 (B) and ASP 295 (B), pi-H: TYR 297 (B), pi-cation: LYS 316 (B)
**9**	− 6.36	1.84	H-acceptor: LYS 273 (B)
**10**	− 6.40	1.68	H-acceptor: LYS 273 (B) and LYS 273 (B), pi-H: TYR 105 (B) and TYR 105 (B)
**13a**	− 5.35	1.37	H-acceptor: LYS 316 (B)
**13b**	− 5.63	0.53	H-acceptor: LYS 316 (B), pi-H: ASN 146 (B)
**14**	− 6.04	1.16	H-acceptor: LYS 273 (B), pi-H: ASP 295 (B)
**15**	− 5.24	1.01	H-acceptor: ARG 298 (A), pi-H: ILE 309 (A)
**16**	− 4.88	1.24	H-acceptor: LYS 273 (B)
**17**	− 5.94	1.20	H-acceptor: LYS 273 (B) and LYS 316 (B), pi-H: GLU 294 (B) and ASP 295 (B) and TYR 297 (B)
**18**	− 5.23	0.96	H-acceptor: ARG 298 (A) and ARG 298 (A), H-donor: GLU 145 (B)
**19a**	− 5.47	1.77	H-acceptor: ARG 298 (A), pi-H: ILE 309 (A)
**19b**	− 5.79	1.94	H-acceptor: LYS 273 (B), H-pi: HIS 293 (B), pi-H: LYS 273 (B), pi-cation: LYS 273 (B) and LYS 273 (B)
**CCL**	− 5.95	1.53	H-acceptor: ARG 298 (A) and LYS 273 (B) and LYS 316 (B) and LYS 273 (B), Ionic: LYS 273 (B) and LYS 316

The table compares the binding scores (S), root mean square deviation (RMSD) values, interaction type and key interacting residues for each compound, alongside the redocked co-crystallized quinazolinone ligand (CCL) data.

The molecular docking data, along with other computational parameters, strongly indicate that PBP2a inhibition is a key mechanism contributing to the observed antimicrobial activity of these compounds. The docking scores and root mean square deviation (RMSD) values provided further insights into binding affinity and stability, supporting this hypothesis. As detailed in Table 4, while several compounds showed favorable binding affinities, some such as **7**, **9**, **10**, and **14** had higher docking scores than the co-crystallized ligand, suggesting potentially stronger interactions with PBP2a active site residues. However, the most biologically active compounds, 6 and 13b, displayed slightly lower docking scores (− 5.98 and − 5.63, respectively), compared to other ligands. This suggests that while docking scores are useful for predicting binding affinity, other factors such as ligand stability and molecular interactions in a biological environment also play crucial roles in determining antimicrobial potency. Notably, all synthesized compounds showed RMSD values below 2 Å, confirming that their docking poses were structurally consistent with the co-crystallized ligand’s binding conformation, further supporting their potential as effective PBP2a inhibitors.

**Fig. 11:**
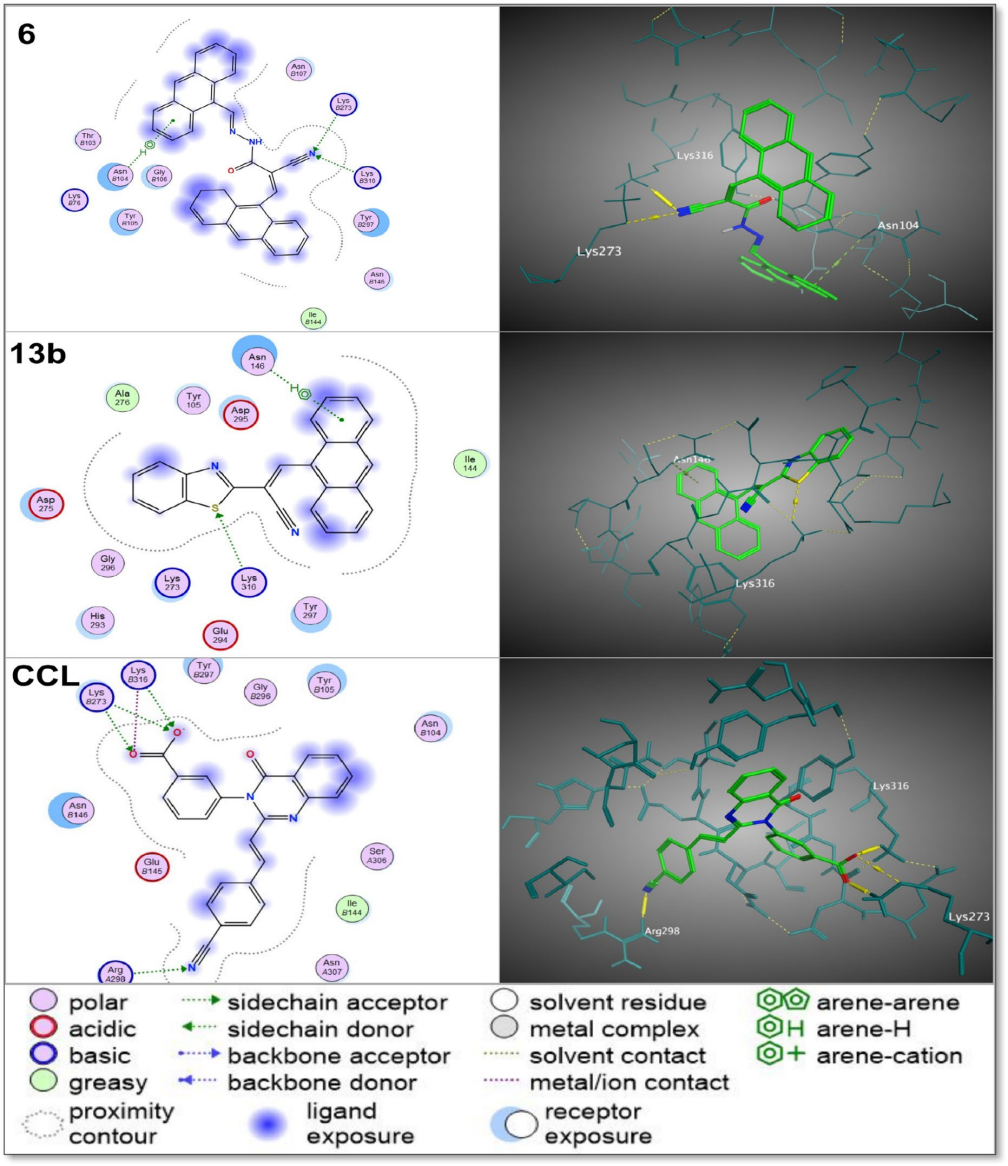
2D and 3D interaction diagrams of the highest active compounds, 6 and 13b, docked against Penicillin-Binding Protein 2a (PBP2a) from Methicillin-Resistant *Staphylococcus aureus* (MRSA), using the crystal structure of PBP2a (PDB ID: 4CJN). The diagrams compare the interaction modes of these compounds with those of the redocked co-crystallized quinazolinone ligand (CCL), highlighting key interactions such as hydrogen bonds, π-stacking, and ionic interactions.

Despite the valuable predictions provided by docking scores and RMSD values, the correlation between these docking results and antimicrobial activity was not always straightforward. While PBP2a inhibition is strongly supported as a key factor contributing to antimicrobial activity, certain discrepancies suggest that other biological properties also play a significant role. Compounds **6** and **13b** exhibited the highest antimicrobial activity, with inhibition zone diameters of 4 cm each and the lowest MIC (9.7 μg/100 μL) and MBC (78.125 μg/100 μL) values, despite having lower docking scores compared to compounds **7**, **9**, **10**, and **14**. This indicates that while PBP2a inhibition contributes to antimicrobial potency, factors such as solubility, bioavailability, and interaction dynamics within the bacterial environment also influence overall effectiveness. Figure 11 illustrates their docking poses, showing that both compounds still interact with critical PBP2a residues, potentially stabilizing the inhibitory complex, even with their relatively lower binding scores. This highlights that while molecular docking provides important insights into PBP2a inhibition, additional biological factors must be considered to fully understand the real-world antimicrobial effectiveness of these compounds.

A similar pattern was observed with compound **7**, which showed a relatively strong docking score (− 6.32) and an inhibition zone diameter (3.9 cm) similar to compound **10**. However, its MIC (39.08 μg/100 μL) and MBC (39.06 μg/100 μL) were notably higher, indicating that it required a much greater concentration to exert antimicrobial effects. This suggests that while compound **7** demonstrated strong binding affinity in docking studies, factors such as bioavailability, cellular uptake, or other physicochemical properties may have limited its biological efficacy. Although compound **7** exhibited bactericidal properties, it was less effective at inhibiting bacterial growth compared to compounds **6** and **13b**.

A more significant discrepancy was observed with compound **10**, which exhibited the highest docking score (− 6.40), suggesting a strong binding affinity for PBP2a. However, its inhibition zone diameter (3.9 cm) was comparable to compound **7**, and its MBC (312 μg/100 μL) was considerably higher than those of compounds **6**, **7**, and **13b**, indicating weaker bactericidal activity. This suggests that, despite favorable docking predictions, compound 10 was less effective at killing MRSA due to other limiting factors, such as solubility, stability, or poor permeability across bacterial membranes. These results reinforce the understanding that while PBP2a inhibition plays a key role in antimicrobial activity, it does not fully explain the variations in biological activity observed among the tested compounds. The discrepancies suggest that further experimental assays and in-depth biological evaluations are necessary to fully elucidate the antimicrobial mechanisms at work.

The molecular docking results in Table 4 and Supplementary Data File highlight the complex relationship between docking scores and antimicrobial activity. While compounds **6** and **13b** had lower docking scores compared to compounds **7**, **9**, **10**, and **14**, they exhibited the highest antimicrobial potency. Their interaction profiles, shown in Fig. 11, demonstrate that despite weaker binding scores, they still form essential hydrogen bonding and π-stacking interactions with critical PBP2a residues, likely stabilizing the enzyme-inhibitor complex in a biologically favorable manner. The enhanced antimicrobial effectiveness of **6** and **13b**, despite their relatively weaker docking scores, suggests that other properties such as solubility, stability, and cellular uptake should be considered alongside docking data when evaluating inhibitor potential. This highlights the importance of integrating docking studies with experimental antimicrobial assays to accurately assess the therapeutic potential of novel compounds.

These findings underscore that while molecular docking is a powerful tool for predicting binding affinity and identifying potential inhibitory mechanisms, it should not be relied upon exclusively to determine antimicrobial effectiveness. The molecular data suggest that PBP2a inhibition is a key factor contributing to antimicrobial activity, but the variations in biological activity indicate that other physicochemical and pharmacokinetic properties must be optimized for enhanced therapeutic efficacy. Future research should focus on optimizing the chemical structures of compounds **7** and **10** to improve bioavailability and cellular uptake, ensuring that strong docking interactions translate into real-world antimicrobial potency. Further studies, including additional biological assays and structure–activity relationship (SAR) analyses, will be crucial to refine the understanding of how these compounds function as PBP2a inhibitors and to develop more effective antimicrobial agents.

### Structure activity relationships (SAR)

The synthesized compounds derived from 3-(anthracen-9-yl)-2-cyanoacryloyl chloride **4** exhibited varying levels of antimicrobial activity, with several demonstrating substantial inhibition against methicillin-resistant *Staphylococcus aureus* (MRSA). A structure–activity relationship (SAR) analysis revealed key structural features responsible for the antibacterial efficacy of these compounds.

The presence of both acrylonitrile and anthracene groups proved crucial in enhancing the antimicrobial activity. The highly reactive nitrile group in acrylonitrile is essential for facilitating interactions with bacterial proteins, thereby contributing to the compounds’ antimicrobial properties. Compounds incorporating both acrylonitrile and anthracene consistently displayed stronger antibacterial effects. The aromatic nature of the anthracene group further stabilized these compounds, likely augmenting their biological activity.

The incorporation of heterocyclic rings significantly boosted the potency of several derivatives. Notably, the benzothiazole derivative **13b** and the acrylohydrazide derivative **6** exhibited the highest antibacterial activity, with inhibition zones of approximately 4 cm. These heterocyclic derivatives showed more pronounced biological effects, indicating that the heterocyclic structures play a pivotal role in bacterial inhibition. Similarly, the pyrimidine thione in compound **9**, the thiopyrazole in compound **10**, and the tetrazine ring in compound **11** all contributed to the compounds’ antimicrobial properties, further emphasizing the importance of heterocyclic modifications.

Among the synthesized compounds, **6** and **13b** stood out for their superior antimicrobial potency. Compound **6** demonstrated the lowest minimum inhibitory concentration (MIC) of 9.7 μg/100 μL and the lowest minimum bactericidal concentration (MBC) of 78.125 μg/100 μL, highlighting its remarkable ability to eradicate MRSA. Likewise, compound 13b showed an inhibition zone of 4 cm, along with low MIC and MBC values, confirming its potent antimicrobial effects. These results underscore the critical role of the acrylohydrazide and benzothiazole functional groups in determining the biological efficacy of these compounds.

In contrast, compounds such as **7**, **10**, and **14** exhibited moderate antibacterial activity, with inhibition zones ranging from 3.65 to 3.9 cm. These compounds required higher concentrations to achieve complete bacterial eradication, as reflected in their relatively higher MIC and MBC values. Despite their lower efficacy compared to **6** and **13b**, these compounds still displayed significant antibacterial potential, demonstrating that the acrylonitrile and anthracene moieties, in combination with the heterocyclic rings, contribute to their antimicrobial effects.

The mode of action of the compounds varied, with some demonstrating bactericidal properties and others exhibiting bacteriostatic effects. Compounds like **7**, **11**, **13a**, and **15** were bactericidal, necessitating lower concentrations for complete bacterial death. In contrast, compounds such as **6**, **13b**, and **14** were found to be bacteriostatic, inhibiting bacterial growth at lower concentrations but requiring higher concentrations for full bacterial eradication.

Overall, the SAR analysis emphasizes the importance of the acrylonitrile and anthracene moieties, as well as the incorporation of heterocyclic structures, in achieving significant antimicrobial activity. These findings suggest that optimizing these structural components, alongside exploring further modifications to improve solubility and membrane permeability, could lead to the development of more effective anti-MRSA agents.

## Experimental

### Chemistry

All reagents and solvents were purified and dried using standard procedures (El Gomhouria Co., Egypt). Melting points were determined using a GallenKamp electric melting point apparatus and are reported uncorrected. Infrared (IR) spectra (cm⁻^1^) were recorded at the Chemistry Department, Faculty of Science, Ain Shams University, using potassium bromide (KBr) discs on a Thermo Electron Nicolet iS10 FTIR spectrometer (Thermo Fisher Scientific Inc., Waltham, MA, USA).

^1^H NMR spectra were acquired at 300 MHz using both a GEMINI NMR spectrometer (GEMINI Manufacturing & Engineering Inc., Anaheim, CA, USA) and a BRUKER 300 MHz NMR spectrometer (BRUKER Manufacturing & Engineering). Tetramethylsilane (TMS) served as the internal standard in deuterated dimethyl sulfoxide (DMSO-d₆). NMR measurements were conducted at the Faculty of Science, Cairo University, Giza, Egypt. Elemental analyses (CHN) were performed using a Perkin-Elmer 2400 elemental analyzer, and the obtained results were in good agreement with the calculated values.

#### 3-(Anthracen-9-yl)-2-cyanoacryloyl chloride **4**

A mixture of acid **3** (5 mmol) and thionyl chloride (5 mL) was heated on a water bath at 65 °C for 4 h. The excess thionyl chloride was distilled under reduced pressure. The obtained red solid was collected and used without further purification, mp. 200–202 °C, yield 88.5%. IR (KBr, ν, cm^−1^): 2224 (C≡N), 1737 (C=O). ^1^H-NMR (400 MHz, DMSO-d6) δ (ppm): 9.26 (s, 1H, CH=), 7.27–8.57 (m, 9H, H_aromatic_). ^13^C NMR (75 MHz, DMSO-d6) δ (ppm): 115.11 (C≡N), 124.82–130.53 (CH Anthracene ring), 155.34, 114.93 (CH=C–C=O), 162.22 (C=O); HRMS (ESI) m/z [M + H]^+^: 291.73111. Anal. Calcd. For C_18_H_10_ClNO (291.73): C, 74.11; H, 3.46; N, 4.80. Found: C, 74.41; H, 3.34; N, 4.66 %.

#### 3-(Anthracen-9-yl)-*N*′-(3-(anthracen-9-yl)acryloyl)-2-cyano-*N*′-phenylacrylohydrazide **6**

To a solution of **4** (2 mmol, 0.7 g) in dry dioxane (20 mL), hydrazine hydrate (2 mmol, 0.16 mL, 80%) was added dropwise at 0 °C, and the reaction mixture was stirred for an hour. The separated solid was collected by filtration and recrystallized from ethanol to give compound **6**.

Green crystals, mp. 190–192 °C, yield 69.36%; IR (KBr) ν = 3424 (NH), 2228 (C≡N), 1720 (C=O), 1621 (C=N) cm^−1^. ^1^H-NMR (400 MHz, DMSO-d6) δ (ppm): 9.3 (br.s, H, NH, exchangeable), 7.69–8.51 (m, 18H, H_aromatic_), 9.16 (s, 1H, CH=), 8.54 (s, 1H, CH=); Anal. Calcd. for C_33_H_21_N_3_O (475.53): C, 83.35; H, 4.45; N, 8.84. Found: C, 84.01; H, 4.38; N, 8.05%.

### General procedure for reaction of compound 4 with phenylhydrazine/2-aminopyridine

To a solution of **4** (2 mmol, 0.7 g) in dry dioxane (20 mL) containing a few drops of triethylamine, phenylhydrazine/2-aminopyridine (2 mmol) was added, and the reaction mixture was stirred at room temperature for 1 h and 2 h respectively. The reaction mixture was poured onto ice or water and acidified with dilute HCl. The precipitated solid was filtered off and recrystallized from ethanol to give **7** and from benzene to give **8**.

#### 3-(Anthracen-9-yl)-N′-(3-(anthracen-9-yl)-2-cyanoacryloyl)-2-cyano-*N*-phenylacrylohydrazide **7**

Green crystals, mp. 160–162 °C, yield 77%; IR (KBr, ν, cm^−1^): 3245 (NH), 2222 (C≡N), 1691 (C=O), 1671 (C=O) cm^−1^. ^1^H-NMR (400 MHz, DMSO-d6): δ (ppm): 10.88 (s, 1H, NH, exchangeable), 9.15 (s, 1H, CH=), 8.81 (s, 1H, CH=), 6.78–8.58 (m, 23H, H_aromatic_); Anal. Calcd. for C_42_H_26_N_4_O_2_ (618.68): C, 81.54; H, 4.24; N, 9.06. Found: C, 81.96; H, 3.91; N, 8.91%.

#### 3-(Anthracen-9-yl)-2-cyano-*N*-(pyridin-2-yl)acrylamide **8**

To a solution of **4** (2 mmol, 0.7 g) in dry dioxane (20 mL) containing a few drops of triethylamine, 2-aminopyridine (2 mmol, 0.25 g) was added, and the reaction mixture was stirred at room temperature for 2 h. The reaction mixture was poured onto ice/water and acidified with dilute HCl. The precipitated solid was filtered off and recrystallized from Benzene to give **8** as green crystals, mp. 146–148 °C, yield 82.5%; IR (KBr) ν: 3148 (NH), 2222 (C≡N), 1665 (C=O) cm^−1^. ^1^H-NMR (400 MHz, DMSO-d6): δ (ppm): 8.78 (s, H, NH, exchangeable), 9.14 (s, 1H, CH=), 7.36–8.55 (m, 13H, H_aromatic_); Anal. Calcd. for C_23_H_15_N_3_O (348.38): C, 79.07; H, 4.33; N, 12.03. Found: C, 78.93; H, 3.97; N, 12.36%.

### General procedure for reaction of compound 4 with thiourea/thiosemicarbazide

A mixture of **4** (2 mmol, 0.7 g) in dry dioxane (20 mL) containing few drops of triethylamine and thiourea/thiosemicarbazide (2 mmol) was heated under reflux for 2 h. The solvent was evaporated under vacuum. The residue was found to be a mixture of compounds recrystallized from dioxane.

#### 6-(Anthracen-9-yl)-1-(3-(anthracen-9-yl)-2-cyanoacryloyl)-4-hydroxy-2-thioxo-1,2,5,6-tetrahydropyrimidine-5-carbonitrile **9**

Green crystal, mp. 200–202 °C, yield 63%; IR (KBr) ν: 3472 (OH), 2231 (C≡N), 1711 (C=O) cm^−1^. ^1^H-NMR (400 MHz, DMSO-d6) δ (ppm): 9.39 (s, 1H, OH, exchangeable), 9.2 (s, H, CH=), 3 (t, H, CH–N), 3.4 (t, H, CH-CN), 7.69–8.54 (m, 18H, H_aromatic_); HRMS (ESI) m/z [M + H]^+^: 586.66116, Anal. Calcd. for C_37_H_22_N_4_O_2_S (586.66): C, 75.75; H, 3.78; N, 9.55. Found: C, 75.72; H, 3.89; N,9.61%.

#### *N*-(5-(-2-(anthracen-1-yl)-1-isocyanovinyl)-1,3,4-thiadiazol-2-yl)-3-(anthracen-9-yl)-2-cyanoacrylamide **10**

Green crystal, mp. 140–142 °C, yield 63%; IR (KBr) ν: 3378 (NH), 2227 (C≡N), 1710 (C=O). ^1^H-NMR (400 MHz, DMSO-d6) δ (ppm): 9.98 (s, 1H, NH, exchangeable), 9.2 (s, H, CH=), 9.3 (s, H, CH=), 7.70–8.55 (m, 18H, H_aromatic_). ^13^C NMR (75 MHz, DMSO-d6) δ (ppm): 114.89 (C≡N), 107.31 (C≡N), 107.05, 154.82 (CH=**C**–CN), 124.49–128.86 (C– Anthracene ring), 147.86, 148.36 (S–C=N (thiazole ring)), 164.45(C=O); HRMS (ESI) m/z [M + H]^+^: 583.66053. Anal. Calcd. for C_37_H_21_N_5_OS (583.66): C, 76.14; H, 3.63; N, 12.00. Found: C, 78.42; H, 3.94; N,12.35%.

#### 6-(Anthracen-9-yl)-1,2,4,5-tetrazine-3-thiol **11**

A solution of **4** (2 mmol, 0.7 g) and Thiocarbohydrazide (2 mmol, 0.26 g) in dry dioxan (20 mL) containing two drops of triethylamine was heated under reflux for 1 h. The precipitated solid upon cooling was collected and recrystallized from benzene to give **11**, Orange crystal, mp. 200–203 °C, yield 84%; IR (KBr) ν: 3077 (CH aromatic), 1619 (C=N) cm^−1^. ^1^H-NMR (400 MHz, DMSO-d6): δ (ppm): 3.31 (s, 1H, SH, exchangeable), 7.78–8.66 (m, 9H, H_aromatic_). ^13^C NMR (75 MHz, DMSO-d6) δ (ppm): 149.67 (SH-C = N-tetrazine), 124.76–128.50 (C– Anthracene ring); Anal. Calcd. for C_16_H_10_N_4_S (290.34): C, 66.19; H, 3.47; N, 19.30. Found: C, 66.32; H, 3.24; N, 19.47%.

#### 3-(Anthracen-9-yl)-2-(1H-benzo[d]imidazol-2-yl)acrylonitrile **13a**

A solution of **4** (2 mmol, 0.7 g) and 1,2-Phenylenediamine (2 mmol, 0.3 g) in dry dioxane (20 mL) containing two drops of triethylamine was heated under reflux for 1 h. The precipitated solid upon cooling was collected and recrystallized from dioxane to give **13a** as green crystal, mp. 260–264 °C, yield 78%; IR (KBr) ν:3267 (NH), 2222 (C≡N), 1638 (C=N) cm^−1^. ^1^H-NMR (400 MHz, DMSO-d6) δ (ppm): 10.65 (s, 1H, NH, exchangeable), 9.23 (s, 1H, CH=), 6.76–8.40 (m, 13H, Ar–H); Anal. Calcd. for C_24_H_15_N_3_ (345.39): C, 83.46; H, 4.38; N, 12.17. Found: C, 83.27; H, 3.99; N, 12.32%.

#### 3-(Anthracen-9-yl)-2-(benzo[d]thiazol-2-yl)acrylonitrile (**13b**)

For one hour, reflux was used to heat a solution of **4** (2 mmol, 0.7 g) and 2-aminothiophenol (2 mmol, 0.3 g) in dry dioxane (20 mL) with two drops of triethylamine After cooling, the precipitated material was gathered and recrystallized from benzene to yield **13b**. Yellow crystal, mp. 180–182 °C, yield 66%; IR (KBr) ν: 2222 (C≡N), 1606 (C=C) cm^−1^. ^1^H-NMR (400 MHz, DMSO-d6) δ (ppm): 9.30 (s, 1H, CH=), 7.35–8.52 (m, 13H, H_aromatic_) ppm.^13^C NMR (75 MHz, DMSO-d6) δ (ppm): 114.65 (C≡N), 126.43–130.65 (C– Anthracene ring), 124.82–126.05 (C– benzene ring), 160.64 (C=N (thiazole ring)), 155.51 (C=**CH**); Anal. Calcd. for C_24_H_14_N_2_S (362.45): C, 79.53; H, 3.89; N, 7.73. Found: C, 79.86; H, 3.75; N, 7.51%.

#### 3-(Anthracen-9-yl)-2-(2-oxo-2H-benzo[b][1,4]oxazin-3-yl)acrylonitrile **14**

A solution of **4** (2 mmol, 0.7 g) and 2-aminobenzoicacid (2 mmol, 0.29 g) in dry dioxane (20 mL) containing two drops of pyridine was refluxed for 3 h. The precipitated solid was collected recrystallized from ethanol to give **14.** Green crystals, mp. 195–197 °C, yield 78%; IR (KBr) ν: 2220 (C≡N), 1620 (C=N), 1637 (C=O) cm^−1^. ^1^H-NMR (400 MHz, DMSO-d6) δ (ppm): 9.20 (s, 1H, CH), 7.36–8.59 (m, 13H, H_aromatic_); HRMS (ESI) m/z [M + H]^+^: 374.39087, Anal. Calcd. for C_25_H_14_N_2_O_2_ (374.39): C, 80.20; H, 3.77; N, 7.48. Found: C, 79.96; H, 3.97; N, 7.21%.

### General procedure for reaction of compound 4 with 1,2-diaminoethane/2-aminoethanol

1,2-diaminoethane (2 mmol, 0.15 mL) or 2-aminoethanol (2 mmol, 0.14 mL) were added to a stirred solution of acid chloride 4 (2 mmol, 0.7 g) in dry dioxane (10 mL) containing two drops of triethylamine, and stirring was continued for 10 min. Compounds **15** and **16** were obtained respectively by gathering and recrystallizing the precipitated solid in the appropriate solvents.

#### 5-(Anthracen-9-yl)-7-hydroxy-5,6-dihydro-2H-1,4-diazepine-6-carbonitrile **15**

Compound 1,4-diazepine **15** as Pale-yellow crystals, mp. 198–200 °C (benzene), yield 77%. IR (KBr) ν: 3404 (OH), 2222 (C≡N), 1628 (C=N) cm^−1^. ^1^H-NMR (400 MHz, DMSO-d6) δ (ppm): 7.68–8.50 (m, 9H, H_aromatic_), 8.53 (d, 2H, CH_2_-N), 6.2 (d, 2H, CH_2_-CN) 8.48 (br.s, 1H, OH, exchangeable), 6.6 (d, 2H, CH_2_), 3.36 (t, H, CH=N); HRMS (ESI) m/z [M + H]^+^: 313.35261, Anal. Calcd. for C_20_H_15_N_3_O (313.35): C, 76.66; H, 4.83; N, 13.41. Found: C, 76.71; H, 5.09; N, 13.20%.

#### 4-(Anthracen-9-yl)-2,5-dihydrooxazole **16**

Yellow crystals, mp. 196–198 °C (dioxane), yield 88%. IR (KBr) ν: 2919 (C-H ali), 1618(C=N) cm^−1^. ^1^H-NMR (400 MHz, DMSO-d6) δ (ppm): 7.67–8.68 (m, 9H, H_aromatic_), 2.87 (s, 2H, CH_2_-O), 1.20 (s, 2H, CH_2_-N); HRMS (ESI) m/z [M + H]^+^: 247.29122, Anal. Calcd. For C_17_H_13_NO (247.29): C, 82.57; H, 5.30; N, 5.66. Found: C, 82.72; H, 4.95; N, 5.79%.

#### 3-(Anthracen-9-yl)-2-cyano-*N*-(2-hydroxyphenyl)acrylamide **17**

A solution of **4** (2 mmol, 0.7 g) and 2-aminophenol (2 mmol, 0.26 g) in dry dioxane (20 mL) containing two drops of triethylamine was refluxed for 1 h. The precipitated solid was collected and recrystallized from ethanol to give **17.** Green crystals, mp. 260–262 °C, yield 65%.; IR (KBr) ν: 3419 (OH), 3377 (NH), 2226 (C≡N), 1681 (C=O) cm^−1^. ^1^H-NMR (400 MHz, DMSO-d6) δ (ppm): 9.97 (br.s, 1H, NH, exchangeable), 9.82 (br.s, 1H, OH, exchangeable), 9.19 (s, 1H, CH=), 6.87–8.56 (m, 13H, H_aromatic_); Anal. Calcd. for C_24_H_16_N_2_O_2_ (364.39): C, 79.11; H, 4.43; N, 7.69. Found: C, 79.41; H, 4.27; N, 7.94%.

### General procedure for reaction of compound 4 with primary amines

To a stirred solution of acid chloride 4 (1 g, 3 mmol) in dry dioxane (10 mL) containing 2-drops of triethylamine, the suitable primary amines, namely ethyl amine, p-toluidine and 4-nitroanisol (3 mmol) were added at room temperature. The reaction mixture was further stirred for 1 h. The precipitated solid was sucked and recrystallized from the suitable solvent to produce the compounds **18**, **9 (a**, **b)** respectively.

#### 3-(Anthracen-9-yl)-2-cyano-*N*-ethylacrylamide **18**

Green crystals; m.p 200–203 °C; IR (KBr) ν: 3411 (NH), 2217 (CN), 1684 (C=O). ^1^H-NMR (400 MHz, DMSO-d6) δ (ppm): 9.18 (s, H, CH=), 7.22–8.57 (m, 9H, H_aromatic_), 3.30 (s, 3H, CH_3_), 1.4 (q, 2H, CH_2_), 10.67 (s, H, NH, exchangeable) ppm. ^13^C NMR (75 MHz, DMSO-d6) δ (ppm): 114.82 (C≡N), 120.72–135.69 (CH Anthracene ring), 149.12, 94.31 (**C**H=**C**–C=O), 159.12 (C=O), 38.70 (NH-CH_2_), 20.58 (**C**H_3_-CH_2_); HRMS (ESI) m/z [M + H]^+^: 300.35383, Anal. Calcd for C_20_H_16_N_2_O (300.35) %: C, 79.98; H, 5.37; N, 9.33; found: C, 79.78; H, 4.98; N, 9.45.

#### 3-(Anthracen-9-yl)-2-cyano-*N*-(p-tolyl)acrylamide **19a**

Green crystals; m.p 200–202 °C; IR (KBr) ν: 3411 (NH), 2218 (CN), 1684 (C=O) cm^−1^. ^1^H-NMR (400 MHz, DMSO-d6) δ (ppm): 9.13 (s, H, CH =), 7.72–8.55 (m, 13H, H_aromatic_), 3.30 (s, 3H, CH_3_), 7.82 (s, H, NH, exchangeable); Anal. Calcd for C_25_H_18_N_2_O (362.42) %: C, 82.85; H, 5.01; N, 7.73; found: C, 82.96; H, 4.88; N, 7.70.

#### 3-(Anthracen-9-yl)-2-cyano-*N*-(4-methoxyphenyl)acrylamide **19b**

Green crystals; m.p 170–173 °C; IR (KBr) ν: 3423 (NH), 2223 (CN), 1678 (C=O) cm^−1^. ^1^H-NMR (400 MHz, DMSO-d6) δ (ppm):9.23 (s, H, CH=), 6.50–8.80 (m, 13H, H_aromatic_), 3.6(s, 3H, OCH_3_), 6.48 (s, H, NH, exchangeable); Anal. Calcd for C_25_H_18_N_2_O_2_ (378.42) %: C, 79.35; H, 4.79; N, 7.40; found: C, 79.40; H, 4.98; N, 6.95.

### Antimicrobial activity

#### Screening of the antibacterial activity

The qualitative agar well diffusion method is used for evaluating the antibacterial activity of the different thirteen synthesized compounds according to CLSI^57^. The agar plate surface is inoculated with 0.5 Maccfaeland bacterial suspension from an overnight culture of the tested susceptible bacteria [*Staphylococcus aureus* ATCC 29,213 (MRSA) and *E. coli* (local pathogenic isolate)]. Afterwards, holes with a diameter of 10 mm are punched aseptically with a sterile cork borer within the agar plates. A volume of 100 μL from each chemical compound at concentration of 560 μg/100μL DMSO is injected into each separate well. After the diffusion in the agar medium at 4 °C, the agar plates are incubated for 24 h at the appropriate temperature. The experiment is carried out in duplicate. Finally, the assessed inhibition zone is measured and the mean values for replica are recorded.

#### Determination of minimum inhibitory concentrations of the active compounds

The minimum inhibitory concentration (MIC) for each active tested compound is investigated through a micro dilution broth assay in a sterile 96-well microtiter plate^58,59^. Ten different concentrations are prepared for each compound using DMSO to prepare two-fold dilutions starting with 625 μg/100 μL till 9.7 μg/100 μL in two rows in the plate. The wells of one row are inoculated with 5 µL from an overnight culture of MRSA (0.5Mcfarland) while the wells of the other row are not inoculated with the test bacteria to serve as a control. The final volume in each well is 100 µL and each sample with its control are prepared in duplicates. Positive control (the growth medium inoculated with the test bacteria) and a negative control (growth medium only) are included in the microtiter plate. The plate is incubated for 24 h at 37 °C. After the incubation period, the growth is checked in each well of the microtiter plates by the unaided eye in comparison with the control row of each tested compound to determine the visualized MIC. The MIC is defined as the lowest concentration of the tested compound that inhibits the visible growth of the tested microorganism with the naked eye^22–25^. For further confirmation of MIC, the minimum bactericidal concentration (MBC) is determined by subculturing an aliquot of 10μL from each mixture to nutrient agar plates.

The plates are then incubated for 24 h at 37 °C^59^. The experiment was carried out in duplicate. The bacterial growth after the incubation indicates the cell viability. Absence of bacterial growth on the plates indicates the killing of the tested microbes. MBC is the lowest concentration of an antibacterial agent required to kill a bacterium.

#### Tolerance level determination

Determining the effect of each compound on the tested bacteria whether to be bacteriostatic or bactericidal is achieved by calculating the tolerance level for the tested bacteria towards the different tested compounds. The tolerance level reported according to the ratio between MBC and MIC; where, If the ratio is > 4, then the agent has a bacteriostatic effect whereas the ratio ≤ 4 reflects the bactericidal ability of the tested compound^60^.

#### Molecular docking simulations

Molecular docking studies were performed to investigate the interactions between the synthesized compounds and Penicillin-Binding Protein 2a (PBP2a) from Methicillin-Resistant *Staphylococcus aureus* (MRSA), utilizing the crystal structure available under PDB ID 4CJN.

##### Protein preparation

The 3D structure of PBP2a, complexed with a quinazolinone ligand, was retrieved from the Protein Data Bank (PDB). Prior to docking, the protein was prepared by removing all water molecules and unrelated ligands, except for the quinazolinone ligand, which was retained to define the active site. The preparation also involved adding hydrogen atoms and performing energy minimization using the Molecular Operating Environment (MOE) software (version 2024.06).

##### Ligands preparation

The 2D structures of the compounds were initially drawn in ChemDraw (version 20.0) and imported into MOE, where they were converted into 3D models. Subsequently, protonation states were assigned, partial charges were calculated, and energy minimization was carried out to optimize the ligands for docking simulations.

##### Docking procedure

Molecular docking simulations were performed using the Molecular Operating Environment (MOE) software on Penicillin-Binding Protein 2a (PBP2a), with the protein treated as a rigid body to maintain a stable receptor framework throughout the simulation. The ligands were made flexible, allowing them to adapt their conformation for optimal fitting into the enzyme’s active site.

The active site for docking was precisely defined by the position of the co-crystallized quinazolinone ligand within the PBP2a structure. This strategic placement ensures accurate targeting of the enzyme’s biological hotspot. During the docking simulations, 100 initial poses were generated for each compound to thoroughly explore potential binding configurations. From these, the top 20 poses were selected based on their binding scores, with particular emphasis on the London ΔG scoring function provided by MOE, which estimates the free energy change upon binding^61–65^.

To identify the most optimal pose for each compound, additional evaluations were carried out, focusing on the lowest binding energy (S-score), a standard MOE metric for estimating ligand binding efficiency. Poses with a root mean square deviation (RMSD) value of less than 2 Å from the initial ligand position were prioritized to ensure accurate alignment with the co-crystallized ligand. This step is essential for confirming the biological relevance of the ligand interactions observed in the simulations.

Finally, the optimal binding mode for each compound was selected based on the similarity of its interactions with the co-crystallized ligand. This involved evaluating key interactions, such as hydrogen bonds, ionic interactions, and π-stacking, which are essential for robust and specific binding to the target protein. This thorough approach ensures that only the most promising compounds are prioritized for further experimental validation and development as potential antimicrobial agents.

##### Validation of docking simulations

To validate the docking results, the co-crystallized quinazolinone ligand was redocked into the active site of PBP2a. This step was essential for comparing the binding mode and energy scores of the tested compounds with those of the co-crystallized ligand, ensuring that the docking simulations accurately represented potential binding interactions. The comparative analysis further supports the reliability of the docking predictions and evaluates the potential efficacy of the compounds as antimicrobial agents.

## Conclusion

In this study, a new series of heterocyclic compounds incorporating anthracene and acrylonitrile moieties were synthesized, characterized, and evaluated for their antimicrobial activity. The biological screening revealed that ten of the synthesized compounds demonstrated potent antibacterial effects against MRSA, with compounds **6** and **13b** exhibiting the strongest inhibition zones (4 cm) and lowest MIC values (9.7 μg/100 μL). Molecular docking analysis confirmed that several compounds, particularly **7**, **9**, **10**, and **14**, exhibited strong binding interactions with PBP2a, suggesting their potential role as enzyme inhibitors. While docking scores correlated well with some antimicrobial activity data, certain compounds exhibited high biological activity despite moderate docking scores, indicating that additional factors such as solubility, membrane permeability, and bioavailability may influence their effectiveness.

The findings underscore the potential of these novel heterocyclic compounds as PBP2a inhibitors and anti-MRSA candidates, warranting further in-depth studies, including pharmacokinetic profiling, cytotoxicity assessment, and in vivo validation. Future research will focus on optimizing these structures to enhance their binding affinity, bioavailability, and overall therapeutic potential against resistant bacterial strains.

## Supplementary Information

Supplementary Information.

## Data Availability

The data that support the findings of this study are available from the corresponding author Hassaballah, A.I. on reasonable request.
