# Supplementary material for: Design synthesis, characterization, molecular docking and antimicrobial evaluation of novel heterocycles with acrylonitrile and anthracene moieties
Source: Sci Rep. 2025 Jun 3;15:19370. doi: 10.1038/s41598-025-03272-5 (PMC12134333; doi:10.1038/s41598-025-03272-5)
Supplement: Supplementary file 1 — Supplementary Information. [file 41598_2025_3272_MOESM1_ESM.pdf]

Design Synthesis, Characterization, Molecular Docking and Antimicrobial Evaluation of Novel Heterocycles with Acrylonitrile and Anthracene Moieties

Aya. I. Hassaballah[1*], A. K. El-ziaty[1*], Marwa M. Gado[2], Hayam A. E. Sayed[2], Mahmoud Kamal[3], and Rania S. Ali[4]

[1] Department of Chemistry, Faculty of Science, Ain Shams University, Abbassia, 11566, Cairo, Egypt.

[2] Department of Microbiology, Faculty of Science, Ain Shams University, Abbassia, 11566, Cairo, Egypt.

[3] Department of Entomology, Faculty of Science, Ain Shams University, Abbassia, 11566, Cairo, Egypt.

[4] Department of Basic Science, Faculty of Technology and Education, Helwan University, Cairo, Egypt

Fax: +(202) 24831836; phone: +(202) 24831836.

***Corresponding author; e-mail: ayaibrahim@sci.asu.edu.eg**

Molecular docking:

Comprehensive molecular docking analysis of 15 synthesized compounds against Penicillin-Binding Protein 2a (PBP2a) in Methicillin-Resistant *Staphylococcus aureus* (MRSA), compared to the co-crystallized quinazolinone ligand (CCL). This file includes 2D interaction diagrams illustrating key molecular interactions, such as hydrogen bonding, π-stacking, and ionic interactions with active site residues. Additionally, a detailed table presents interaction types, interacting residues, bond energy (kcal/mol), and bond lengths (Å), providing valuable insights into the binding affinities and inhibitory potential of these compounds.

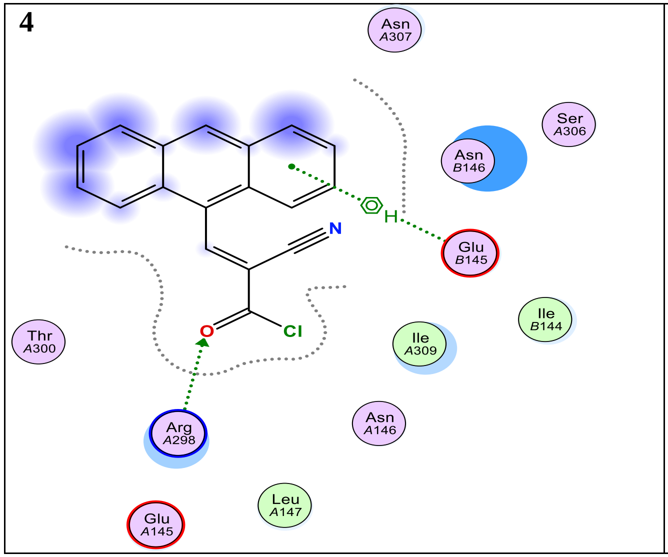
4
Asn A307
Ser A306
Asn B146
N
H
Glu B145
O
Cl
Thr A300
Ile A309
Ile B144
Arg A298
Asn A146
Leu A147
Glu A145

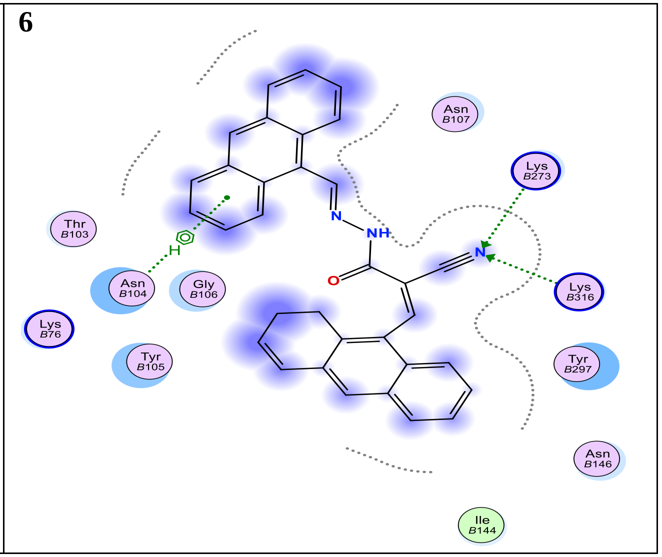
6
Asn B107
Lys B273
N
NH
Thr B103
H
N
O
Asn B104
Gly B106
Lys B316
Lys B76
Tyr B105
Tyr B297
Asn B146
Ile B144

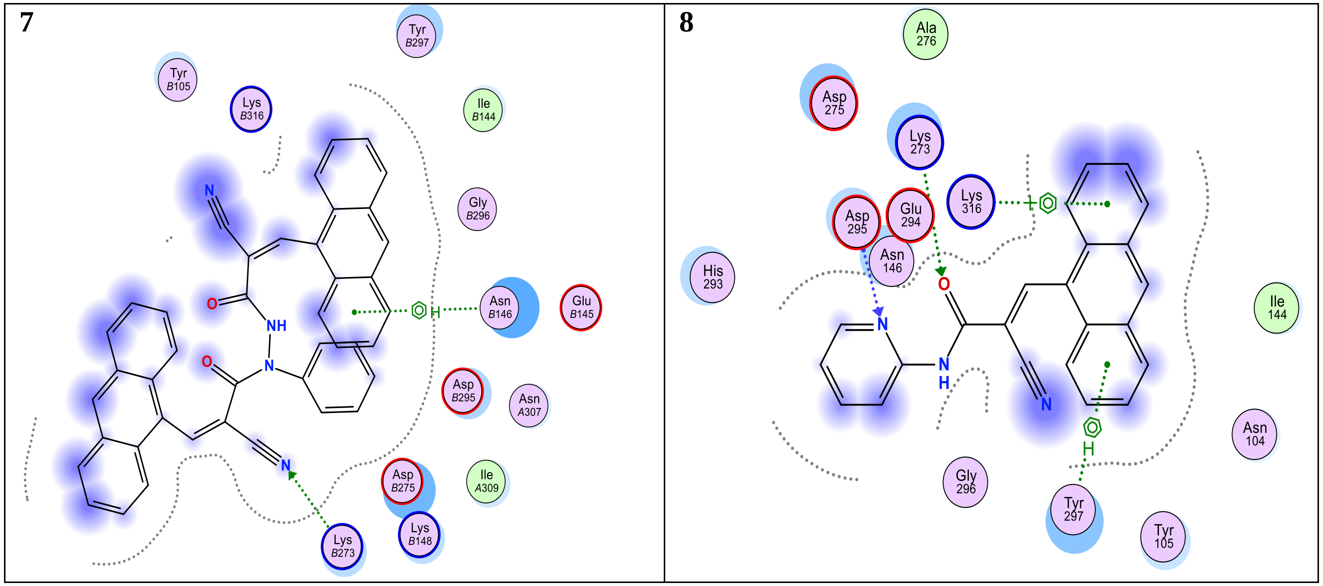
7
Tyr B297
Tyr B105
Lys B316
Ile B144
Gly B296
N
O
NH
O
N
H
Asn B146
Glu B145
Asp B295
Asn A307
N
Asp B275
Ile A309
Lys B148
Lys B273
8
Ala 276
Asp 275
Lys 273
Lys 316
Asp 295
Glu 294
Asn 146
His 293
O
N
N H
N
Ile 144
Asn 104
H
Gly 296
Tyr 297
Tyr 105

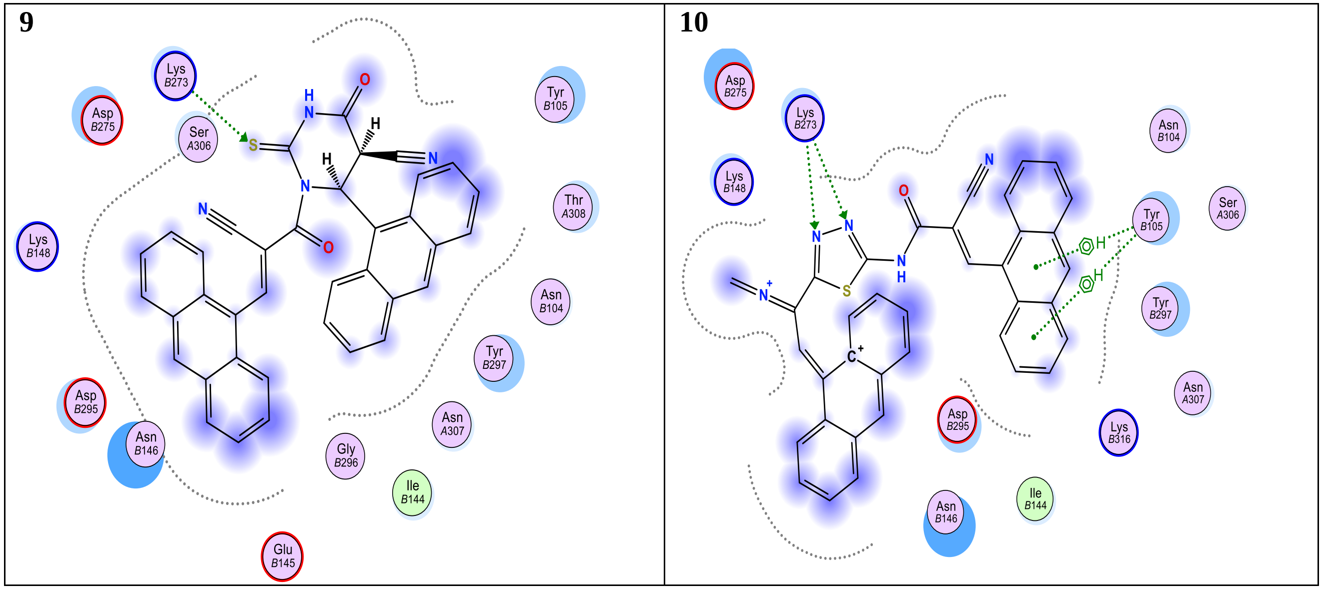

9
Lys B273
Asp B275
Ser A306
Lys B148
Asp B295
Asn B146
Glu B145
Gly B296
Ile B144
Asn A307
Tyr B297
Asn B104
Thr A308
Tyr B105
10
Asp B275
Lys B273
Lys B148
Asn B104
Ser A306
Tyr B105
Tyr B297
Asn A307
Lys B316
Asp B295
Asn B146
Ile B144

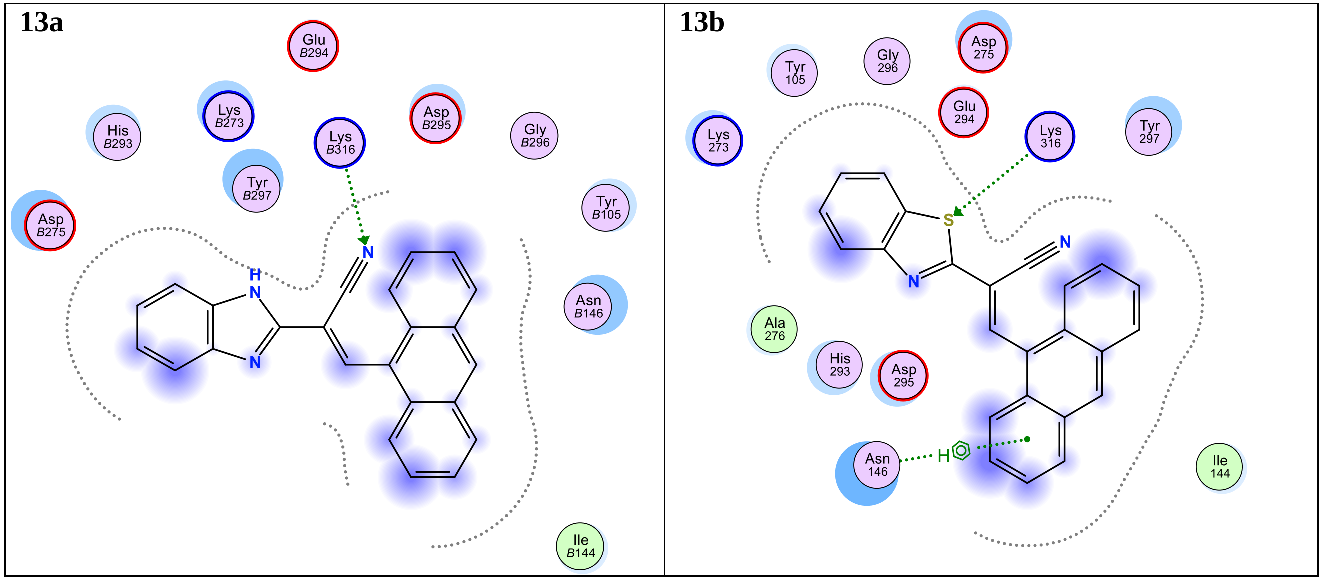
13a
Glu B294
Lys B273
Lys B316
Asp B295
His B293
Gly B296
Tyr B297
Asp B275
Tyr B105
H
N
N
N
Asn B146
Ile B144
13b
Tyr 105
Gly 296
Asp 275
Glu 294
Lys 316
Tyr 297
Lys 273
S
N
N
Ala 276
His 293
Asp 295
Asn 146
H
Ile 144

14

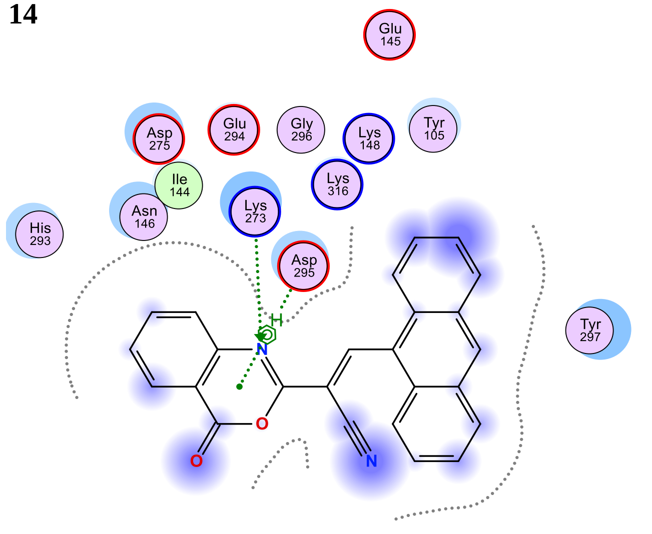
Glu 145
Asp 275
Glu 294
Gly 296
Lys 148
Tyr 105
Ile 144
Lys 316
His 293
Asn 146
Lys 273
Asp 295
Tyr 297
N
H
O
O
N

15

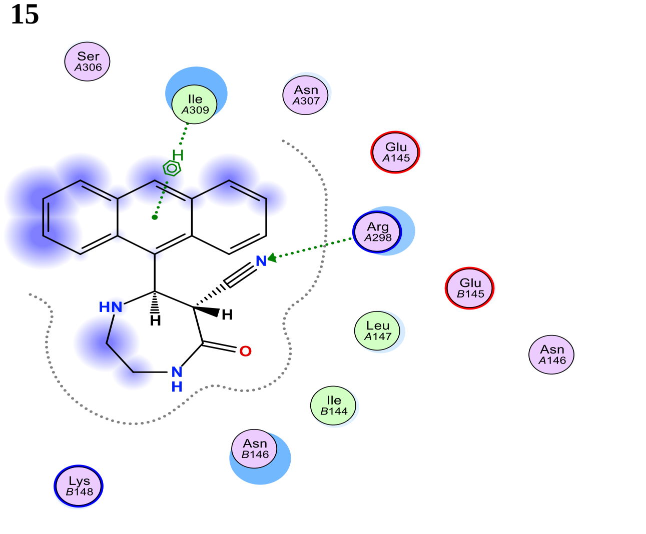
Ser A306
Ile A309
Asn A307
Glu A145
Arg A298
Glu B145
Leu A147
Asn A146
Ile B144
Asn B146
Lys B148
H
N
HN
H
H
O
N
H

16

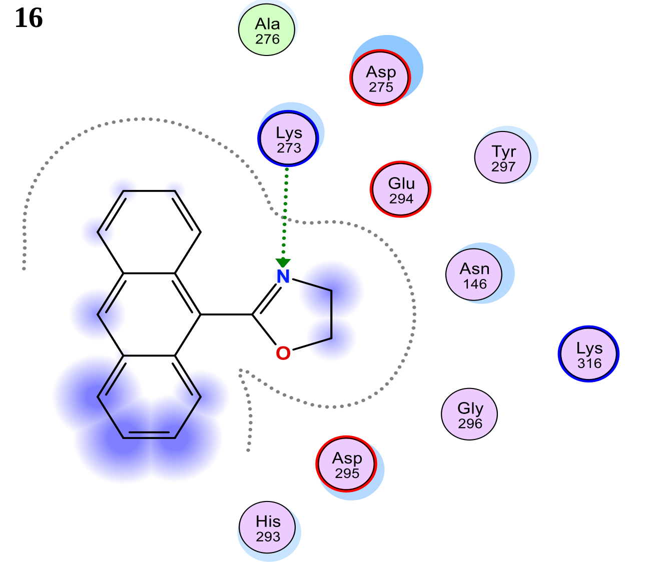
Ala 276
Asp 275
Lys 273
Glu 294
Tyr 297
N
O
Asn 146
Lys 316
Gly 296
Asp 295
His 293

17

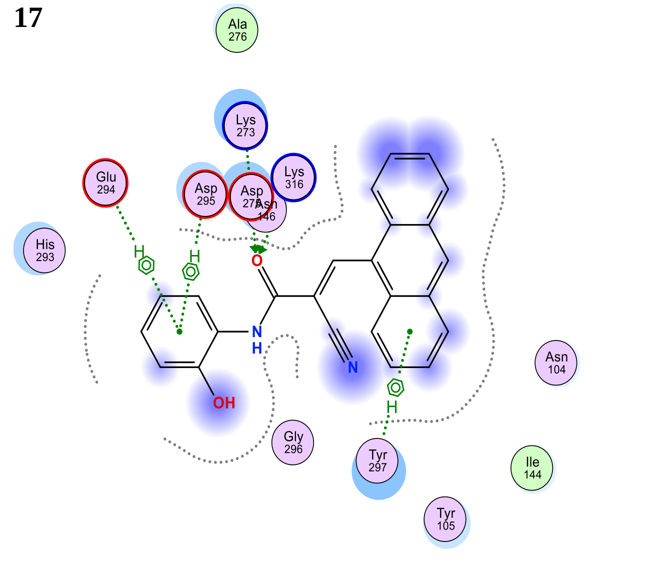
Ala 276
Lys 273
Glu 294
Asp 295
Asp 275
Asn 146
Lys 316
His 293
O
N
H
N
OH
Gly 296
Tyr 297
Asn 104
Ile 144
Tyr 105

18

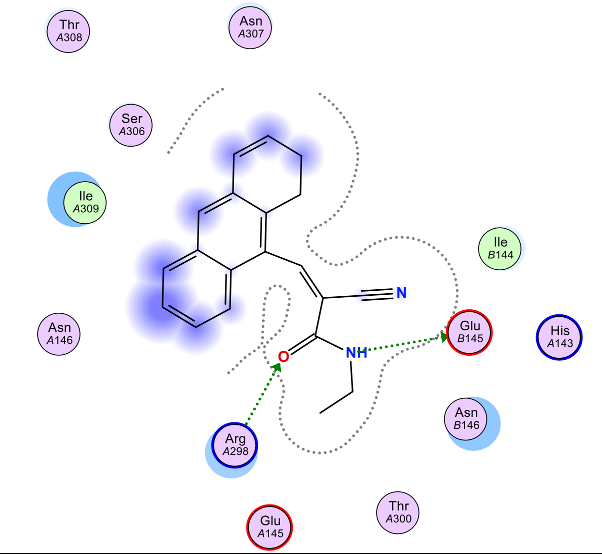

Thr A308
Asn A307
Ser A306
Ile A309
Ile B144
Asn A146
Glu B145
His A143
Asn B146
Arg A298
Glu A145
Thr A300
N
O
NH

19a

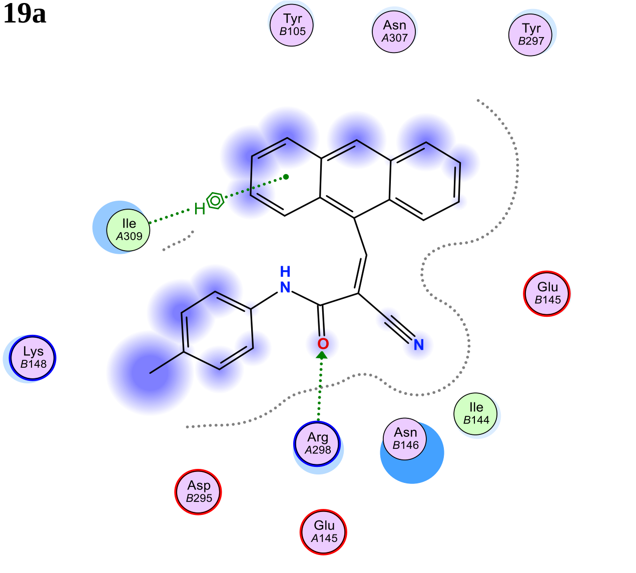

Tyr B105
Asn A307
Tyr B297
Ile A309
H
Glu B145
H N
Lys B148
O
N
Ile B144
Arg A298
Asn B146
Asp B295
Glu A145

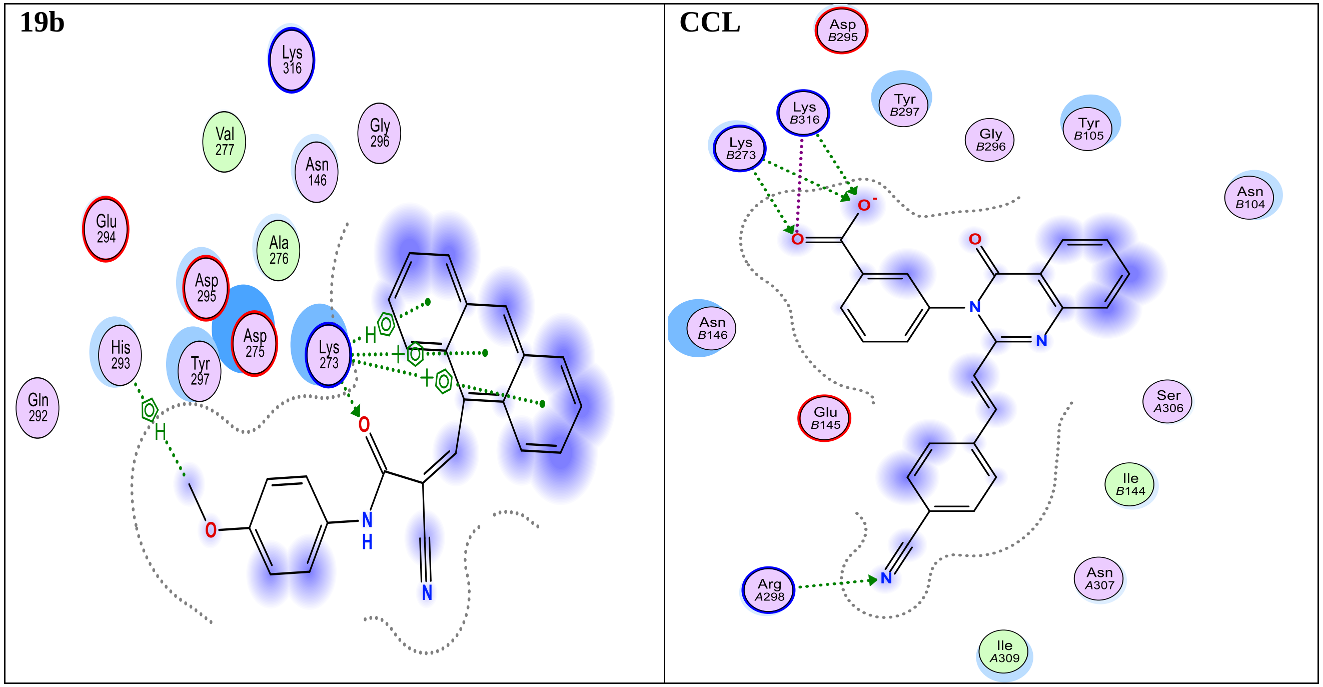
19b
Lys 316
Val 277
Gly 296
Asn 146
Glu 294
Ala 276
Asp 295
Asp 275
His 293
Tyr 297
Lys 273
Gln 292
H
O
N
H
N
O
CCL
Asp B295
Tyr B297
Lys B316
Lys B273
Gly B296
Tyr B105
Asn B104
O-
O
O
N
N
Asn B146
Glu B145
Ser A306
Ile B144
Arg A298
N
Asn A307
Ile A309

Ligand	Ligand interacting atoms	Residues interacting atoms	Interacting Residues	Interaction Type	Distance (Å)	E (kcal/mol)
4	O 7	NE	ARG 298 (A)	H-acceptor	3	-1.2
	6-ring	CA	GLU 145 (B)	pi-H	4.7	-0.8
6	N 60	CE	LYS 273 (B)	H-acceptor	3.6	-1.1
	N 60	NZ	LYS 316 (B)	H-acceptor	3.25	-7.5
	6-ring	CA	ASN 104 (B)	pi-H	3.96	-0.6
7	N 74	NZ	LYS 273 (B)	H-acceptor	3.07	-2.1
	6-ring	CB	ASN 146 (B)	pi-H	4.61	-0.5
8	N 5	N	ASP 295 (B)	H-acceptor	3.25	-2.4
	O 13	NZ	LYS 273 (B)	H-acceptor	3.03	-1.6
	6-ring	CB	TYR 297 (B)	pi-H	3.96	-0.5
	6-ring	NZ	LYS 316 (B)	pi-cation	5.26	-0.5
9	S 15	NZ	LYS 273 (B)	H-acceptor	3.29	-6.5
10	N 2	NZ	LYS 273 (B)	H-acceptor	2.89	-0.7
	N 3	NZ	LYS 273 (B)	H-acceptor	3.04	-0.6
	6-ring	CE1	TYR 105 (B)	pi-H	4.75	-0.6
	6-ring	CE1	TYR 105 (B)	pi-H	3.69	-0.8
13a	N 42	NZ	LYS 316 (B)	H-acceptor	3.04	-0.6
13b	S 11	NZ	LYS 316 (B)	H-acceptor	3.93	-1.3
	6-ring	CB	ASN 146 (B)	pi-H	3.71	-0.9
14	N 11	NZ	LYS 273 (B)	H-acceptor	2.96	-1.4

	6-ring	CB	ASP 295 (B)	pi-H	3.62	-0.9
15	N 41	NE	ARG 298 (A)	H-acceptor	3.44	-2.9
	6-ring	CG1	ILE 309 (A)	pi-H	3.68	-0.5
16	N 1	NZ	LYS 273 (B)	H-acceptor	3.24	-3.3
17	O 5	NZ	LYS 273 (B)	H-acceptor	3.04	-1.5
	O 5	NZ	LYS 316 (B)	H-acceptor	3.23	-0.9
	6-ring	CA	GLU 294 (B)	pi-H	4.76	-0.6
	6-ring	N	ASP 295 (B)	pi-H	4.42	-0.7
	6-ring	CB	TYR 297 (B)	pi-H	4	-0.5
18	N 9	OE1	GLU 145 (B)	H-donor	3.23	-3.5
	O 39	NE	ARG 298 (A)	H-acceptor	3.03	-3.4
	O 39	NH2	ARG 298 (A)	H-acceptor	2.86	-4.9
19a	O 6	NH2	ARG 298 (A)	H-acceptor	2.98	-0.7
	6-ring	N	ILE 309 (A)	pi-H	4.49	-1
19b	O 6	NZ	LYS 273 (B)	H-acceptor	2.99	-4.7
	C 44	5-ring	HIS 293 (B)	H-pi	3.81	-0.7
	6-ring	CE	LYS 273 (B)	pi-H	3.4	-0.6
	6-ring	NZ	LYS 273 (B)	pi-cation	4.68	-1.3
	6-ring	NZ	LYS 273 (B)	pi-cation	3.66	-1.3
CCL	N 1	NH2	ARG 298 (A)	H-acceptor	3.11	-5.8
	O 30	CE	LYS 273 (B)	H-acceptor	3.18	-0.7
	O 30	NZ	LYS 316 (B)	H-acceptor	2.92	-4.1
	O 31	NZ	LYS 273 (B)	H-acceptor	3.02	-10.8

	**O 30**	**NZ**	**LYS 273 (B)**	**Ionic**	**3.06**	**-4.1**
	**O 30**	**NZ**	**LYS 316 (B)**	**Ionic**	**2.92**	**-5**

Full spectroscopic data

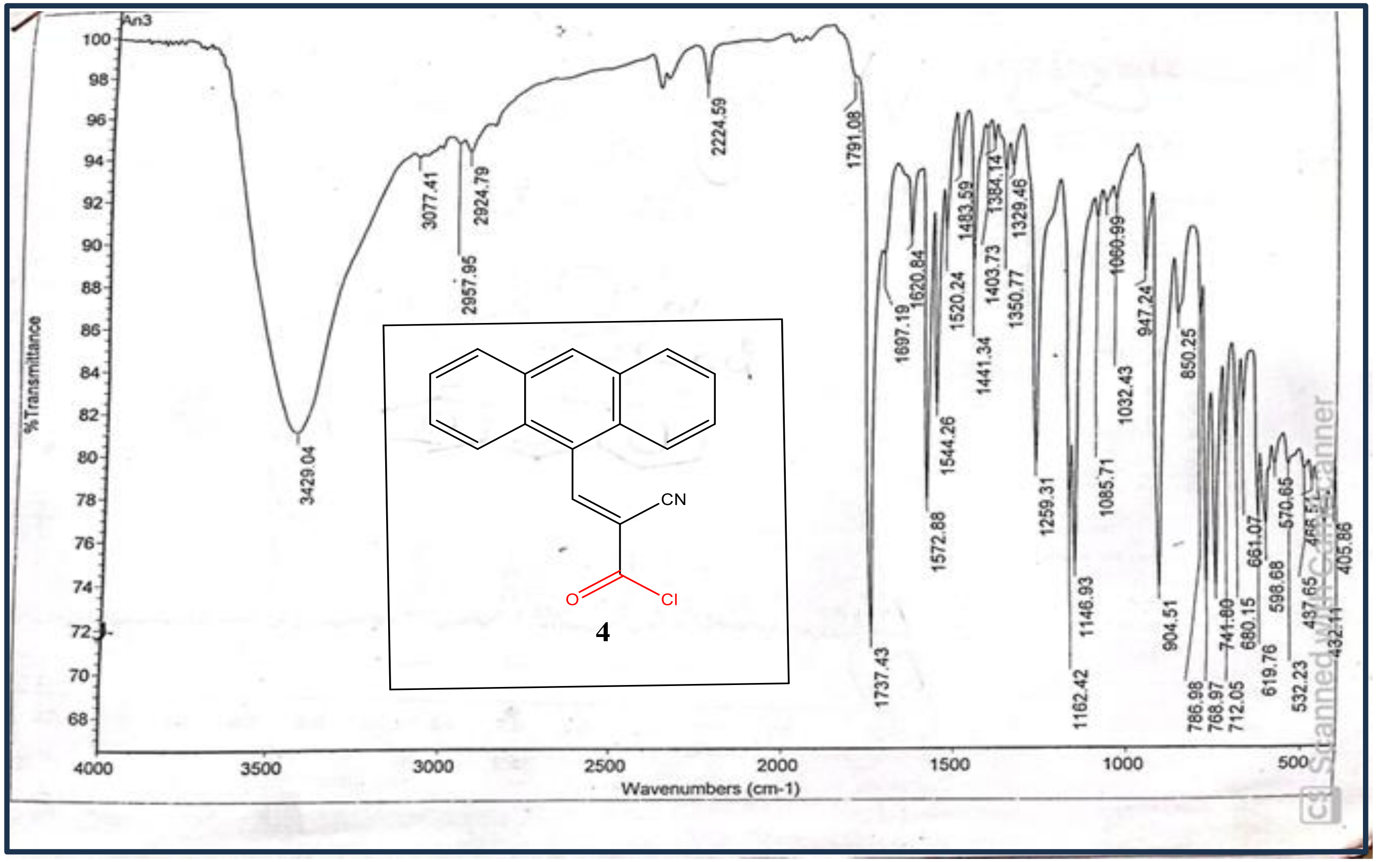

Figure S1. IR spectrum of compound **4**

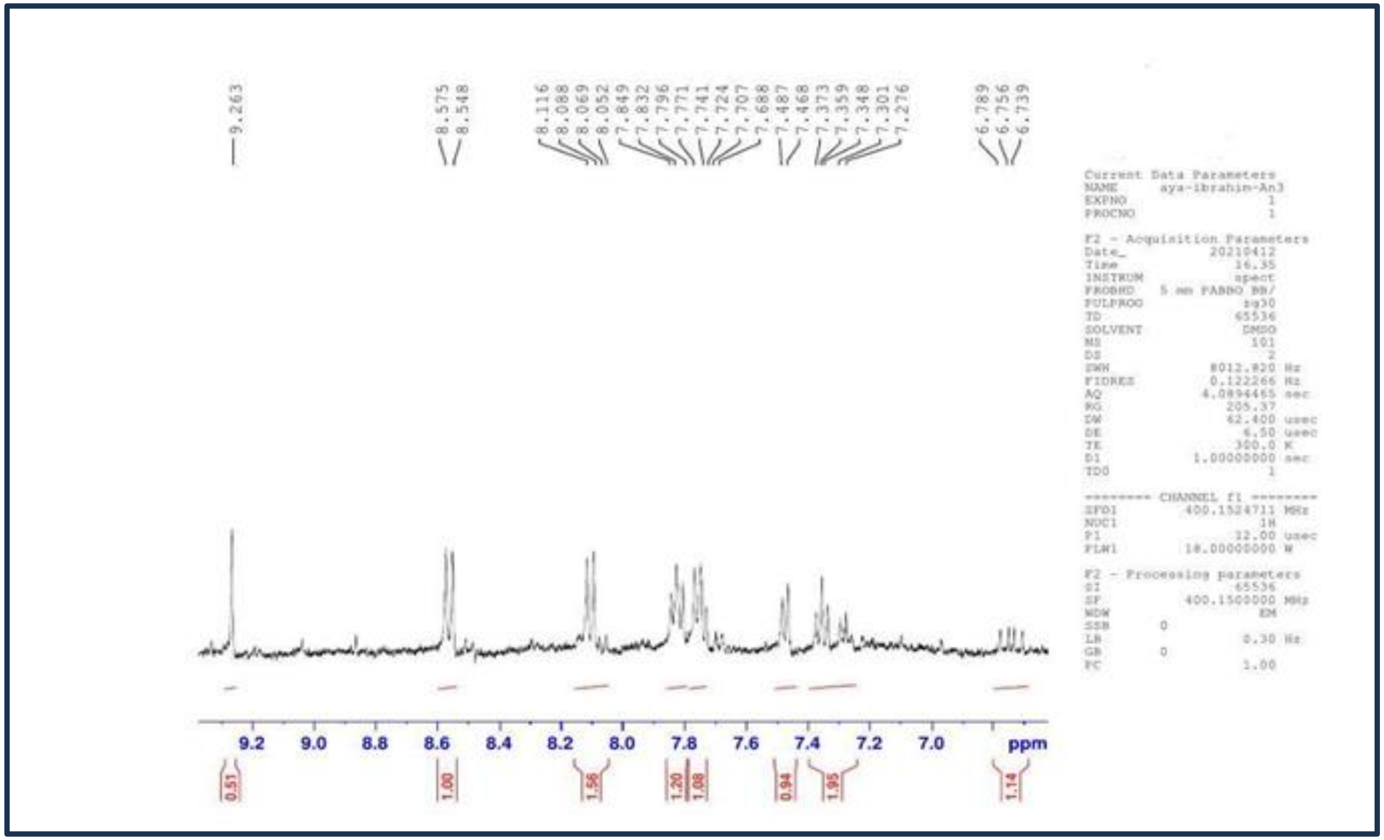

Figure S2. ^{1}H NMR (300 MHz, DMSO-d_6) spectrum of compound **4**

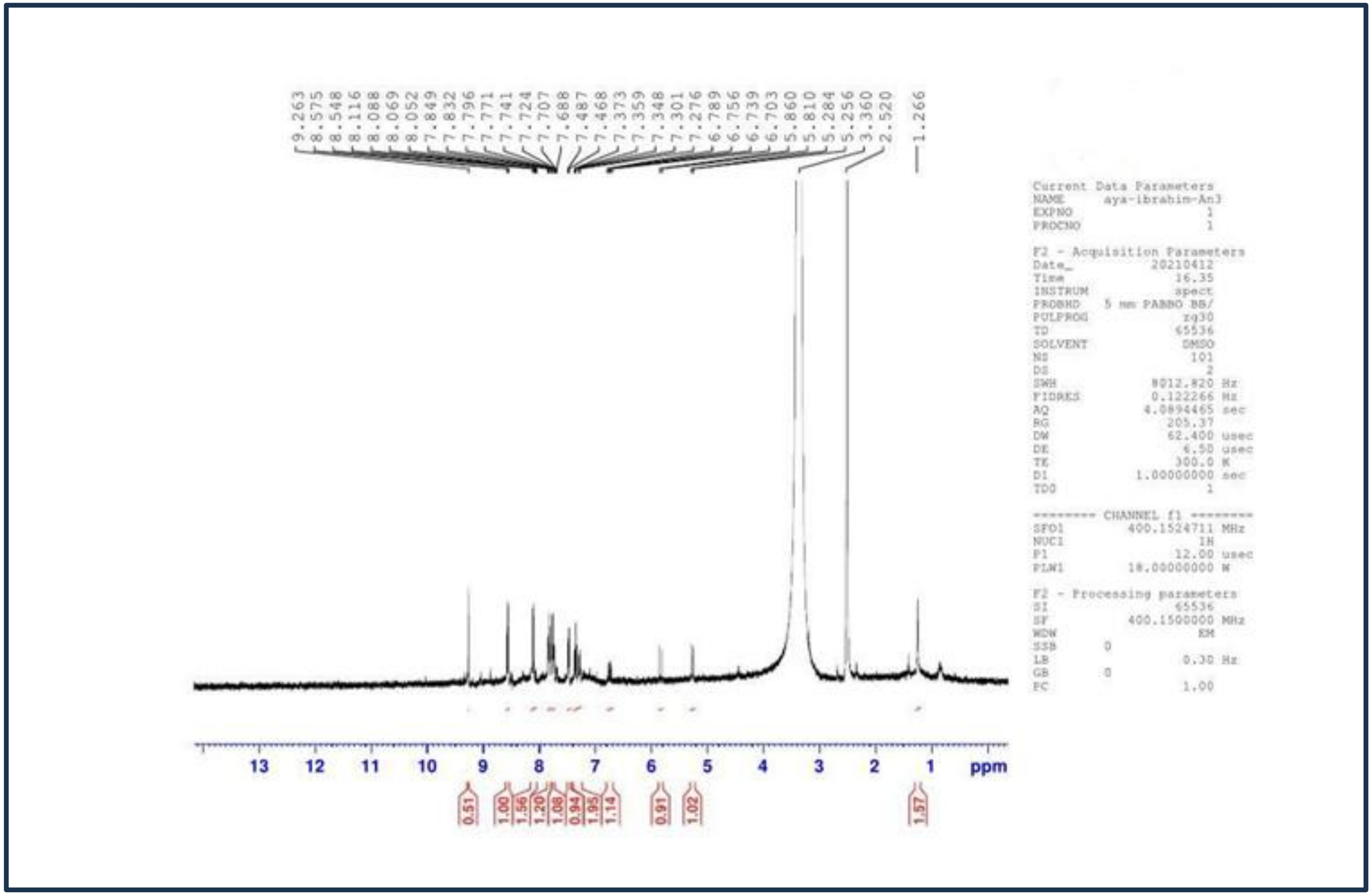
9.263
8.575
8.548
8.116
8.088
8.069
8.052
7.849
7.832
7.796
7.771
7.741
7.724
7.707
7.688
7.487
7.468
7.373
7.359
7.348
7.301
7.276
6.789
6.756
6.739
6.703
5.860
5.810
5.284
5.256
3.360
2.520
1.266
13 12 11 10 9 8 7 6 5 4 3 2 1 ppm
0.51 1.00 1.56 1.20 1.08 0.94 1.95 1.14 0.91 1.02 1.57
Current Data Parameters
NAME aya-ibrahim-An3
EXPNO 1
PROCNO 1
F2 - Acquisition Parameters
Date_ 20210412
Time 16.35
INSTRUM spect
PROBHD 5 mm PABBO BB/
PULPROG zg30
TD 65536
SOLVENT DMSO
NS 101
DS 2
SWH 8012.820 Hz
FIDRES 0.122266 Hz
AQ 4.0894465 sec
RG 205.37
DW 62.400 usec
DE 6.50 usec
TE 300.0 K
D1 1.00000000 sec
TD0 1
======== CHANNEL f1 ========
SFO1 400.1524711 MHz
NUC1 1H
P1 12.00 usec
PLW1 18.00000000 W
F2 - Processing parameters
SI 65536
SF 400.1500000 MHz
WDW EM
SSB 0
LB 0.30 Hz
GB 0
PC 1.00

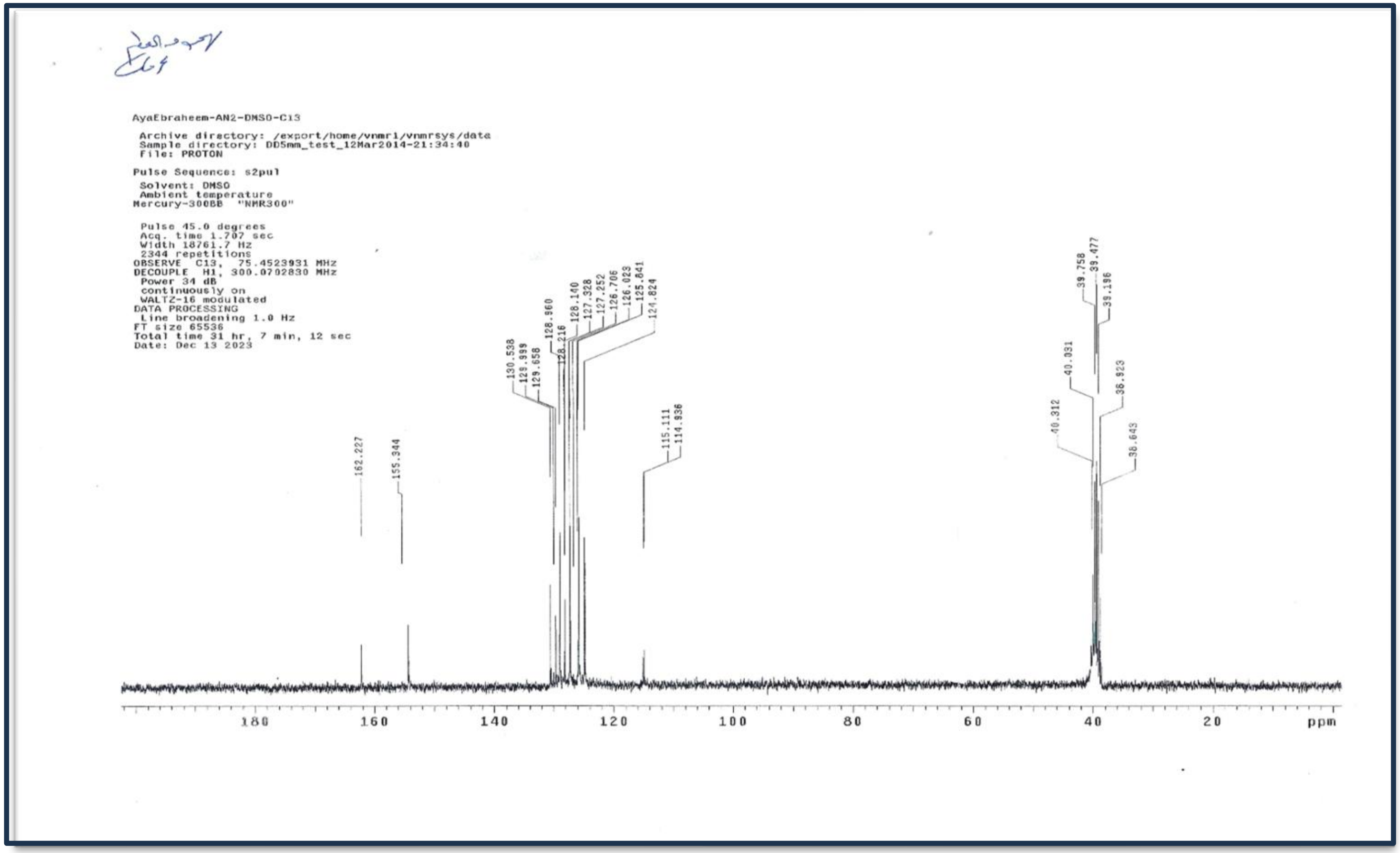

Figure S3. ^{13}C NMR (300 MHz, DMSO-d_6) spectrum of compound **4**

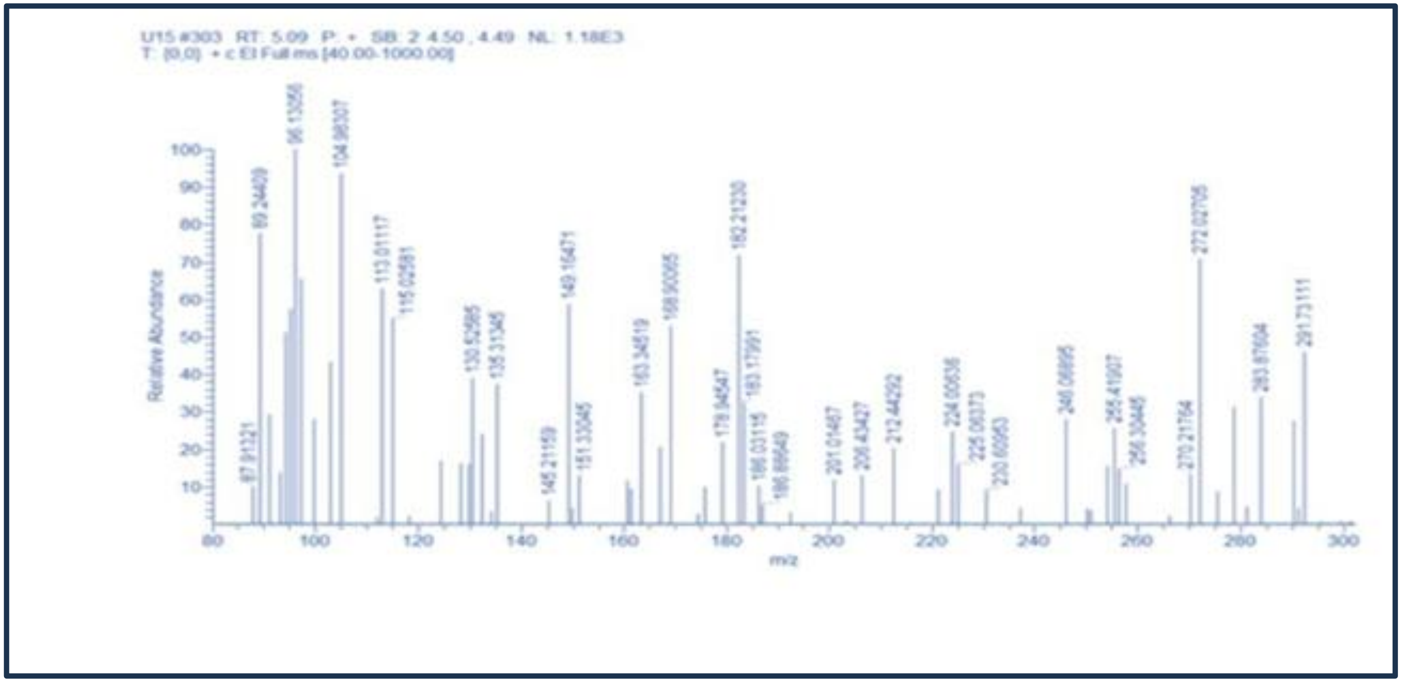

Figure S4. HR-Mass spectrum of compound **4**

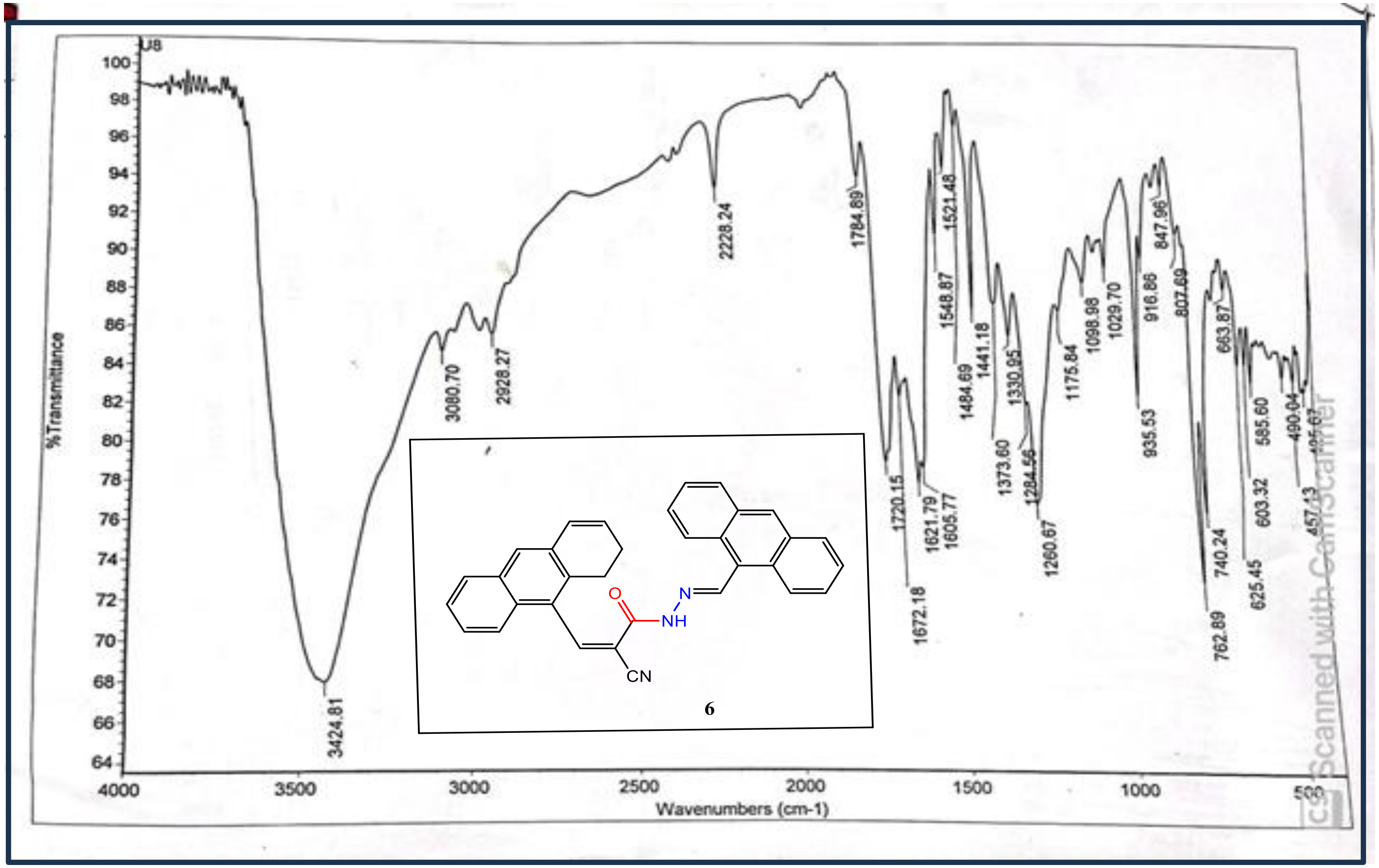

Figure S5. IR spectrum of compound **6**

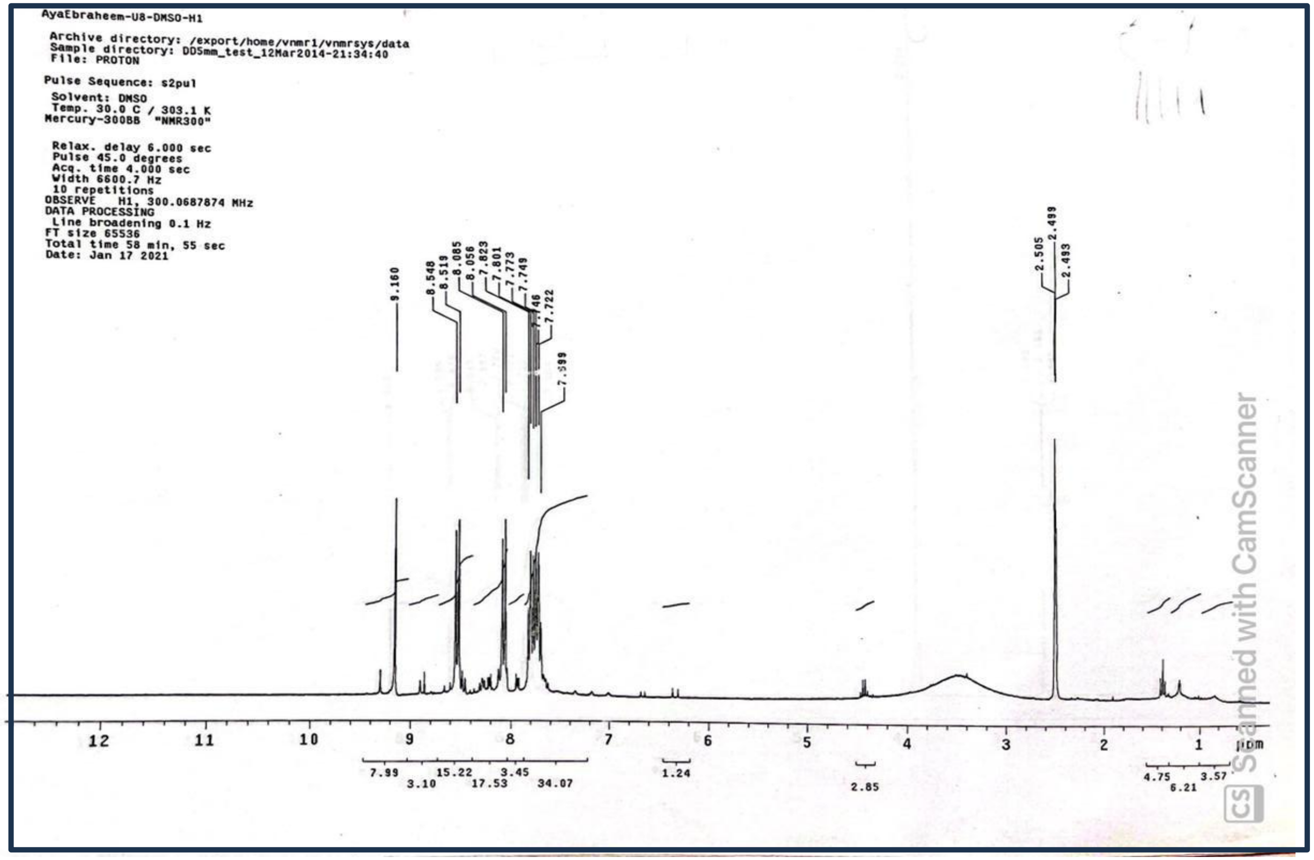

Figure S6. ^{1}H NMR (300 MHz, DMSO-d_6) spectrum of compound **6**

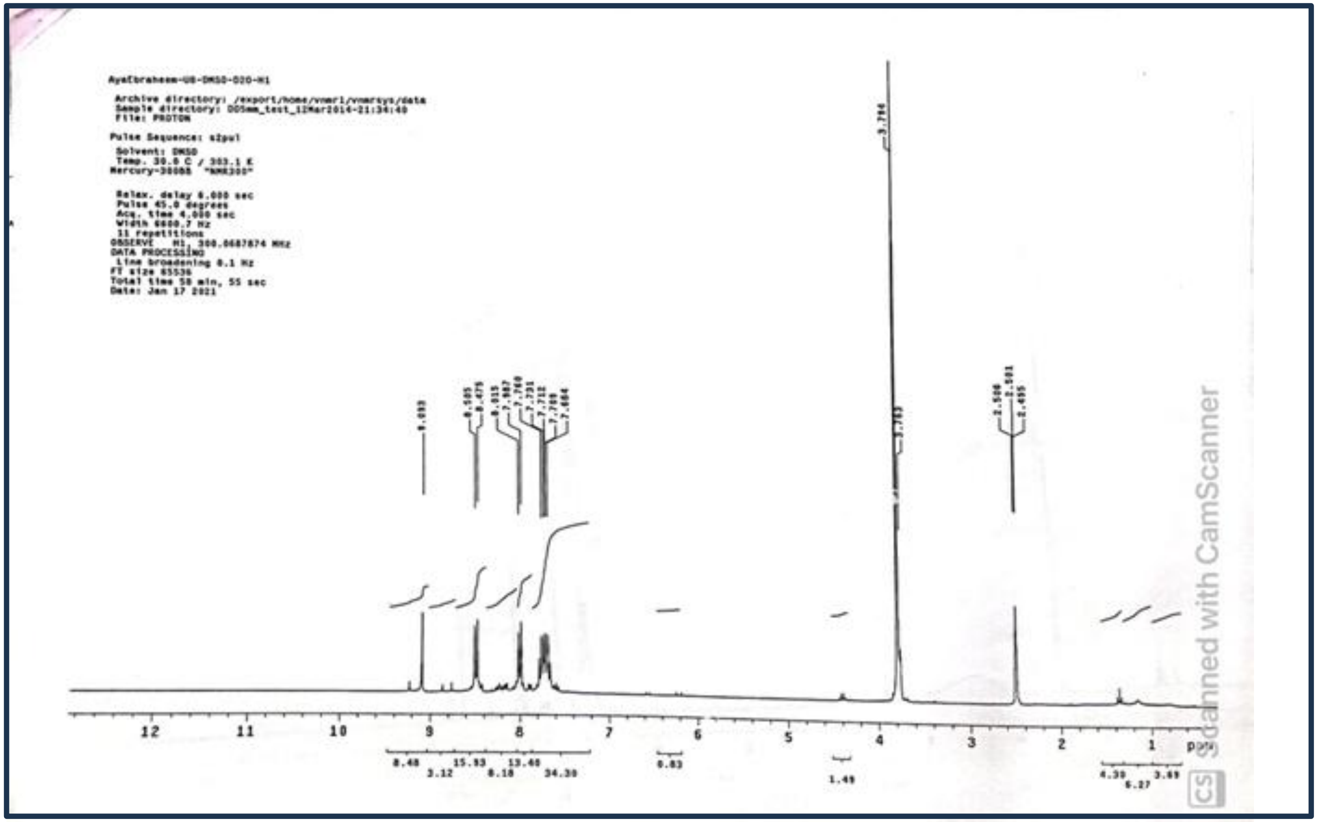

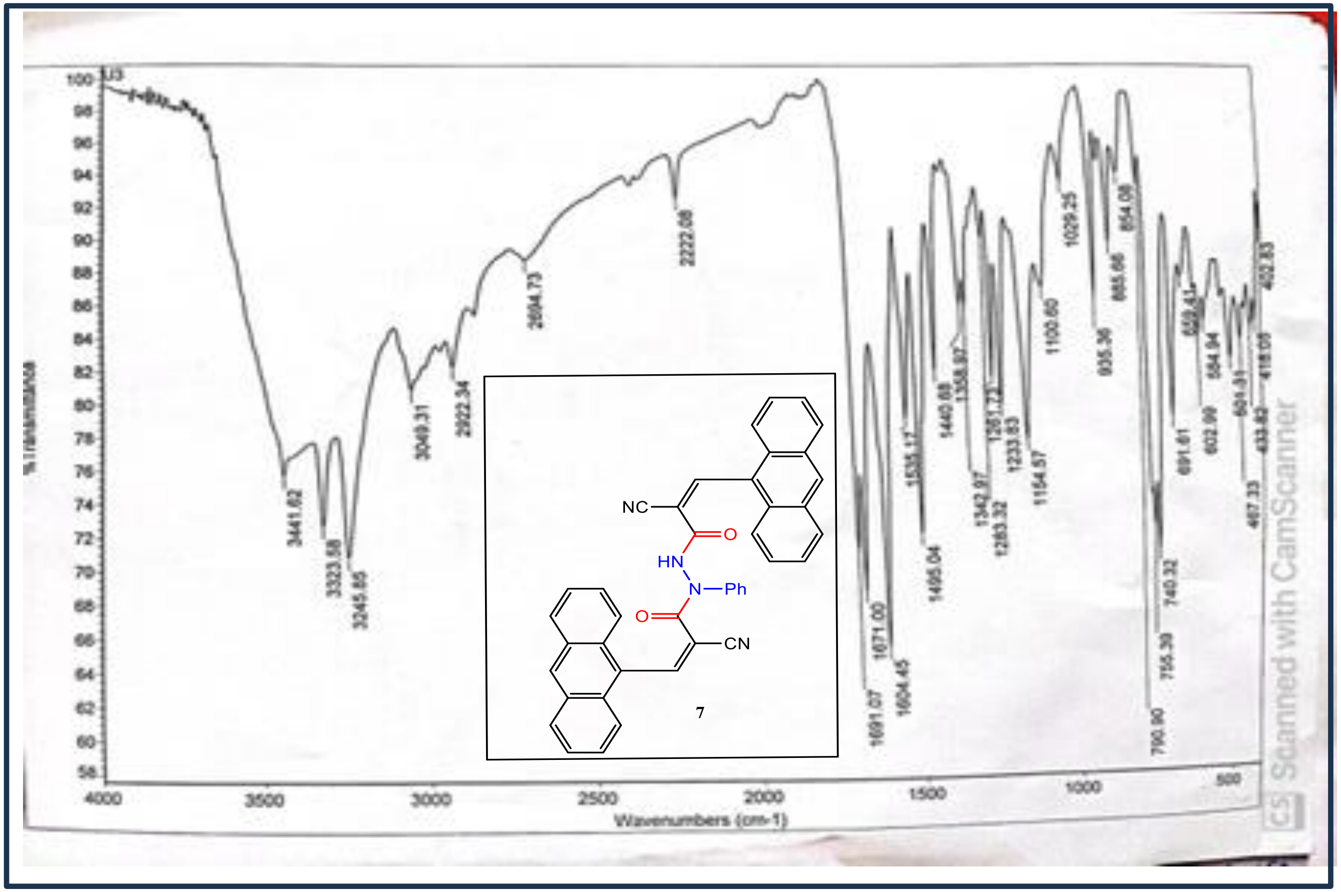

Figure S7. IR spectrum of compound **7**

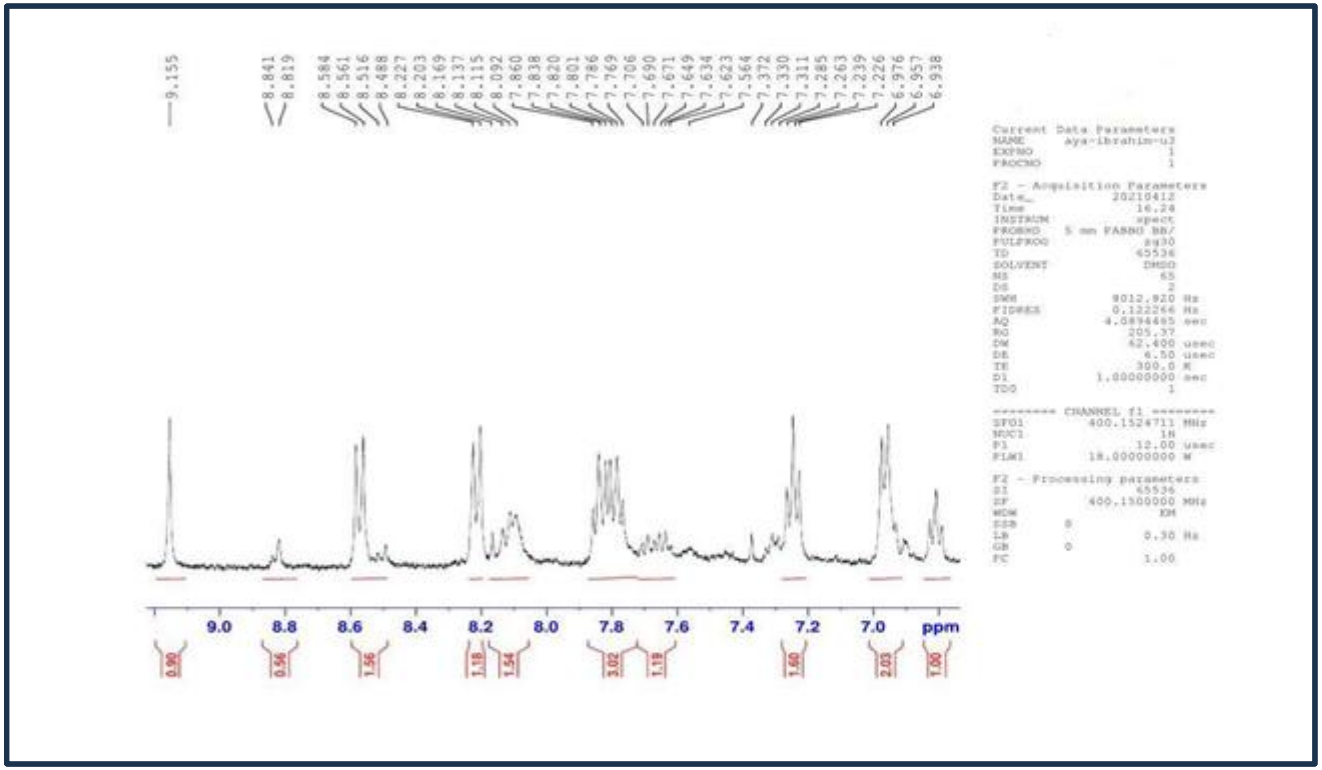

Figure S8. ^{1}H NMR (300 MHz, DMSO-d_6) spectrum of compound **7**

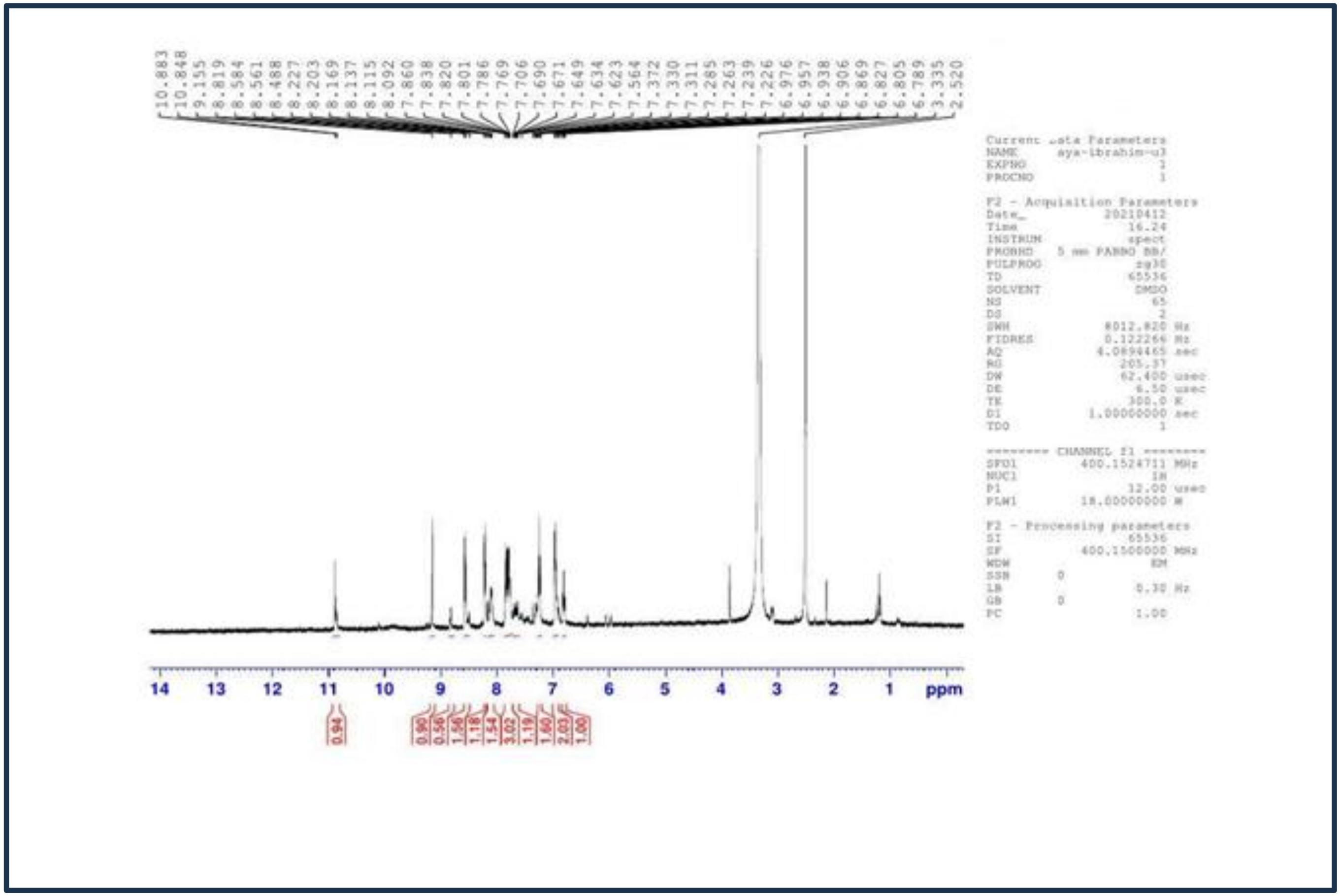

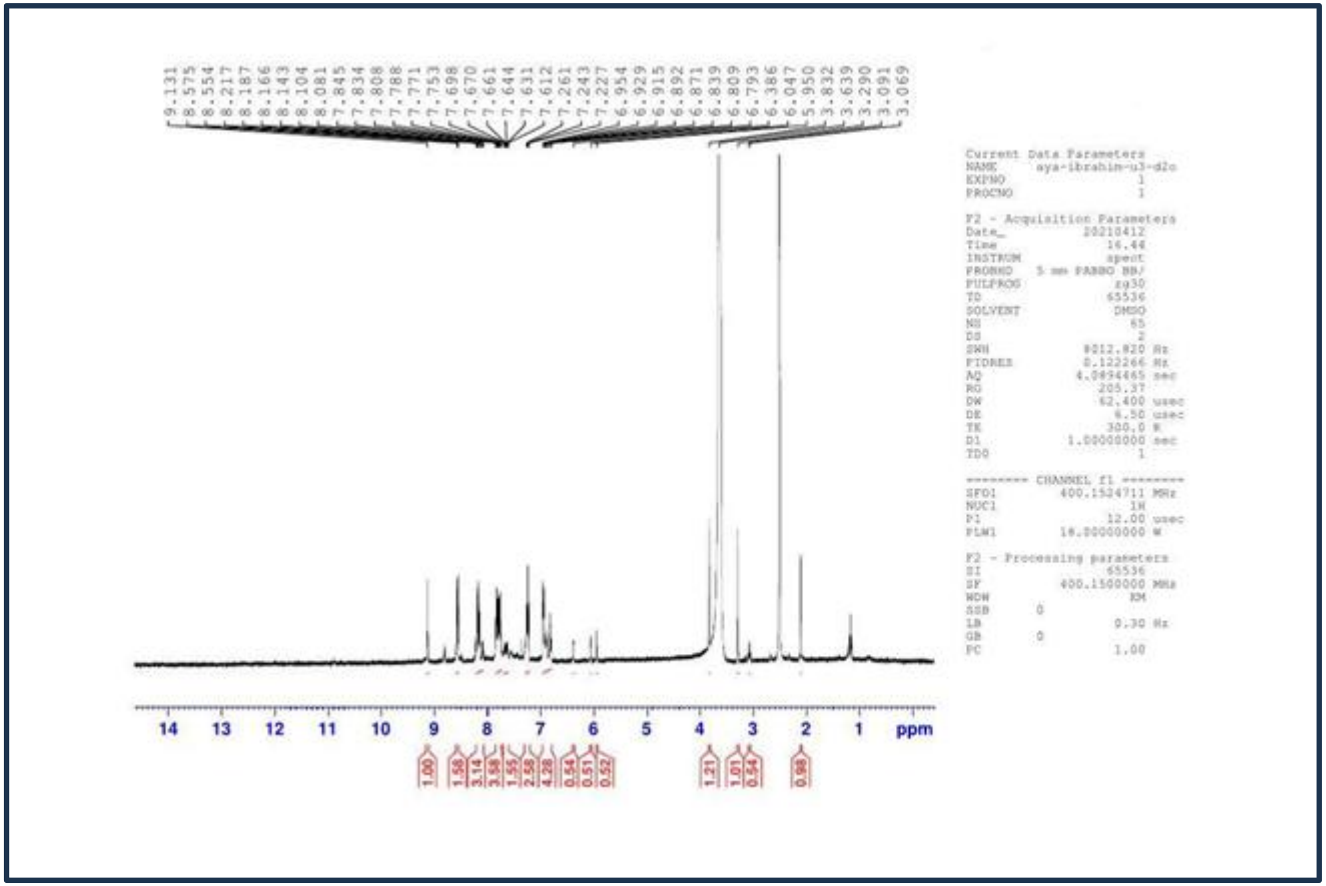

9.131 8.575 8.554 8.217 8.187 8.166 8.143 8.104 8.081 7.845 7.834 7.808 7.788 7.771 7.753 7.698 7.670 7.661 7.644 7.631 7.612 7.261 7.243 7.227 6.954 6.929 6.915 6.892 6.871 6.839 6.809 6.793 6.386 6.047 5.950 3.832 3.639 3.290 3.091 3.069
Current Data Parameters
NAME aya-ibrahim-u3-d2o
EXPNO 1
PROCNO 1
F2 - Acquisition Parameters
Date_ 20210412
Time 16.44
INSTRUM spect
PROBHD 5 mm PABBO BB/
PULPROG zg30
TD 65536
SOLVENT DMSO
NS 65
DS 2
SWH 8012.820 Hz
FIDRES 0.122266 Hz
AQ 4.0894465 sec
RG 205.37
DW 62.400 usec
DE 6.50 usec
TE 300.0 K
D1 1.00000000 sec
TD0 1
======== CHANNEL f1 ========
SFO1 400.1524711 MHz
NUC1 1H
P1 12.00 usec
PLW1 18.00000000 W
F2 - Processing parameters
SI 65536
SF 400.1500000 MHz
WDW EM
SSB 0
LB 0.30 Hz
GB 0
PC 1.00
14 13 12 11 10 9 8 7 6 5 4 3 2 1 ppm
1.00 1.58 3.14 3.58 1.55 2.58 4.28 0.54 0.51 0.52 1.21 1.01 0.54 0.98

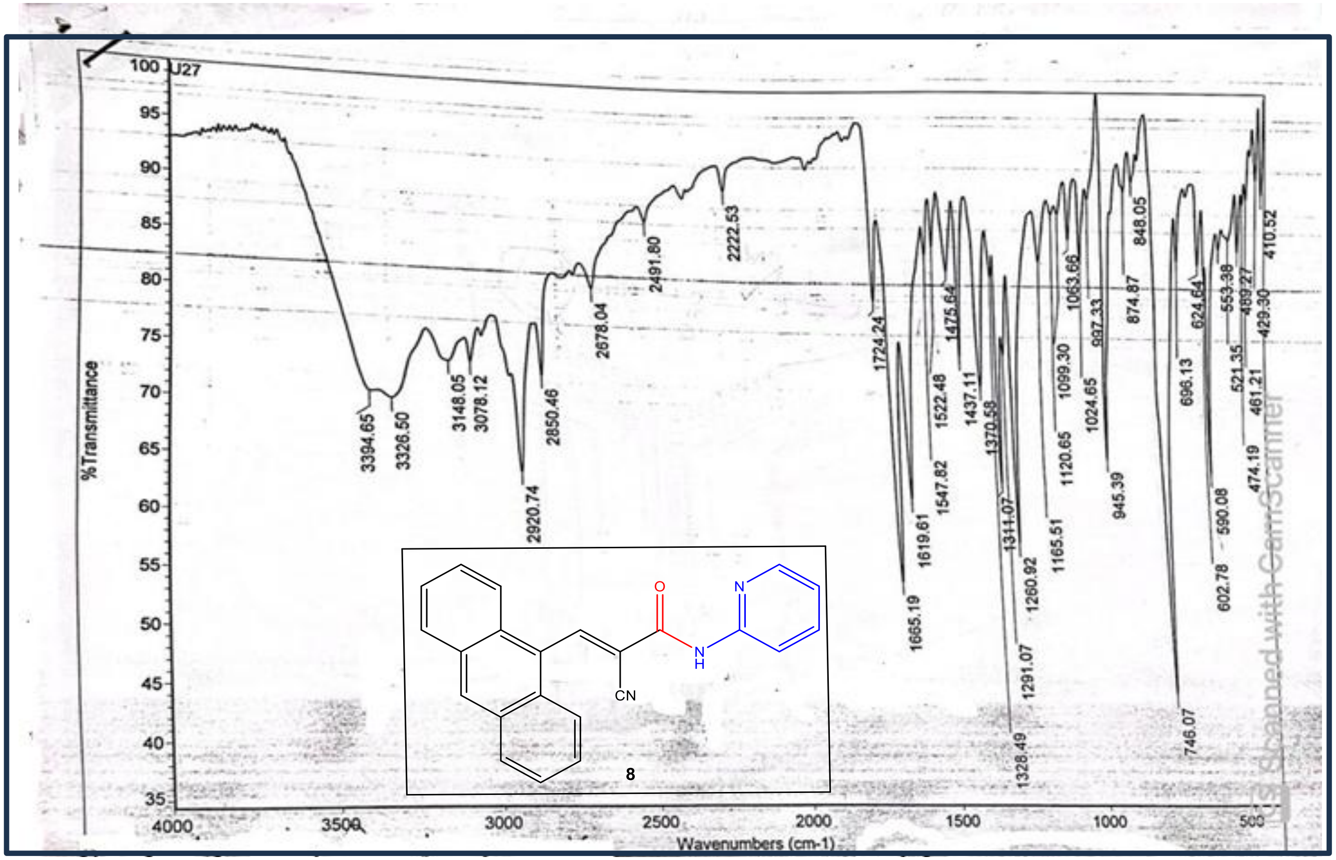

Figure S9. IR spectrum of compound **8**

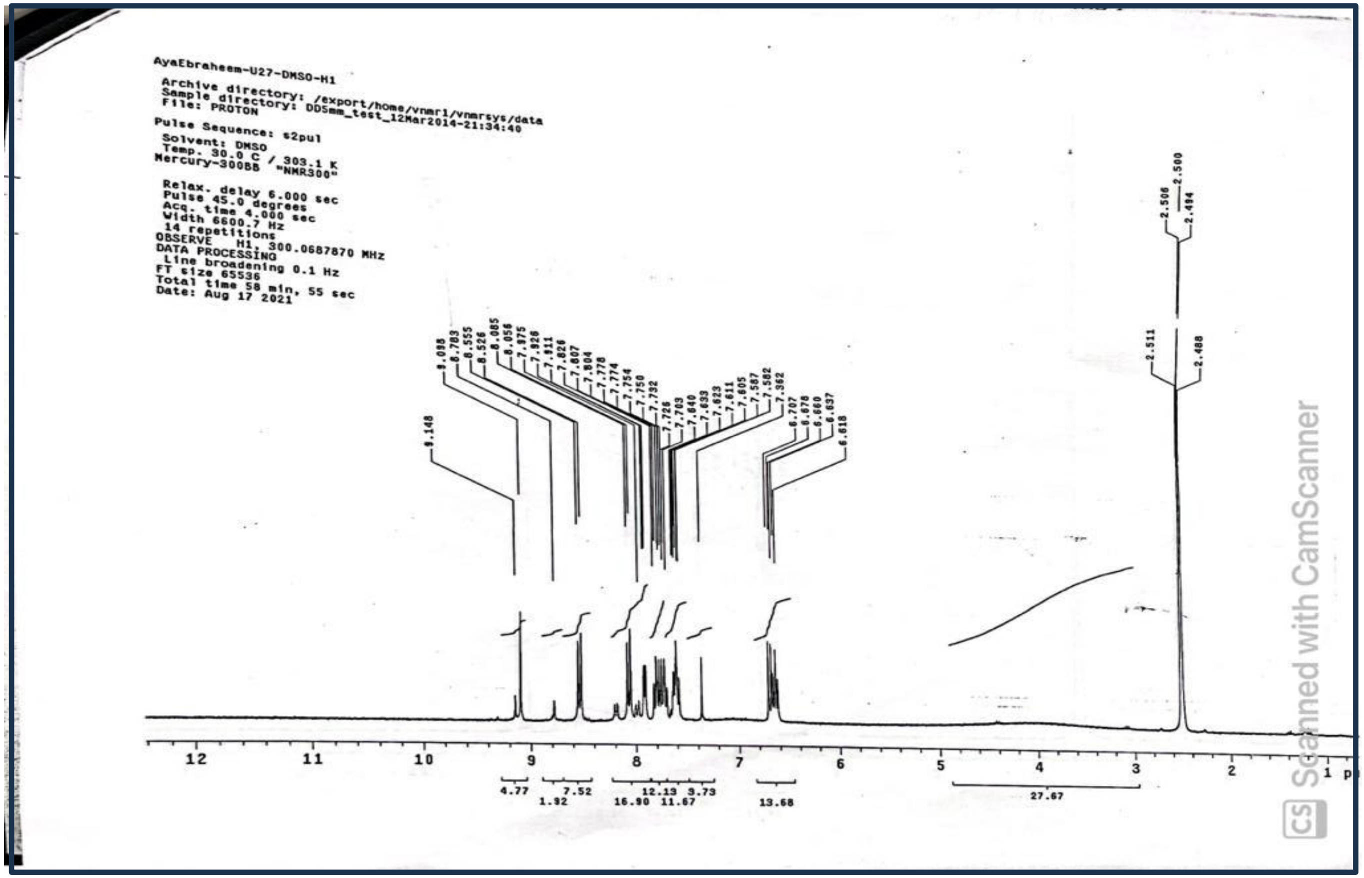

Figure S10. ^{1}H NMR (300 MHz, DMSO-d_6) spectrum of compound **8**

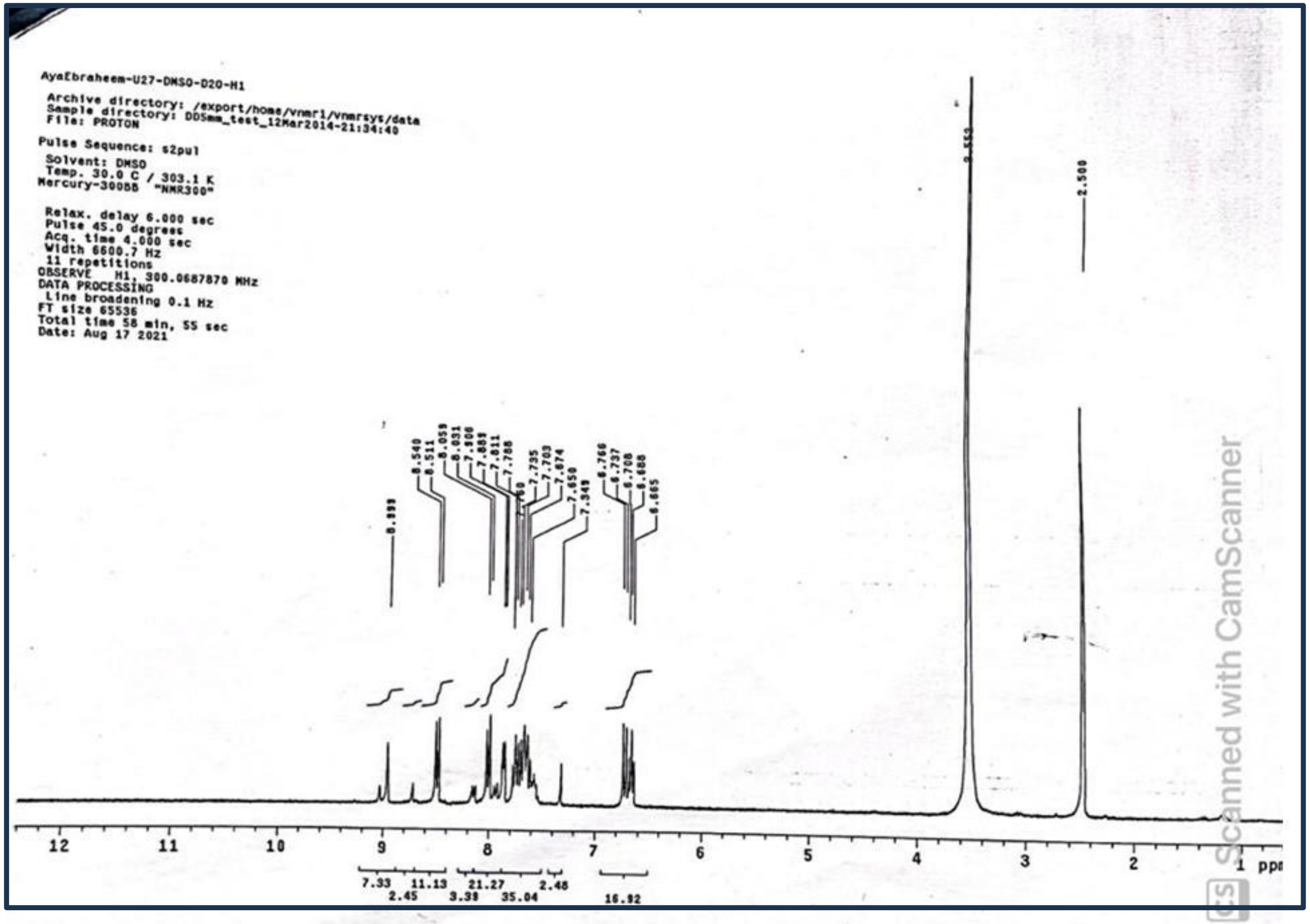
AyaEbraheem-U27-DMSO-D2O-H1
Archive directory: /export/home/vnmr1/vnmrsys/data
Sample directory: DD5mm_test_12Mar2014-21:34:40
File: PROTON
Pulse Sequence: s2pul
Solvent: DMSO
Temp. 30.0 C / 303.1 K
Mercury-300BB "NMR300"
Relax. delay 6.000 sec
Pulse 45.0 degrees
Acq. time 4.000 sec
Width 6600.7 Hz
11 repetitions
OBSERVE H1, 300.0687870 MHz
DATA PROCESSING
Line broadening 0.1 Hz
FT size 65536
Total time 58 min, 55 sec
Date: Aug 17 2021
ppm

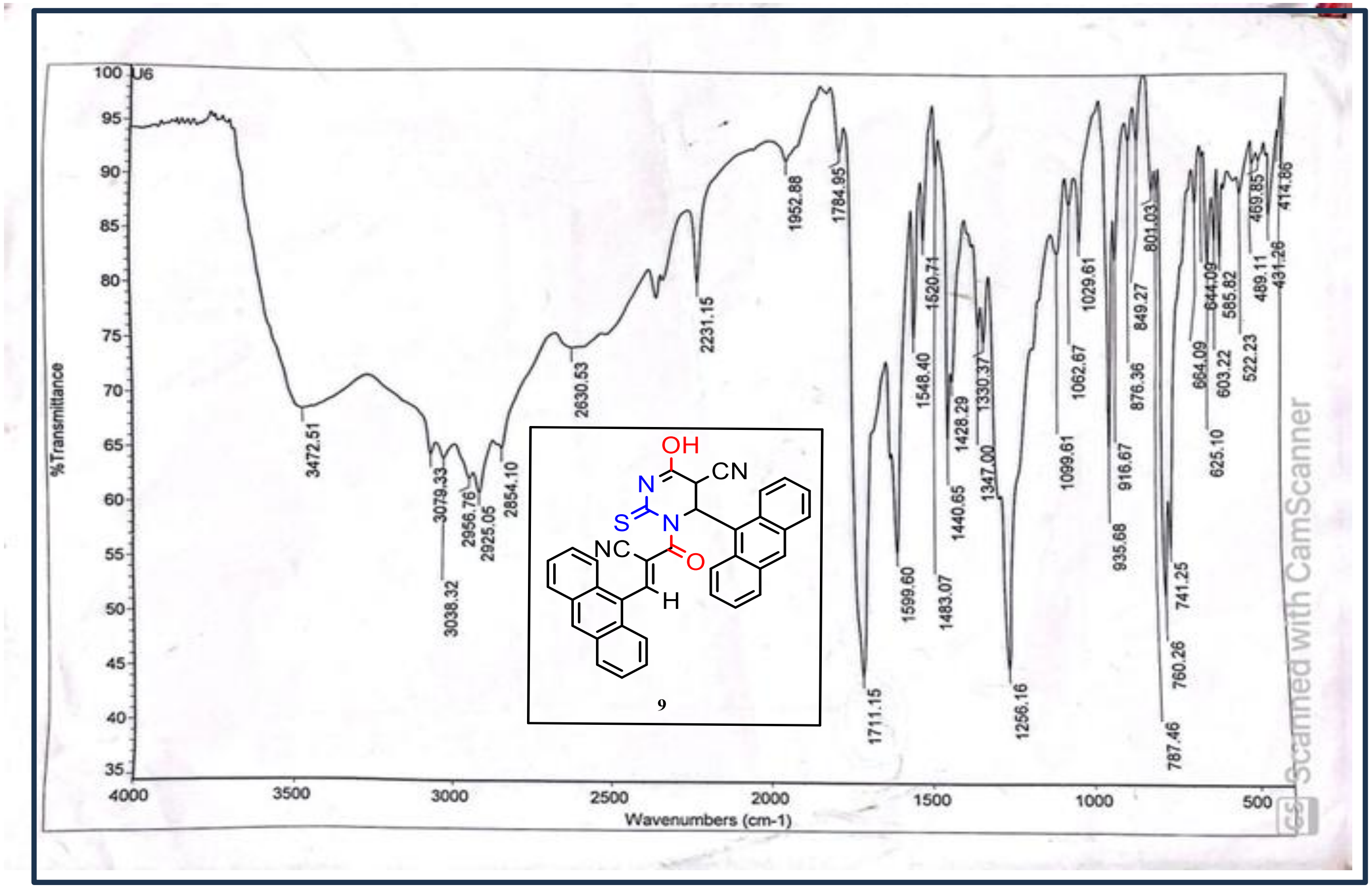

Figure S11. IR spectrum of compound **9**

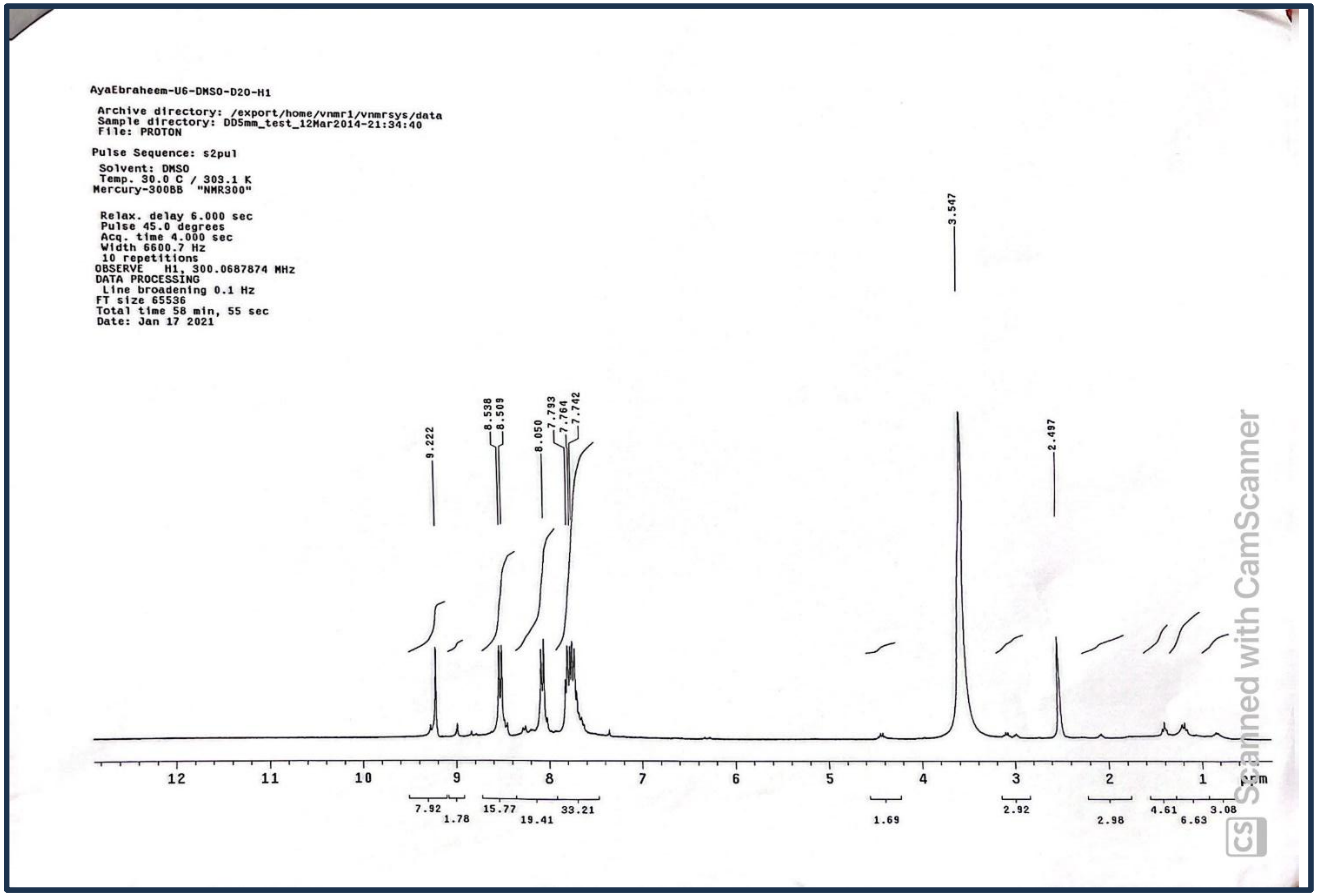

Figure S12. ^{1}H NMR (300 MHz, DMSO-d_6) spectrum of compound 9

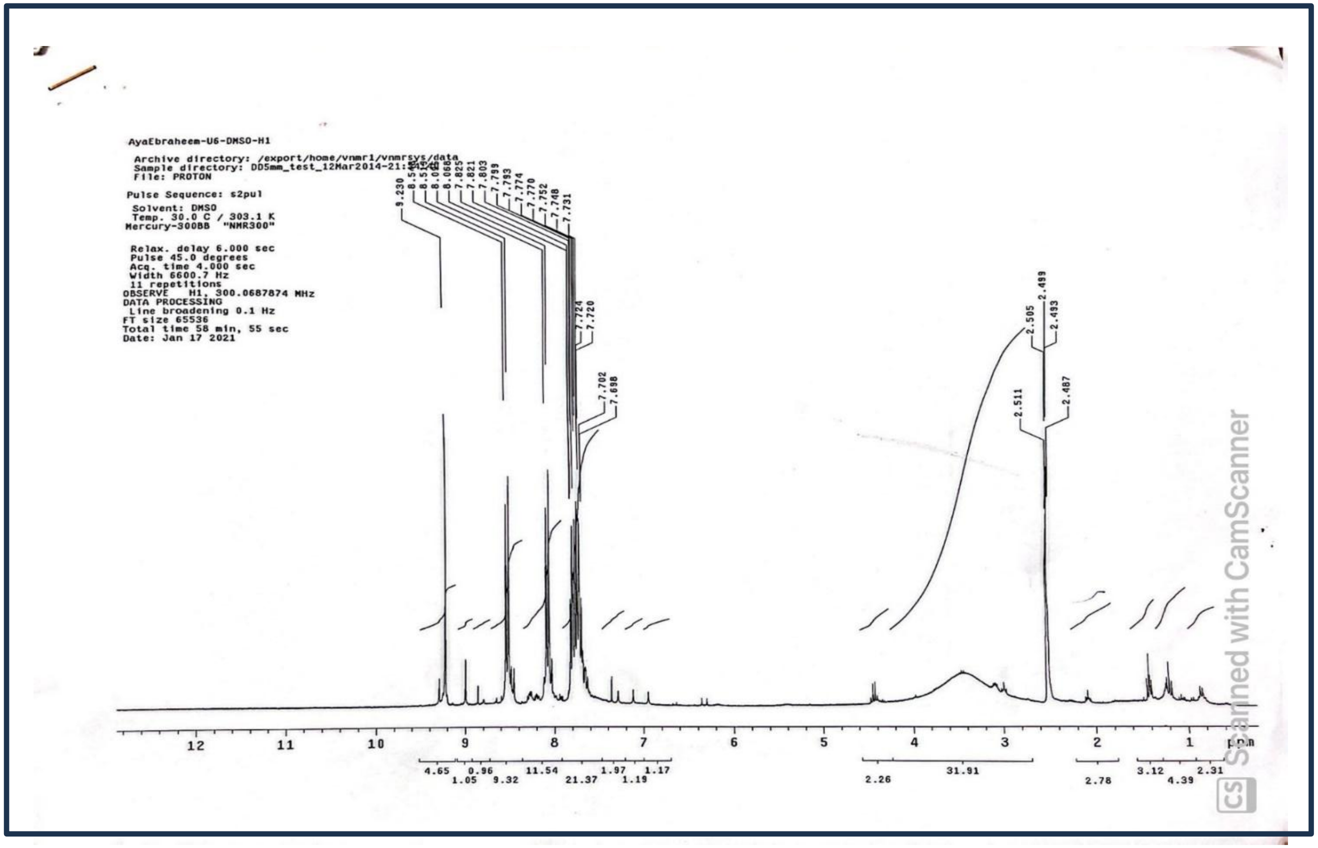

Scanned with CamScanner

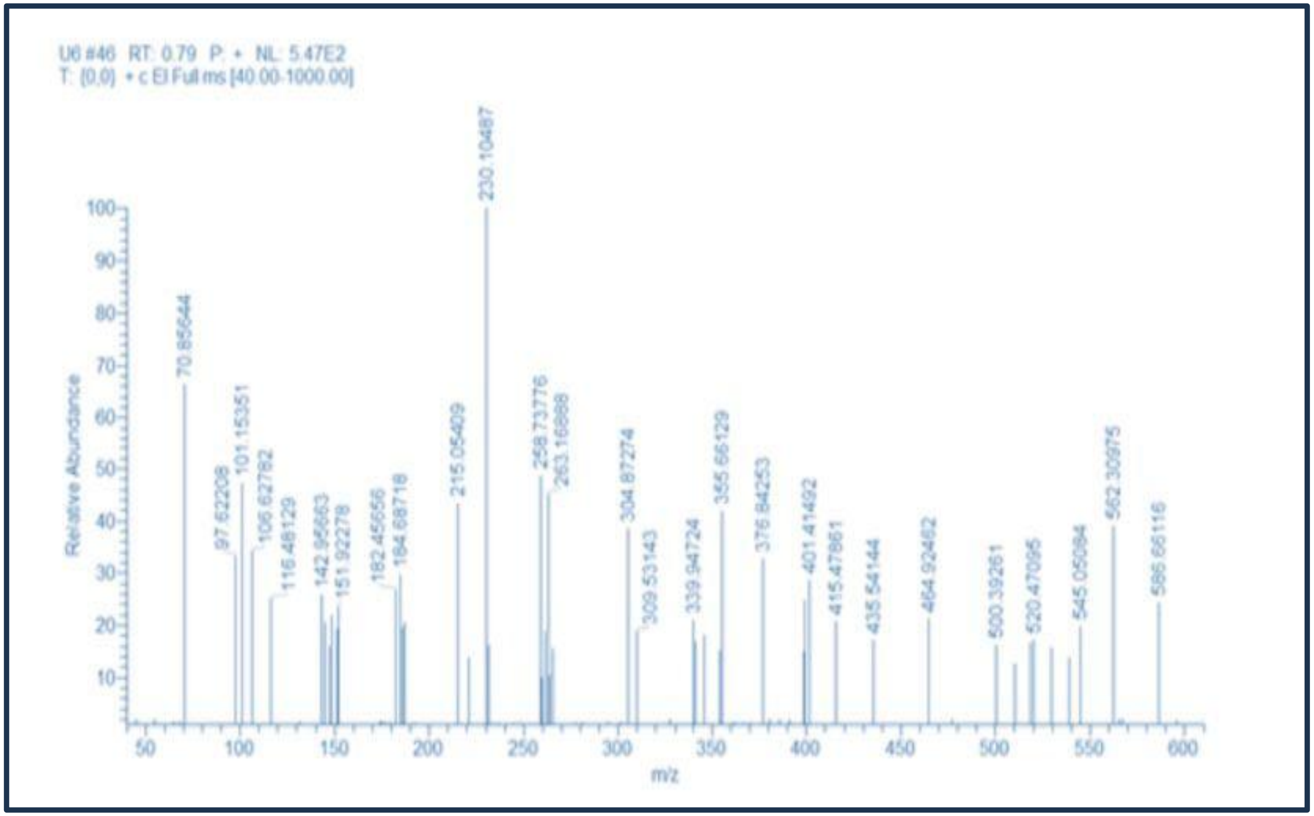

Figure S13: HR-Mass spectrum of compound **9**

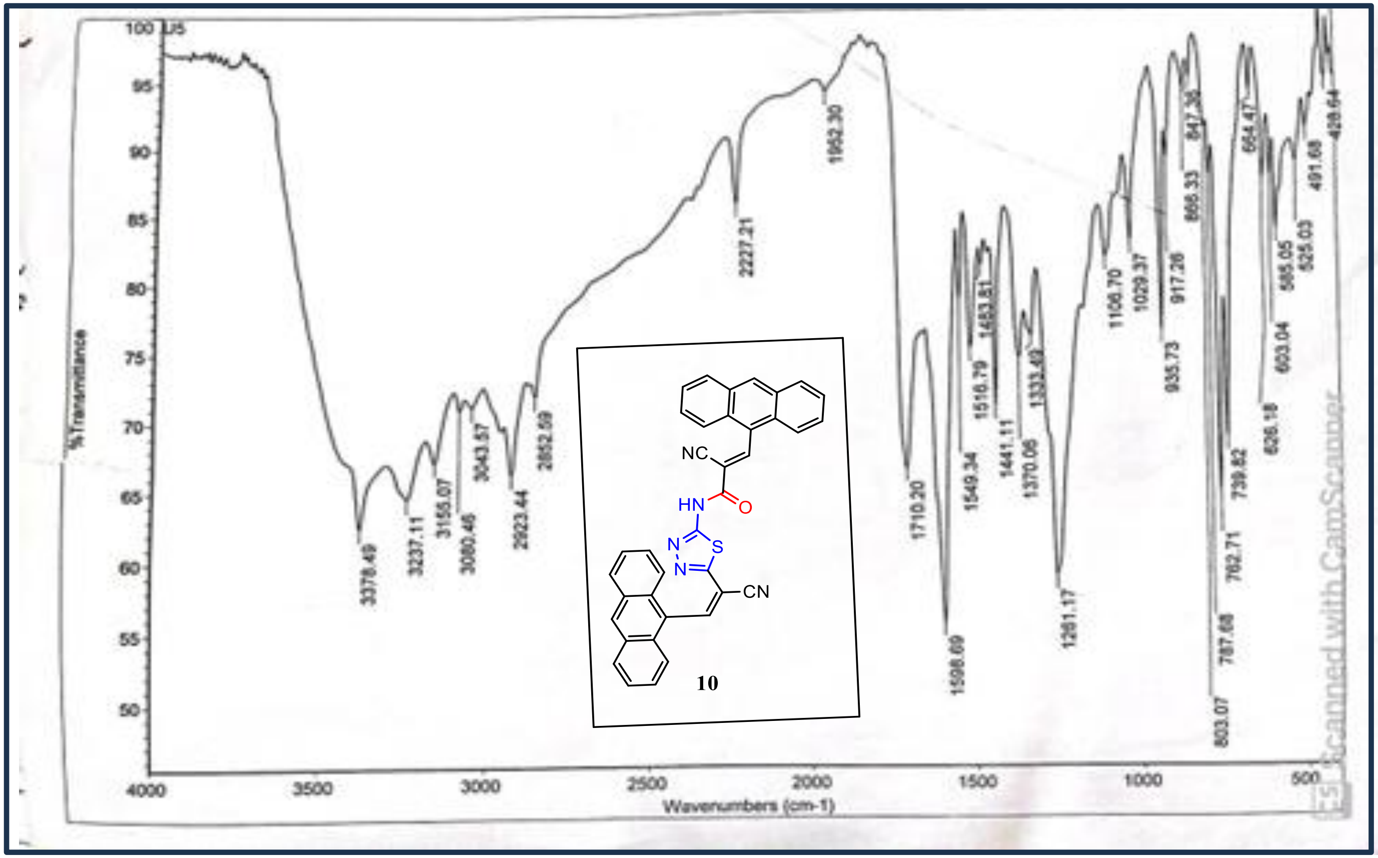

Figure S14. IR spectrum of compound **10**

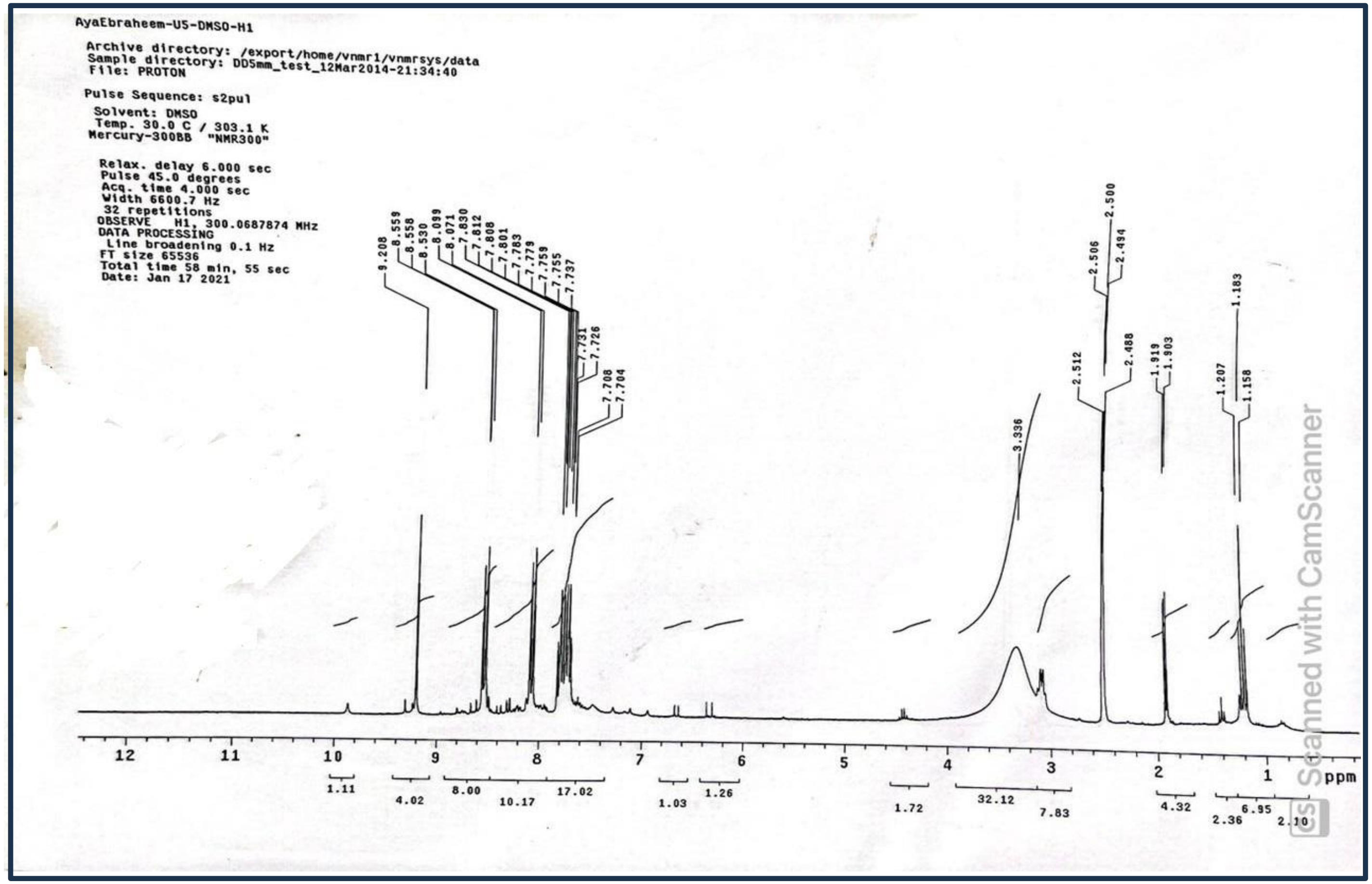

Figure S15. ^{1}H NMR (300 MHz, DMSO-d_6) spectrum of compound **10**

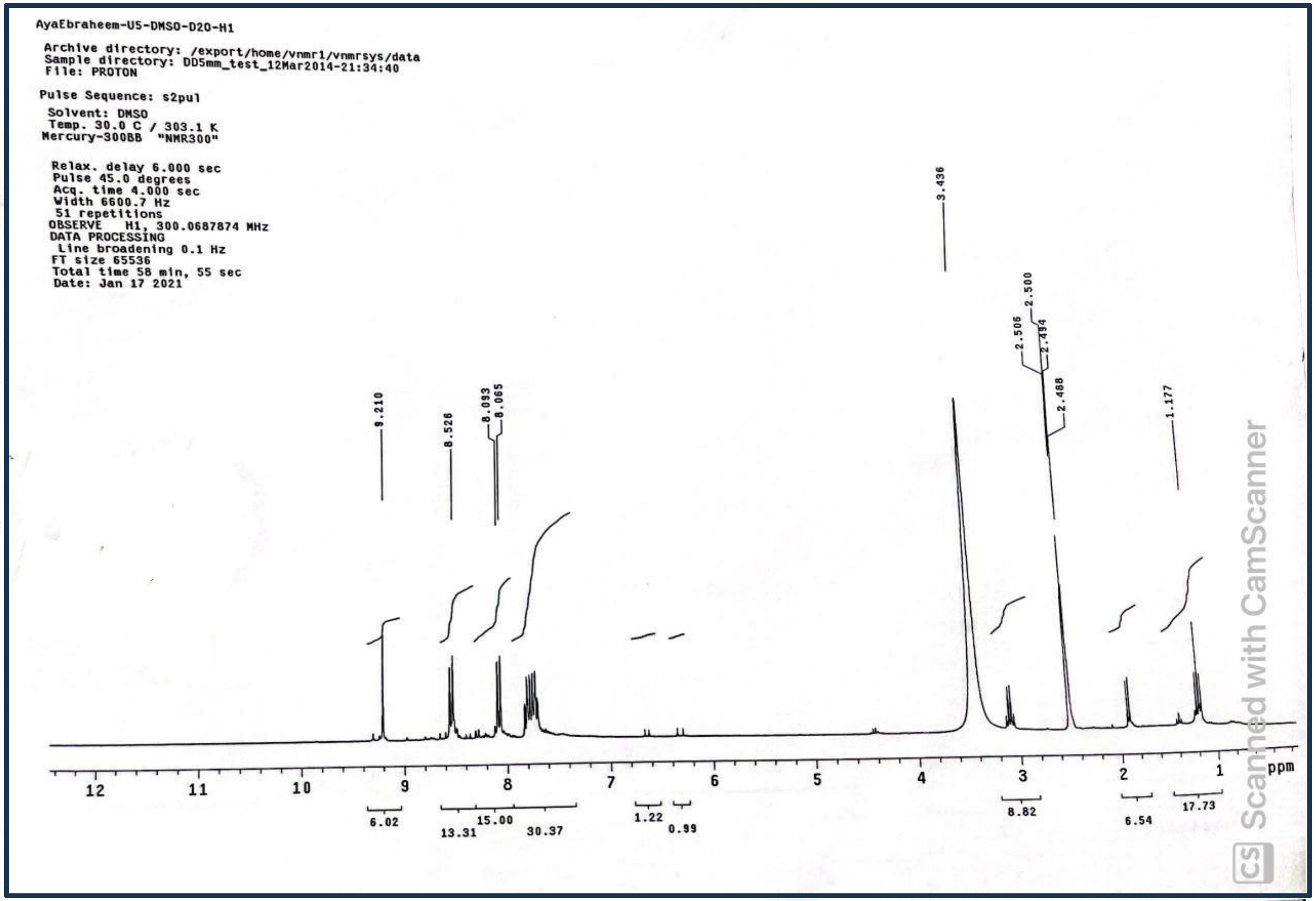
AyaEbraheem-U5-DMSO-D2O-H1
Archive directory: /export/home/vnmr1/vnmrsys/data
Sample directory: DD5mm_test_12Mar2014-21:34:40
File: PROTON
Pulse Sequence: s2pul
Solvent: DMSO
Temp. 30.0 C / 303.1 K
Mercury-300BB "NMR300"
Relax. delay 6.000 sec
Pulse 45.0 degrees
Acq. time 4.000 sec
Width 6600.7 Hz
51 repetitions
OBSERVE H1, 300.0687874 MHz
DATA PROCESSING
Line broadening 0.1 Hz
FT size 65536
Total time 58 min, 55 sec
Date: Jan 17 2021
9.210
8.526
8.093
8.065
3.436
2.506
2.500
2.494
2.488
1.177
12 11 10 9 8 7 6 5 4 3 2 1 ppm
6.02
13.31
15.00
30.37
1.22
0.99
8.82
6.54
17.73
Scanned with CamScanner

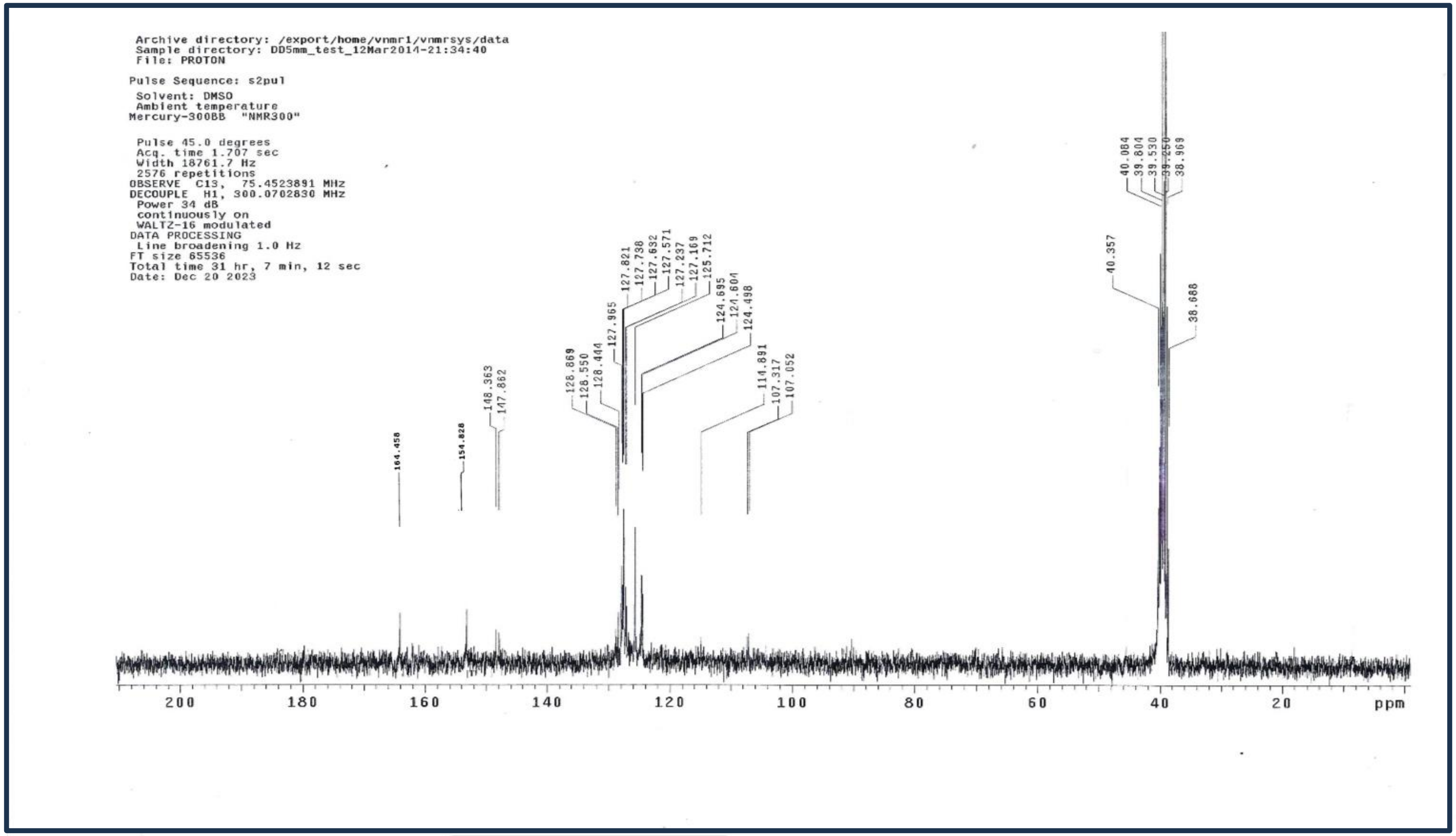

Figure S16. ^{13}C NMR (300 MHz, DMSO-d_6) spectrum of compound **10**

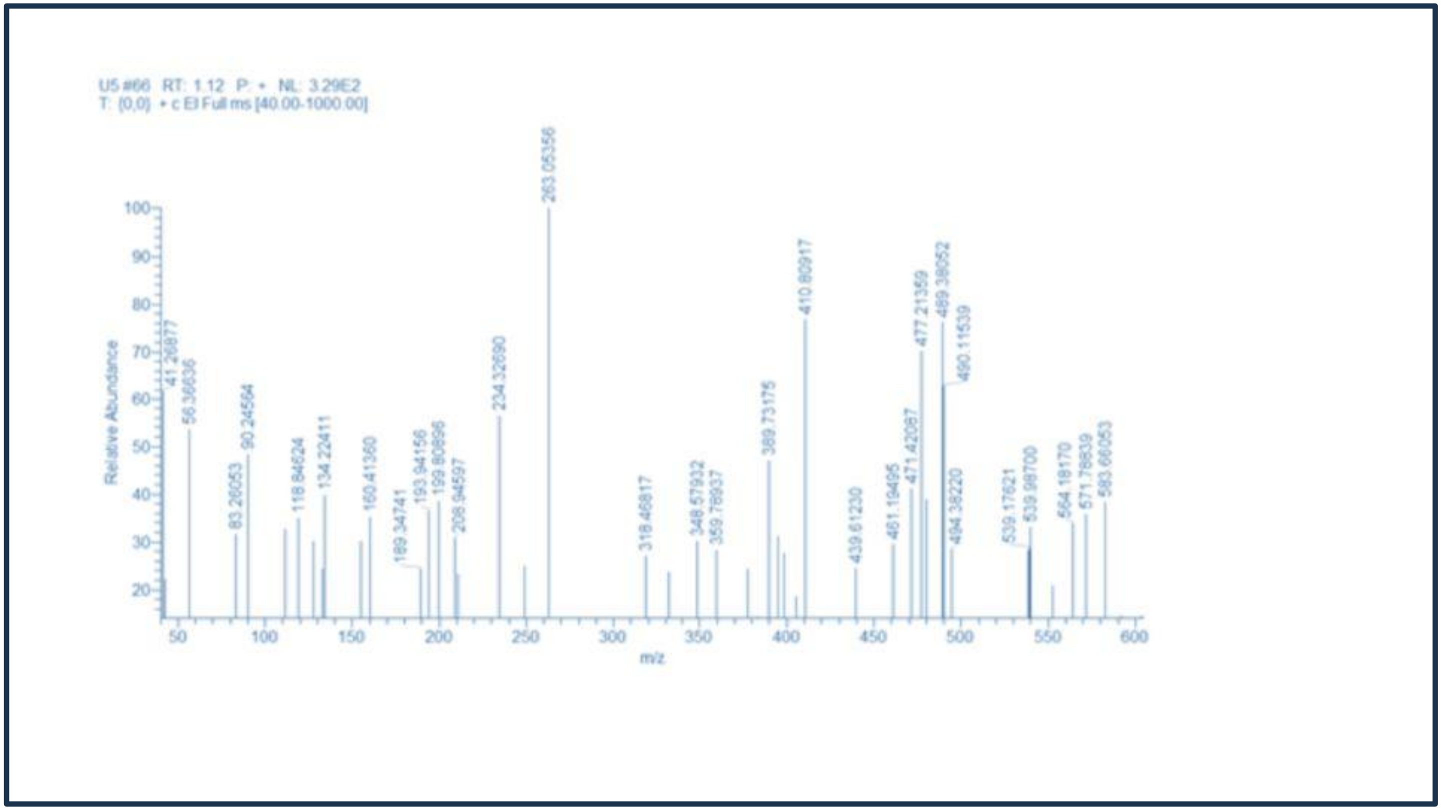

Figure S17. HR-Mass spectrum of compound **10**

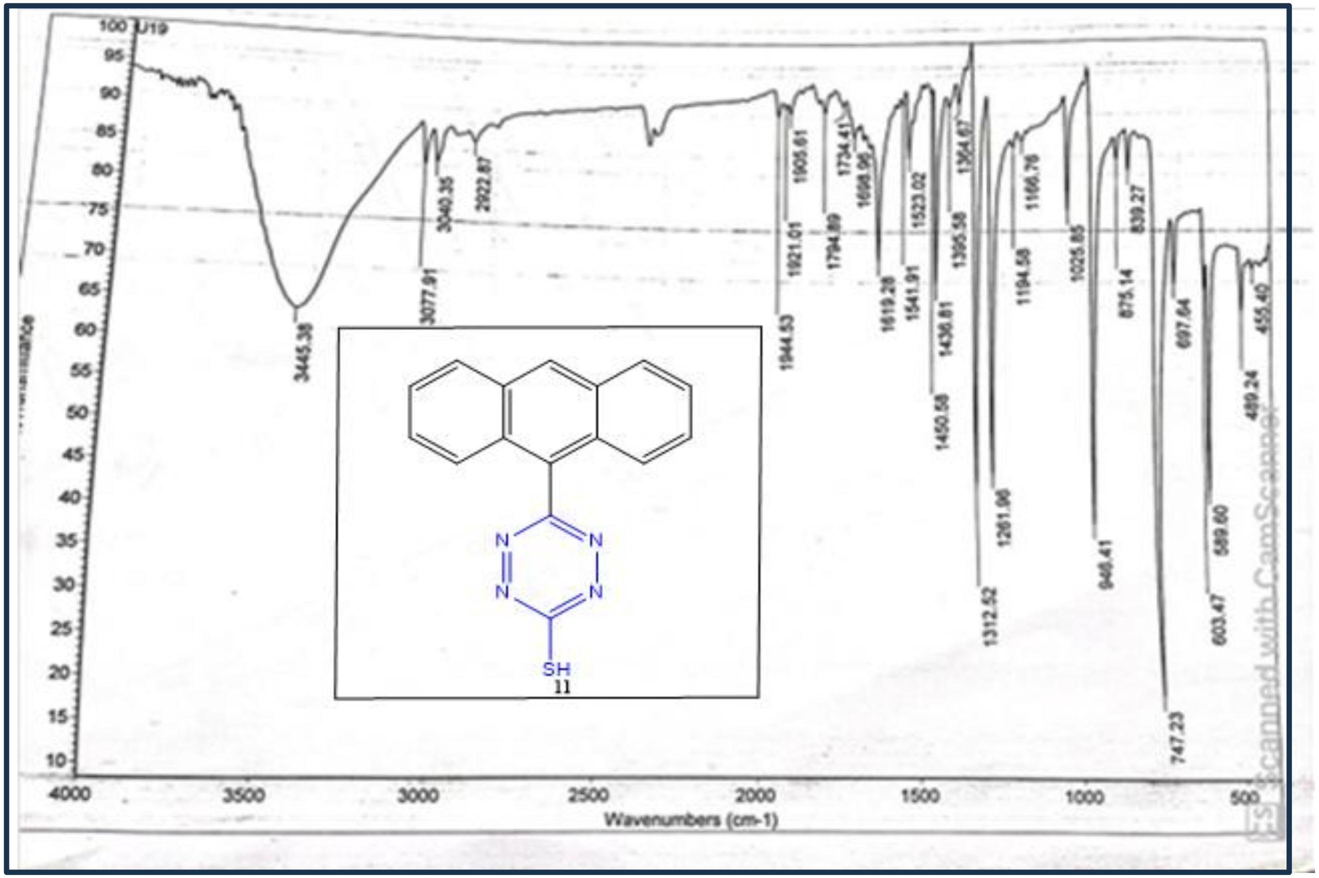

Figure S18. IR spectrum of compound **11**

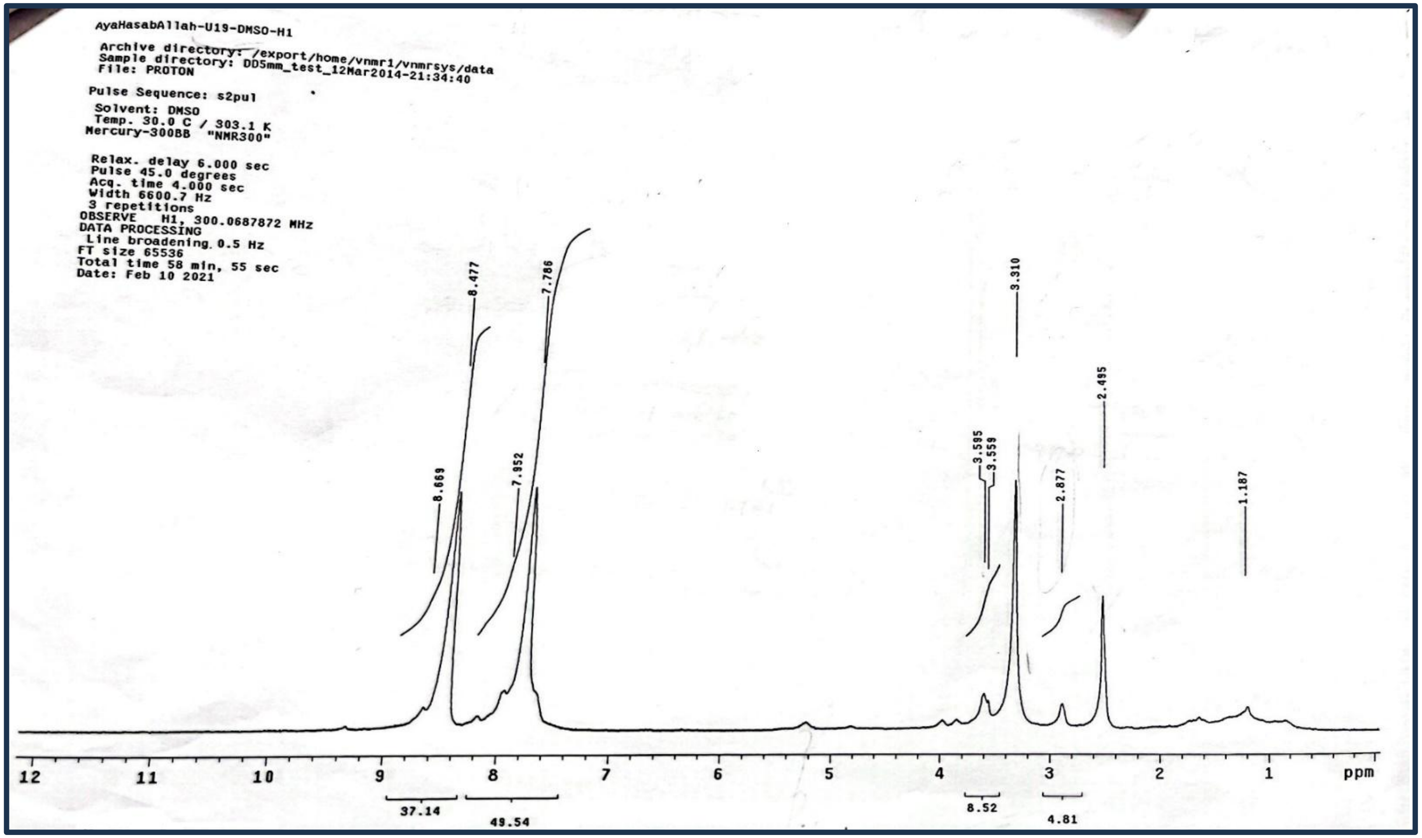

Figure S19. ^{1}H NMR (300 MHz, DMSO-d_6) spectrum of compound **11**

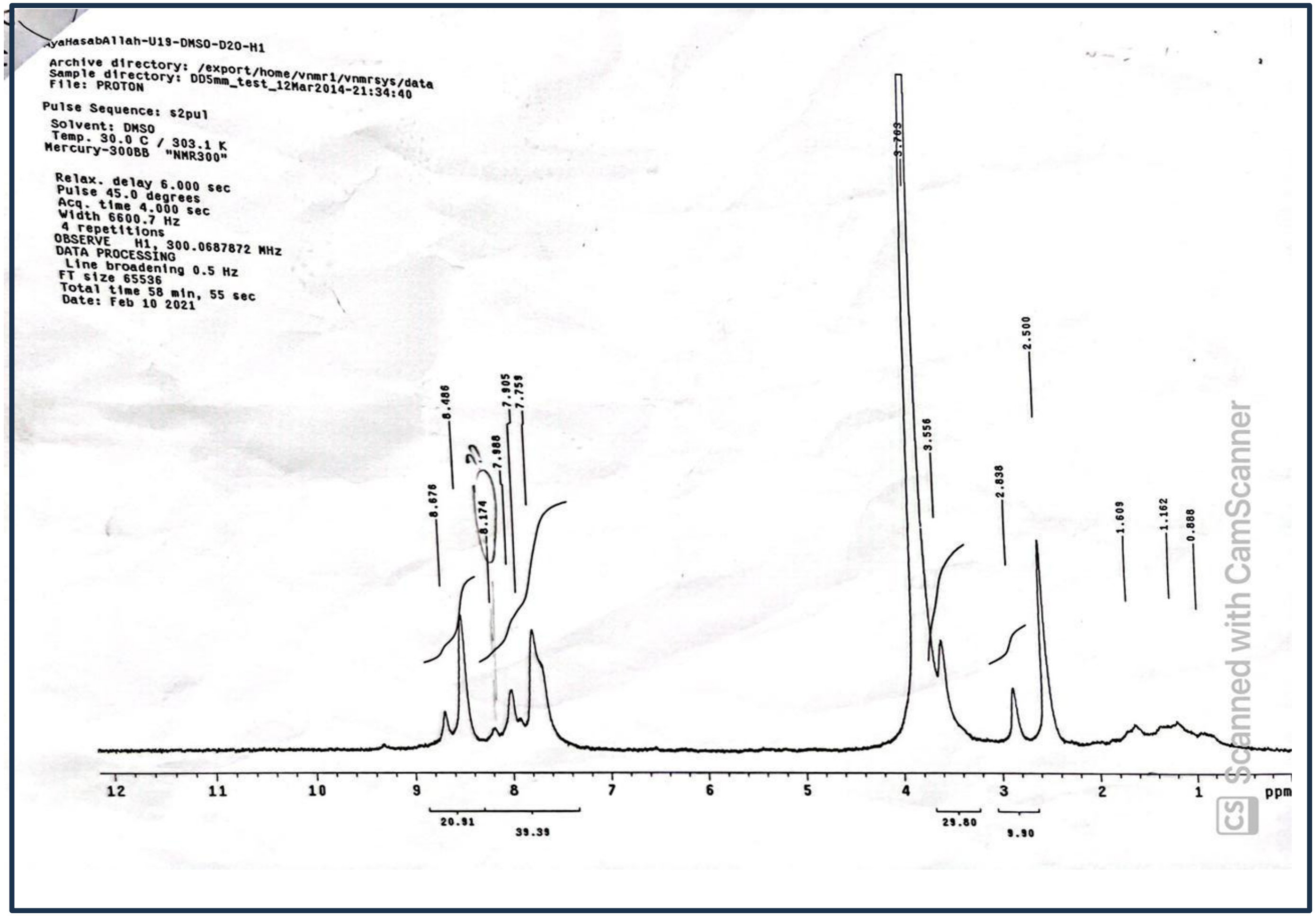

Scanned with CamScanner

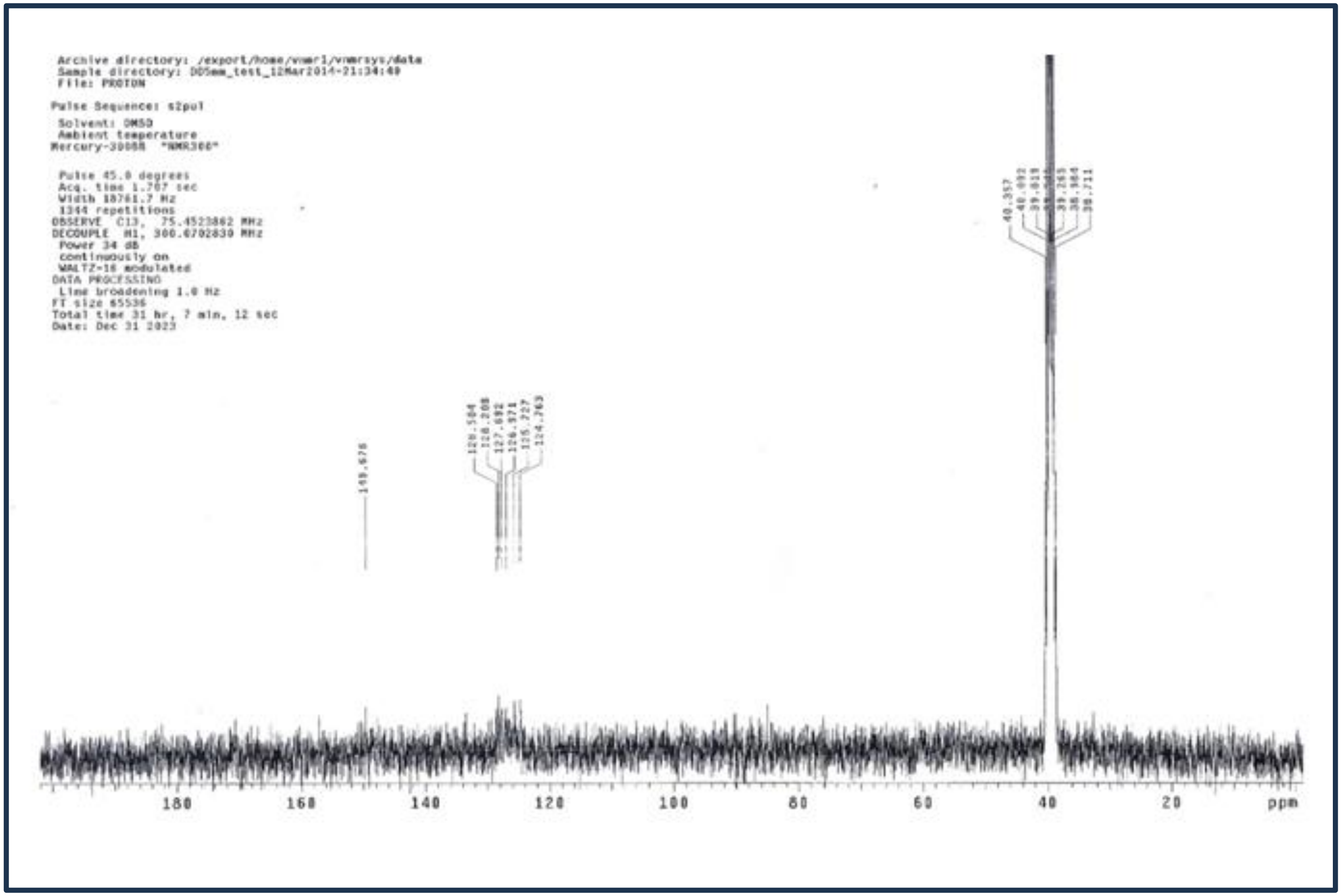

Figure S20. ^{13}C NMR (300 MHz, DMSO-d_6) spectrum of compound **11**

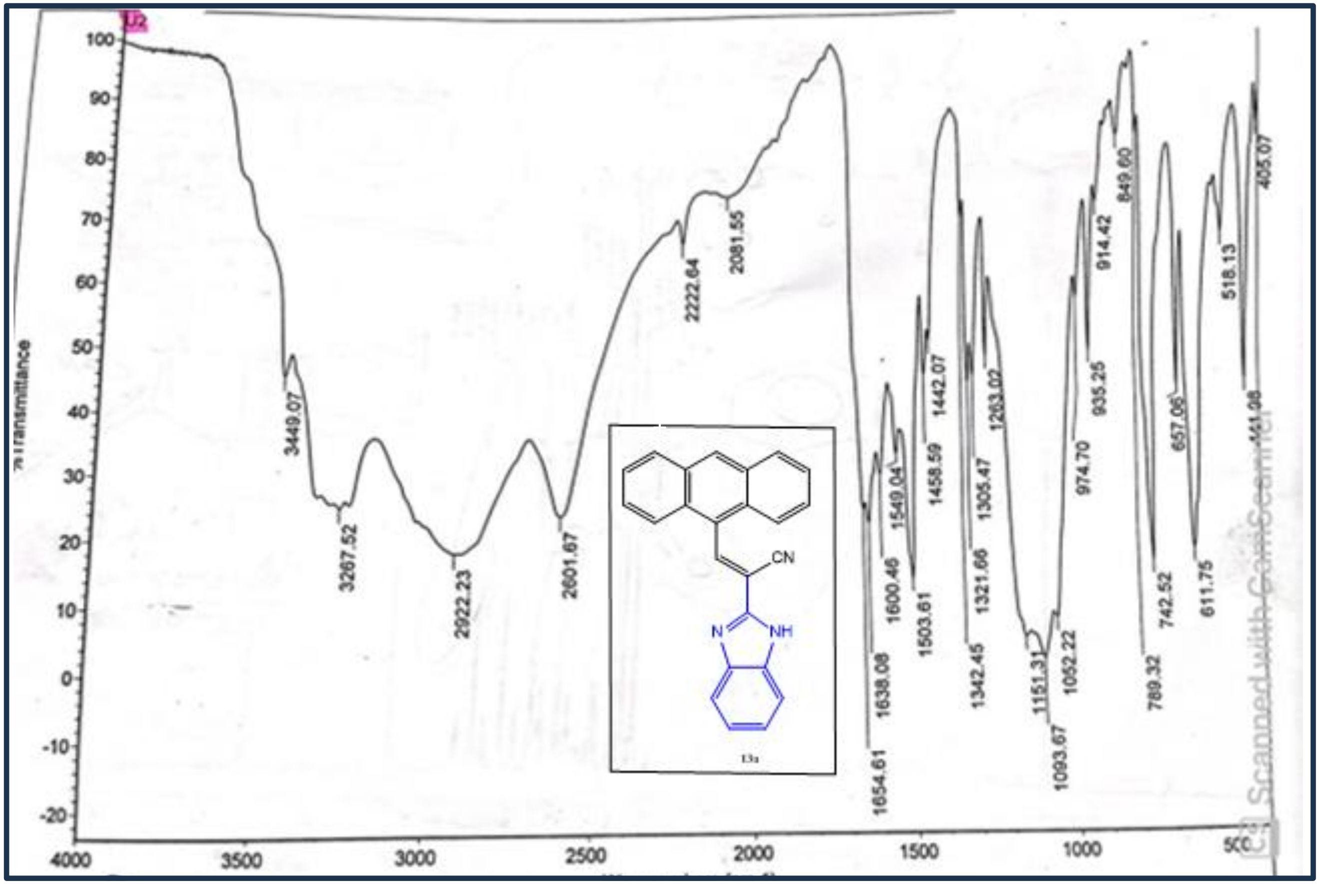

Figure S21. IR spectrum of compound **13a**

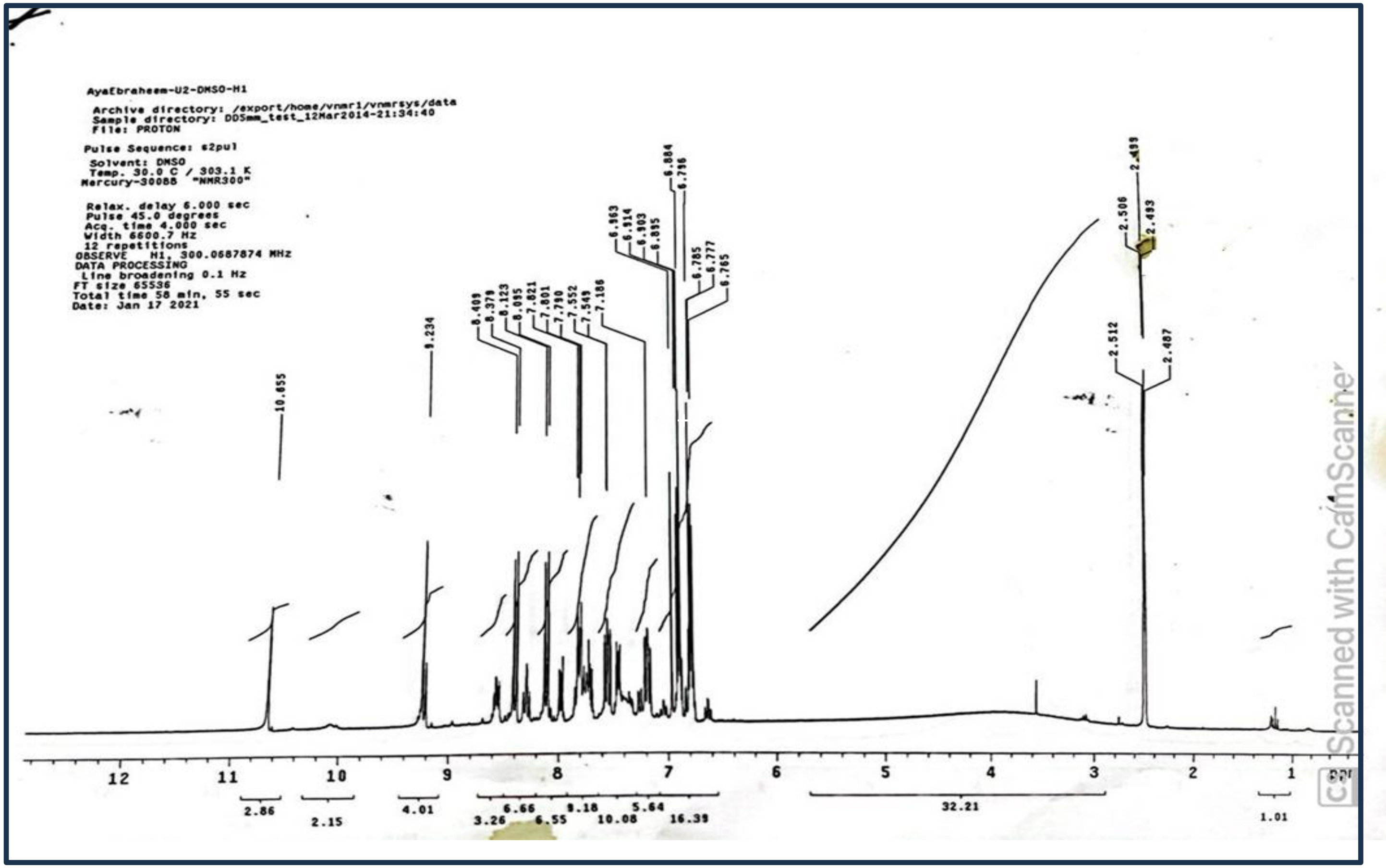

Figure S22. ^{1}H NMR (300 MHz, DMSO-d_6) spectrum of compound **13a**

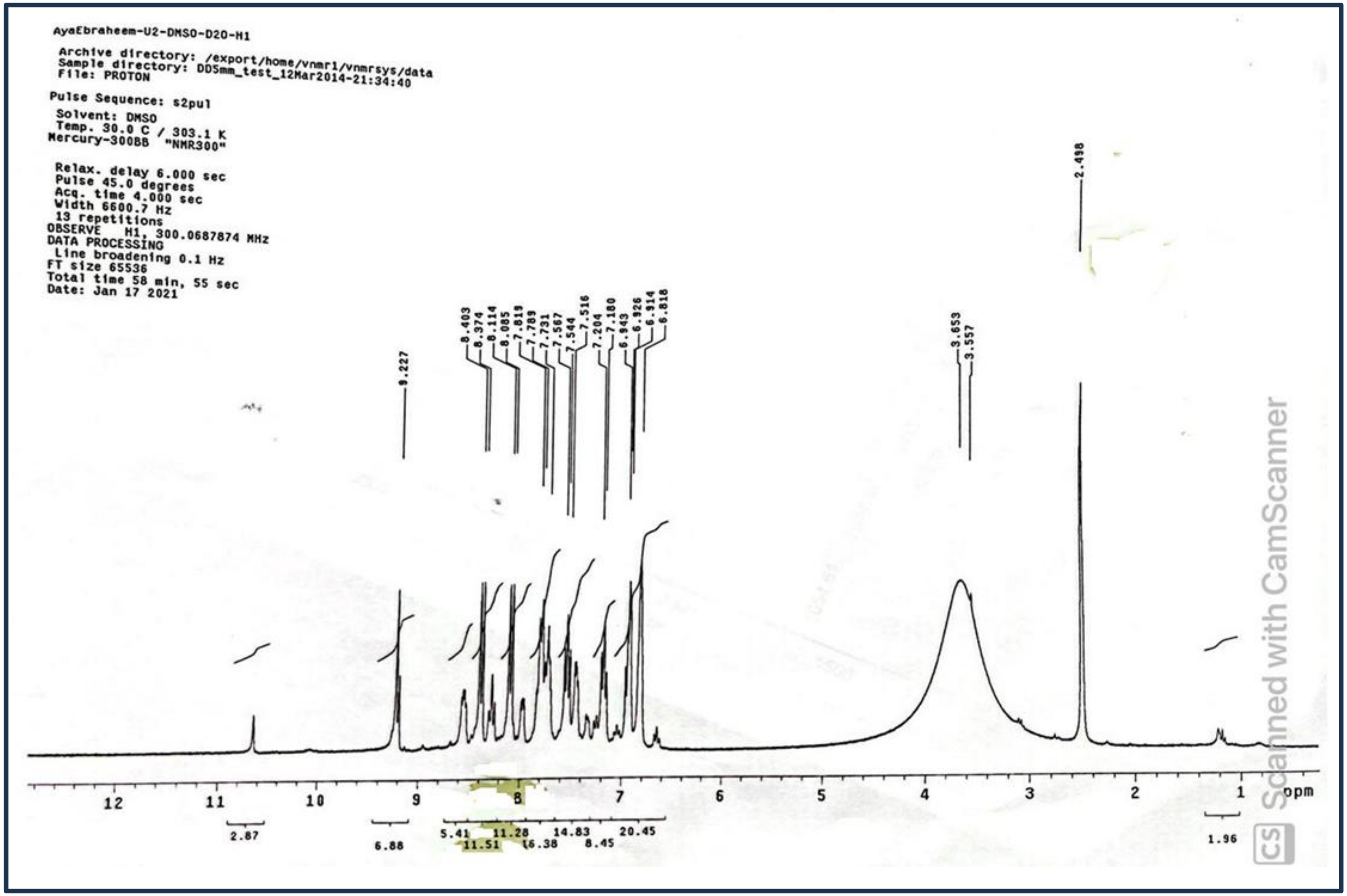
AyaEbraheem-U2-DMSO-D2O-H1
Archive directory: /export/home/vnmr1/vnmrsys/data
Sample directory: DD5mm_test_12Mar2014-21:34:40
File: PROTON
Pulse Sequence: s2pul
Solvent: DMSO
Temp. 30.0 C / 303.1 K
Mercury-300BB "NMR300"
Relax. delay 6.000 sec
Pulse 45.0 degrees
Acq. time 4.000 sec
Width 6600.7 Hz
13 repetitions
OBSERVE H1, 300.0687874 MHz
DATA PROCESSING
Line broadening 0.1 Hz
FT size 65536
Total time 58 min, 55 sec
Date: Jan 17 2021
9.227
8.403
8.374
8.114
8.085
7.819
7.789
7.731
7.567
7.544
7.516
7.204
7.180
6.943
6.926
6.914
6.818
3.653
3.557
2.498
12
11
10
9
8
7
6
5
4
3
2
1
ppm
2.87
6.88
5.41
11.51
11.28
16.38
14.83
8.45
20.45
1.96

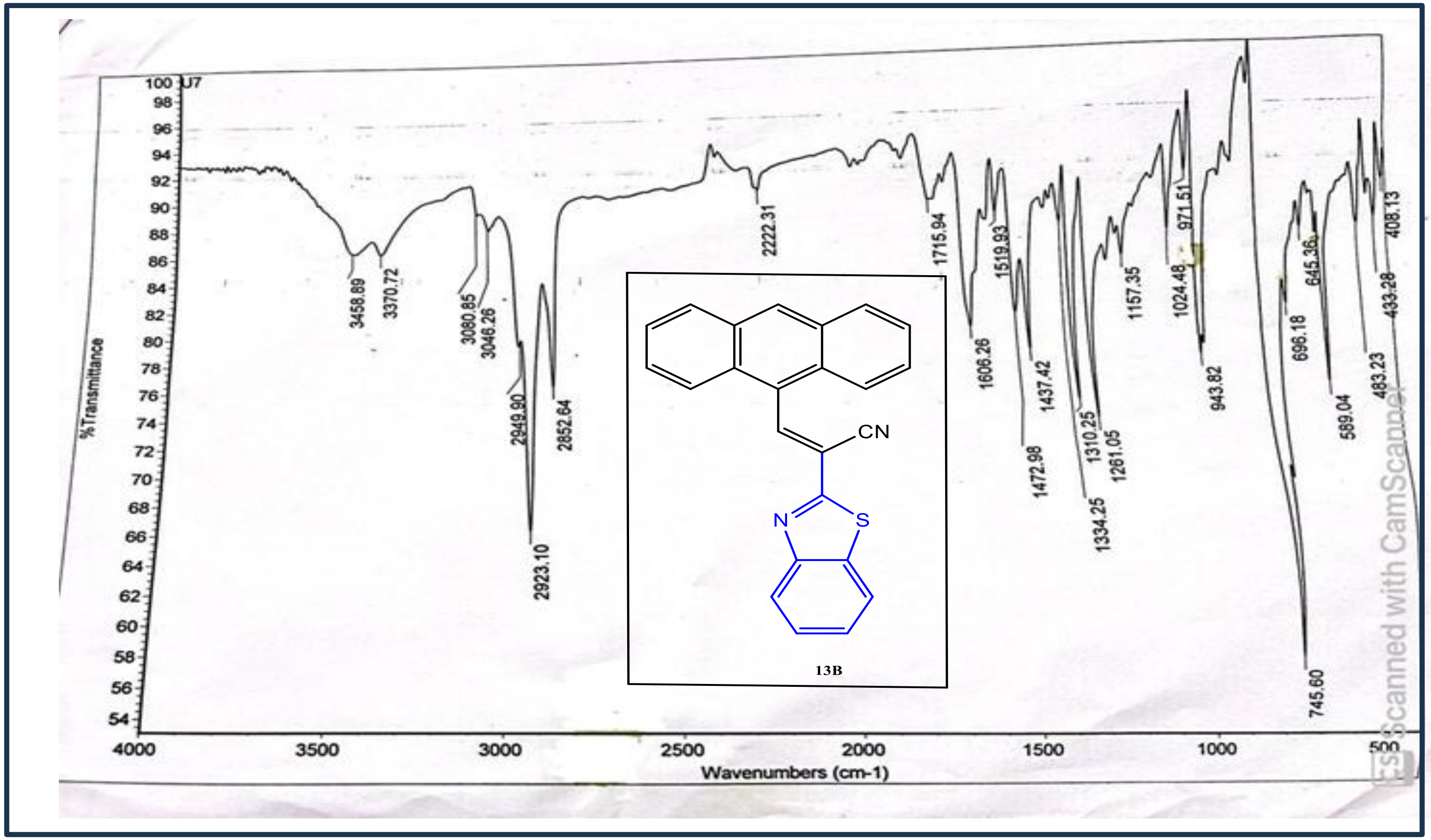

Figure S23. IR spectrum of compound **13b**

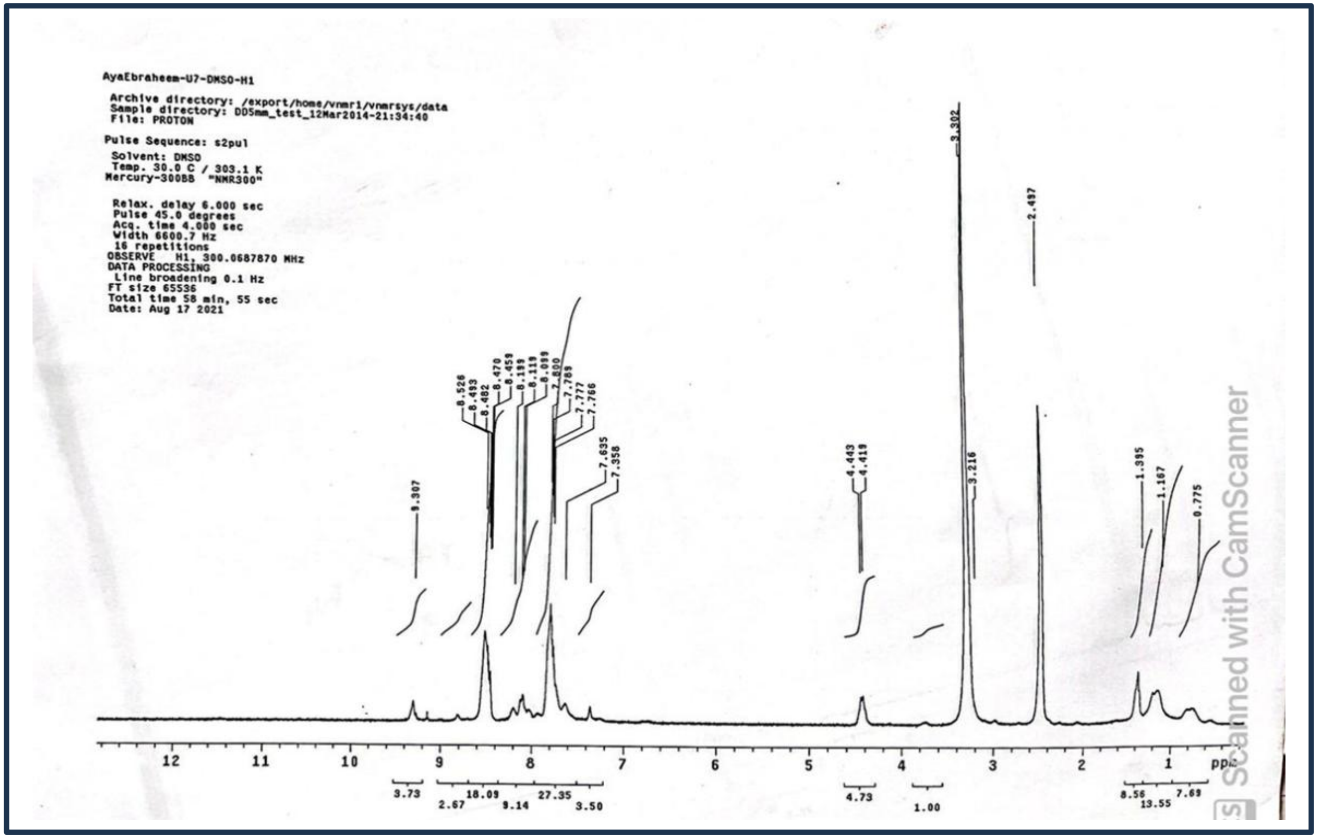

Figure S24. ^{1}H NMR (300 MHz, DMSO-d_6) spectrum of compound 13b

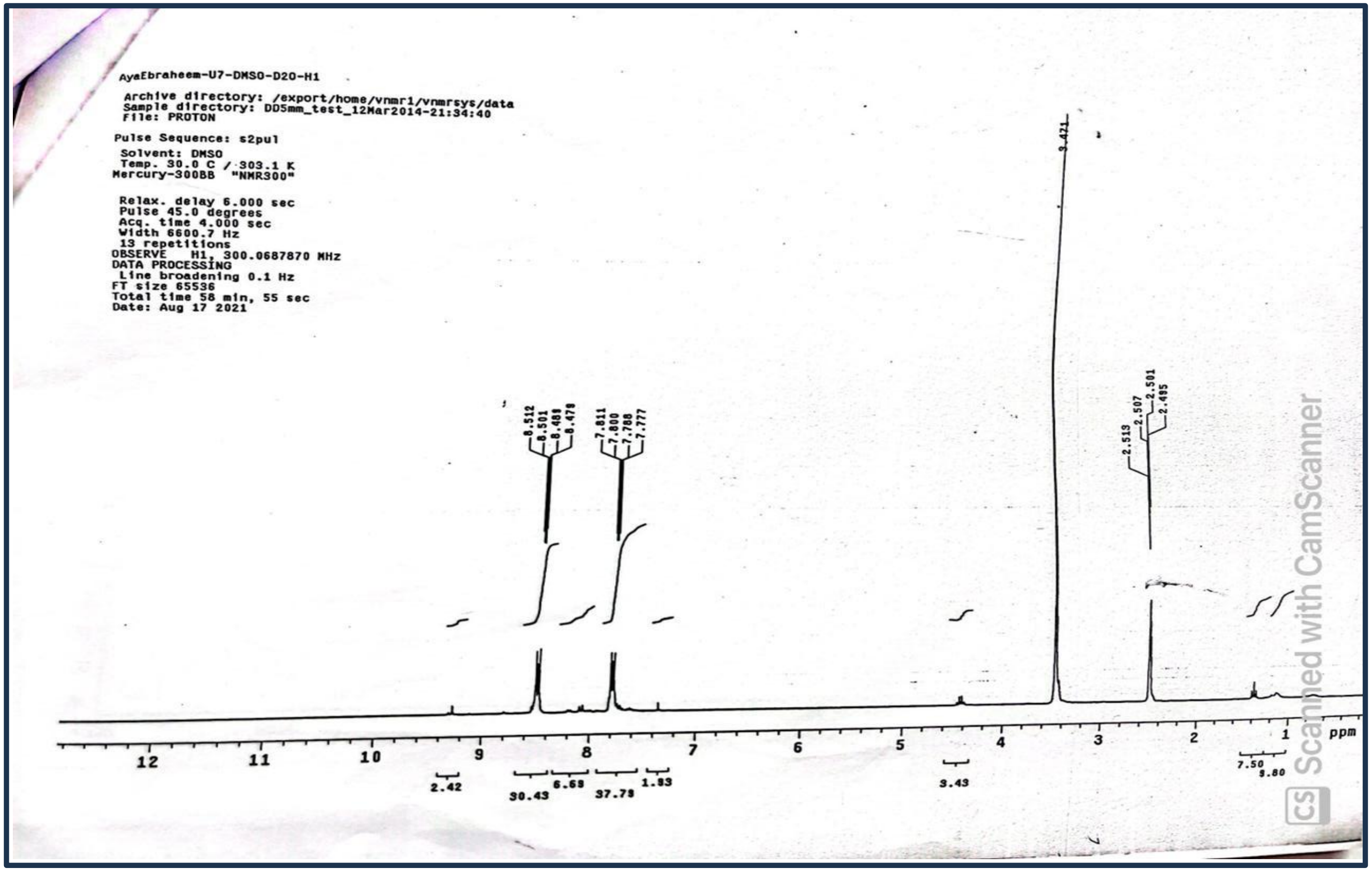

Scanned with CamScanner

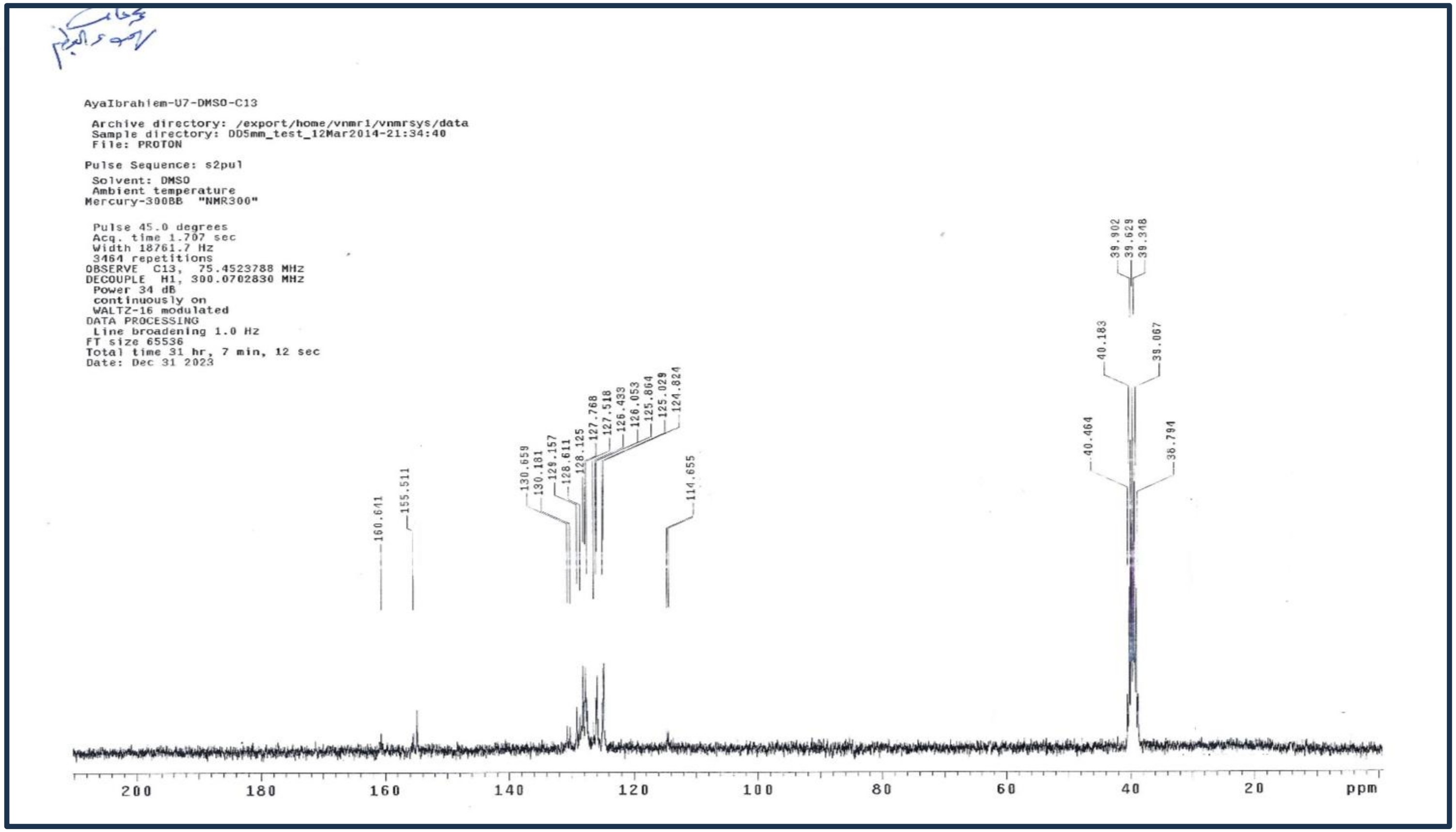

Figure S25. ^{13}C NMR (300 MHz, DMSO-d_6) spectrum of compound **13b**

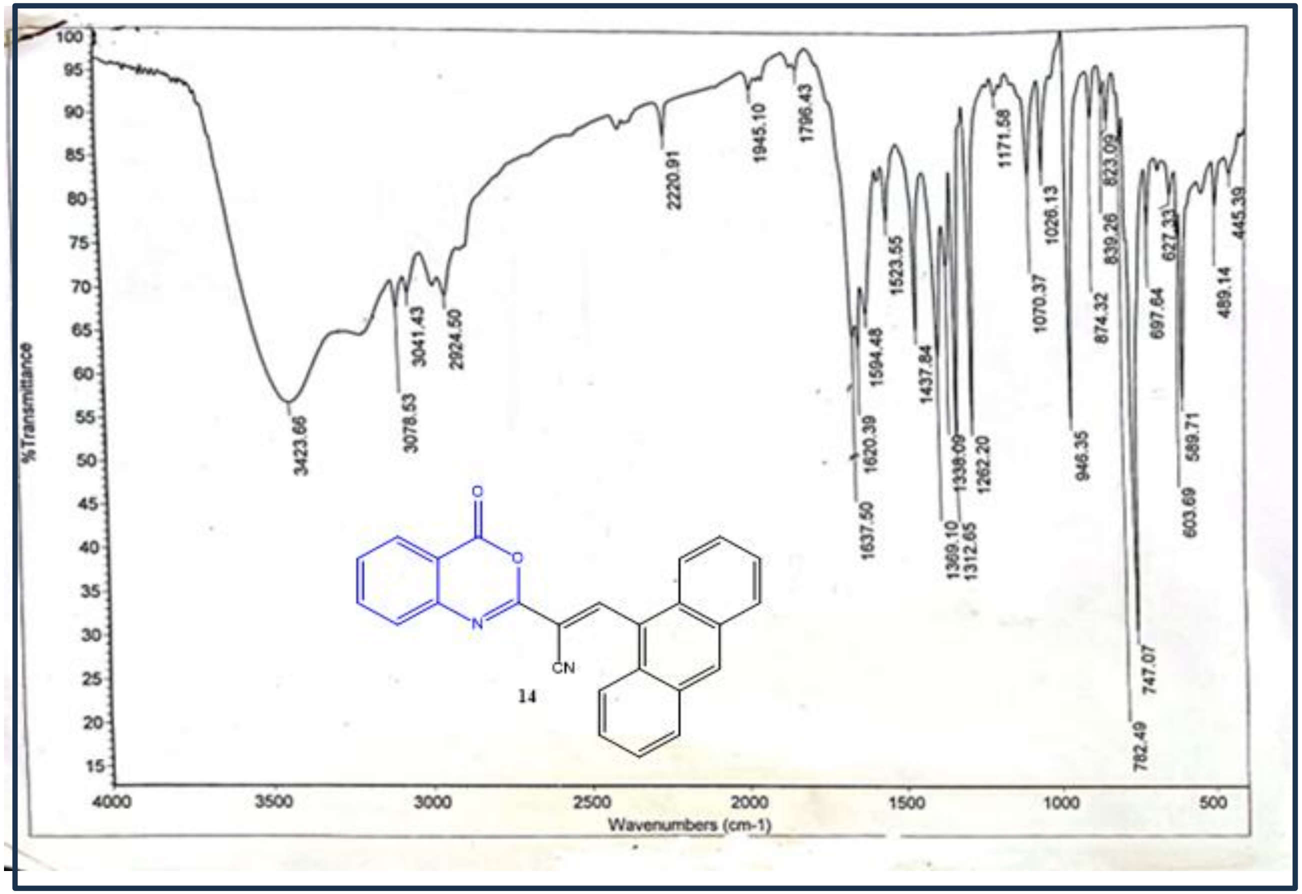

Figure S26. IR spectrum of compound **14**

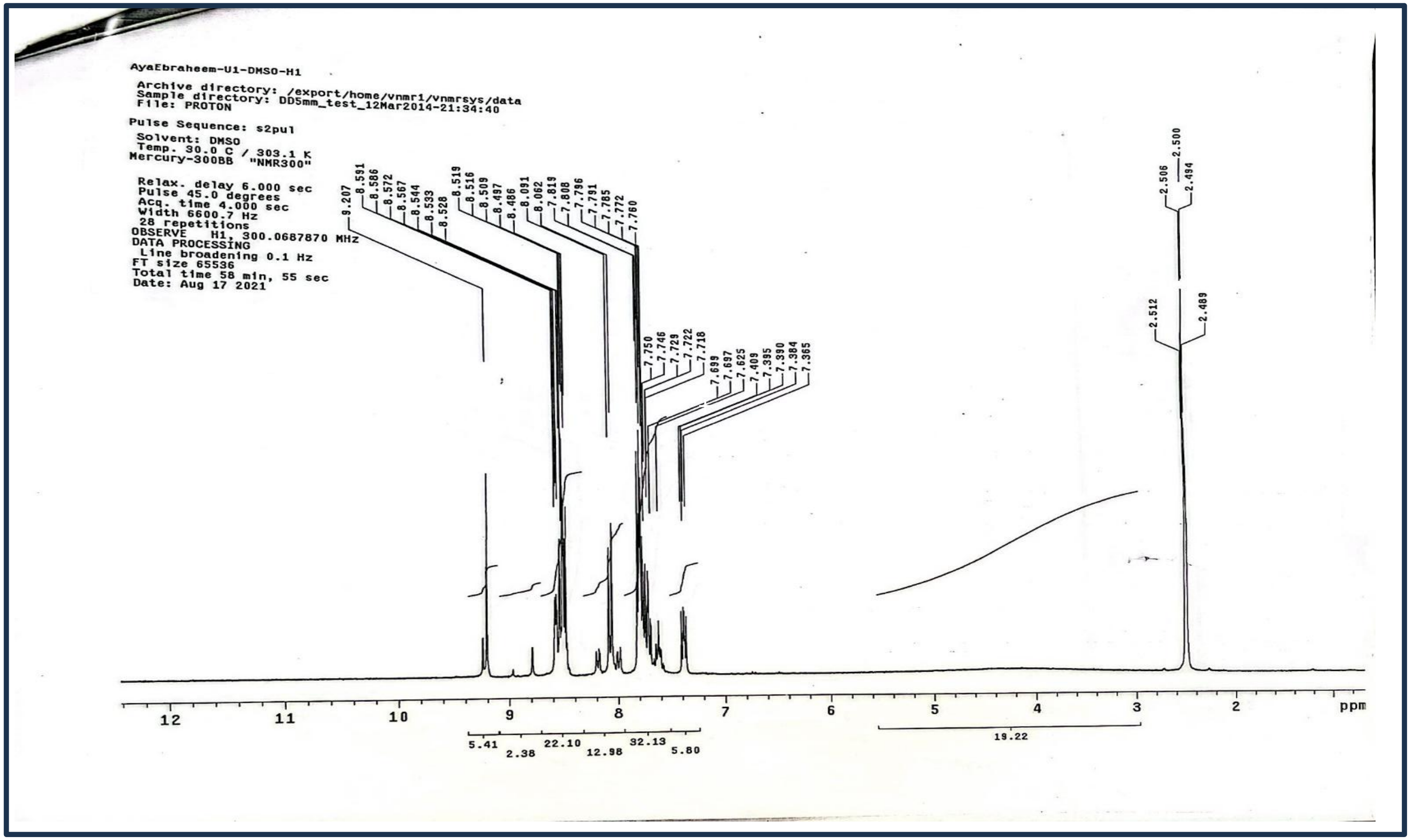

Figure S27. ^{1}H NMR (300 MHz, DMSO-d_6) spectrum of compound 14

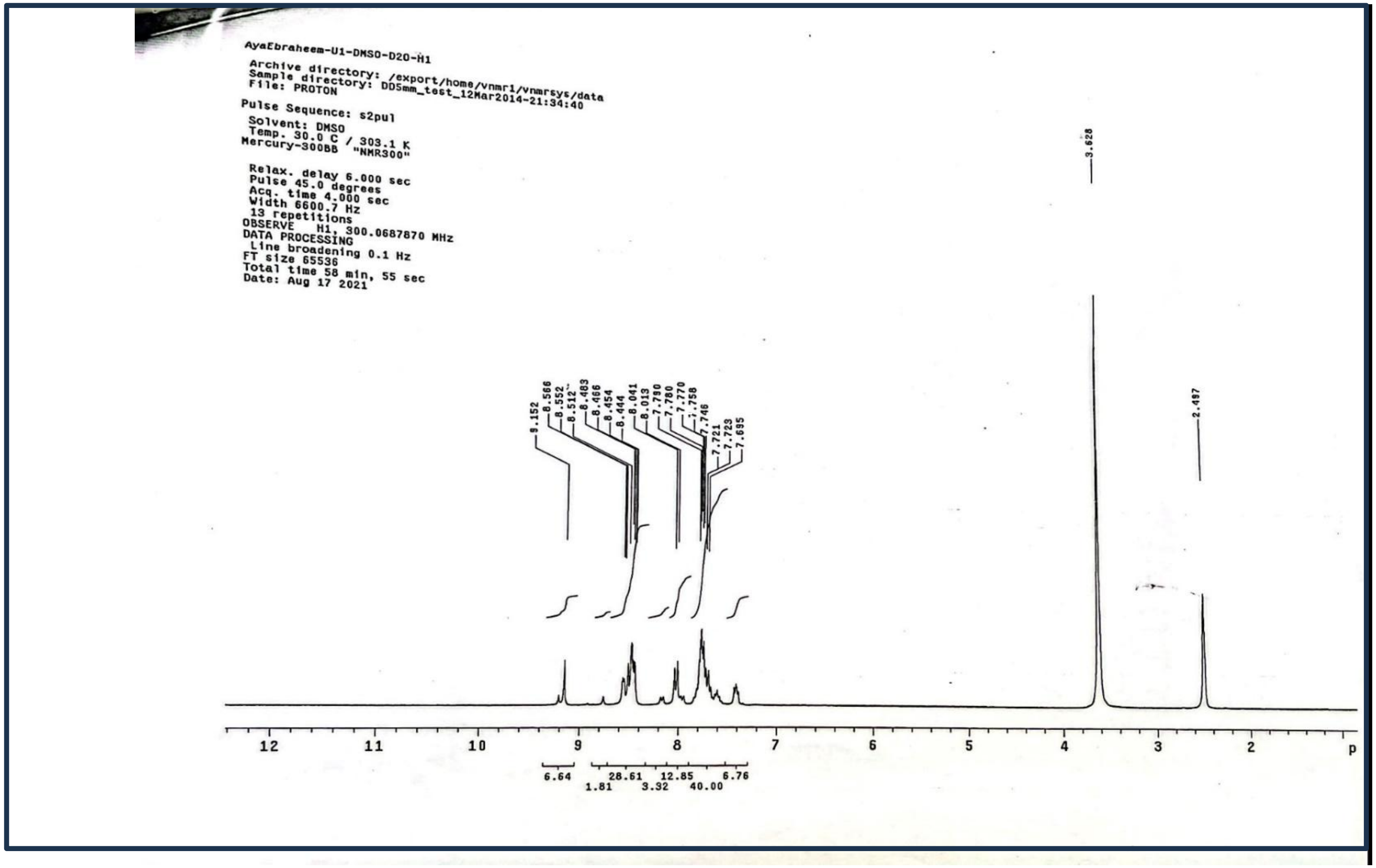

AyaEbraheem-U1-DMSO-D2O-H1
Archive directory: /export/home/vnmr1/vnmrsys/data
Sample directory: DD5mm_test_12Mar2014-21:34:40
File: PROTON
Pulse Sequence: s2pul
Solvent: DMSO
Temp. 30.0 C / 303.1 K
Mercury-300BB "NMR300"
Relax. delay 6.000 sec
Pulse 45.0 degrees
Acq. time 4.000 sec
Width 6600.7 Hz
13 repetitions
OBSERVE H1, 300.0687870 MHz
DATA PROCESSING
Line broadening 0.1 Hz
FT size 65536
Total time 58 min, 55 sec
Date: Aug 17 2021
9.152
8.566
8.552
8.512
8.483
8.466
8.454
8.444
8.041
8.013
7.790
7.780
7.770
7.758
7.746
7.721
7.723
7.695
3.628
2.497
12
11
10
9
8
7
6
5
4
3
2
p
6.64
1.81
28.61
3.32
12.85
40.00
6.76

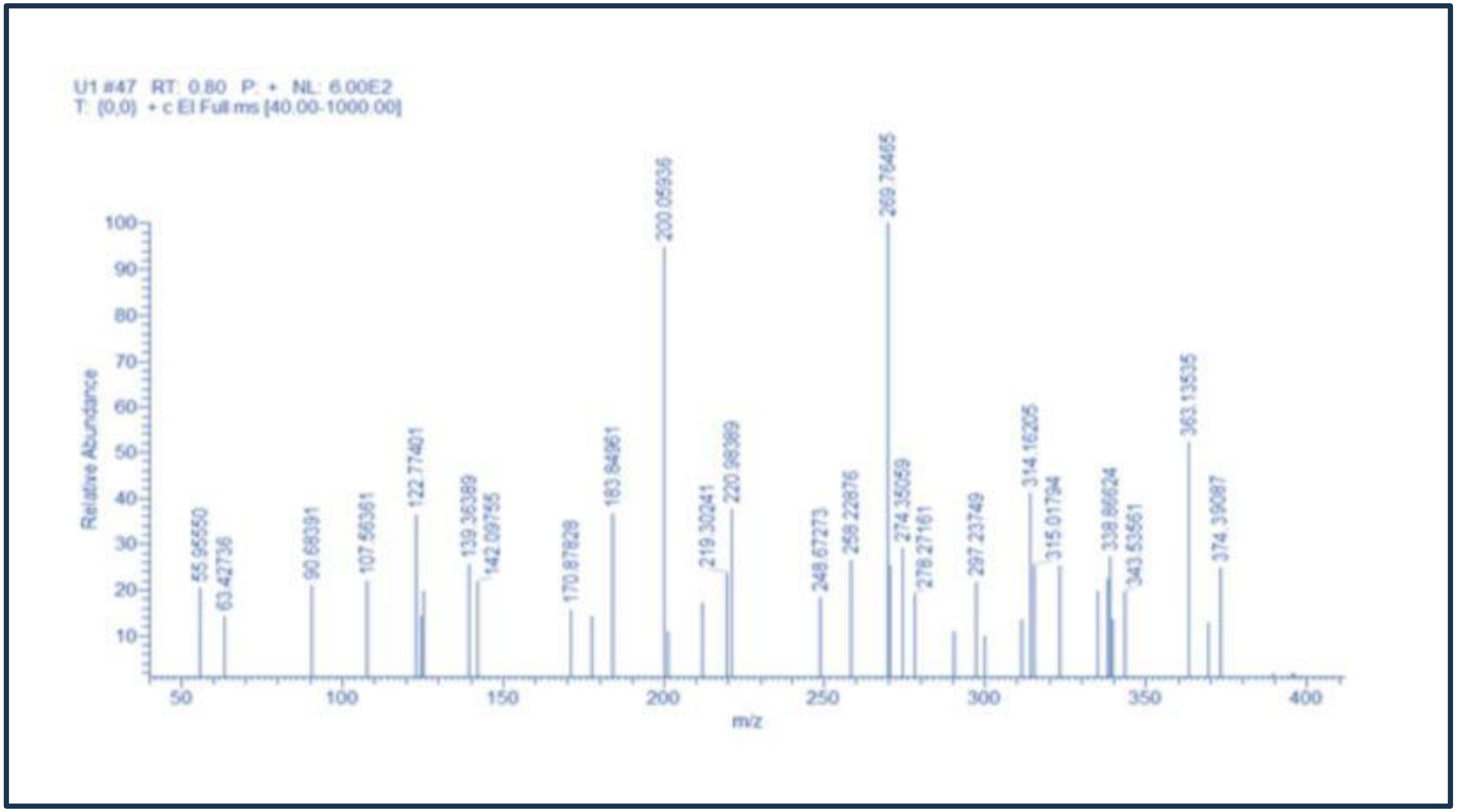

Figure S28. HR-Mass spectrum of compound **14**

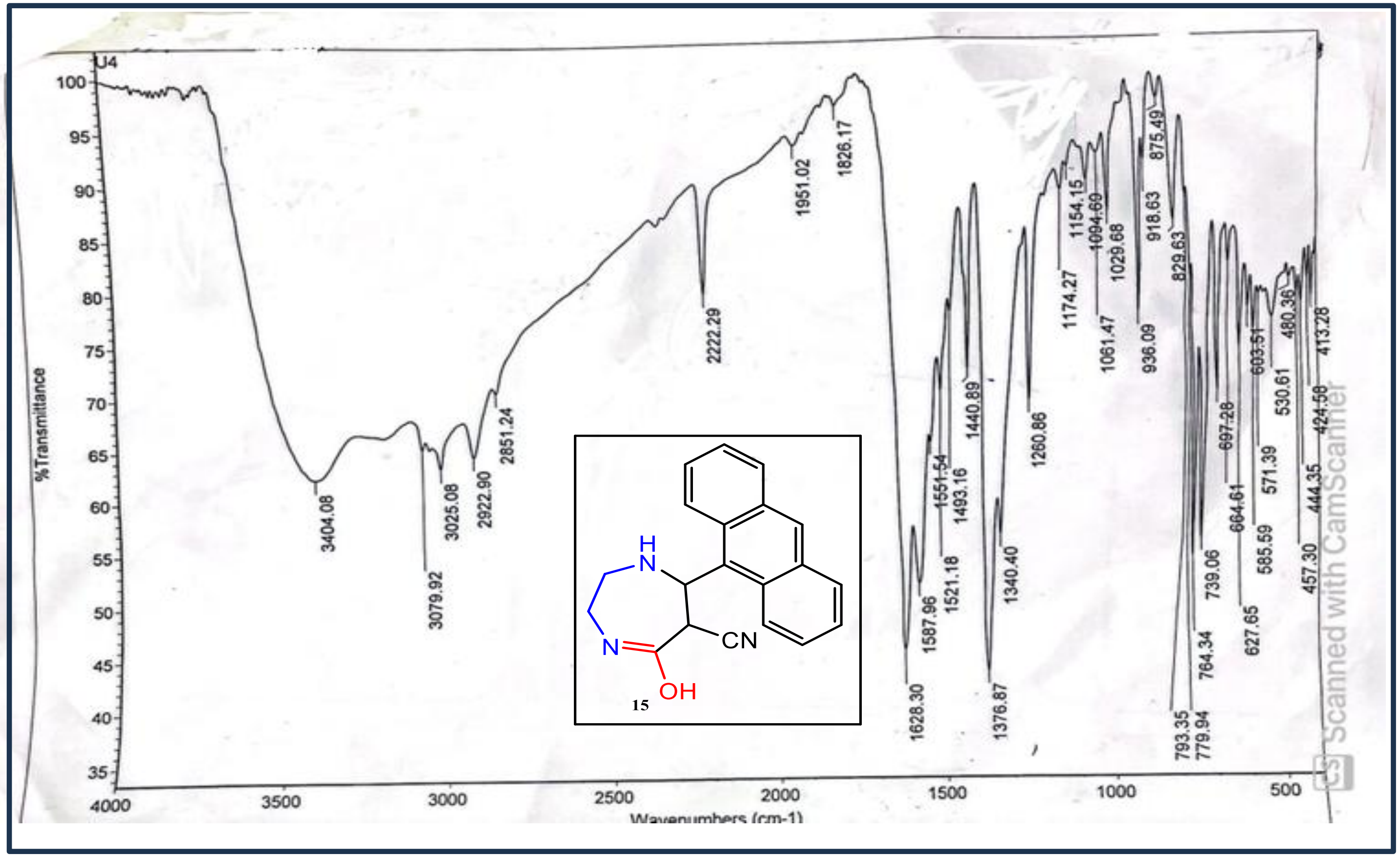

Figure S29. IR spectrum of compound **15**

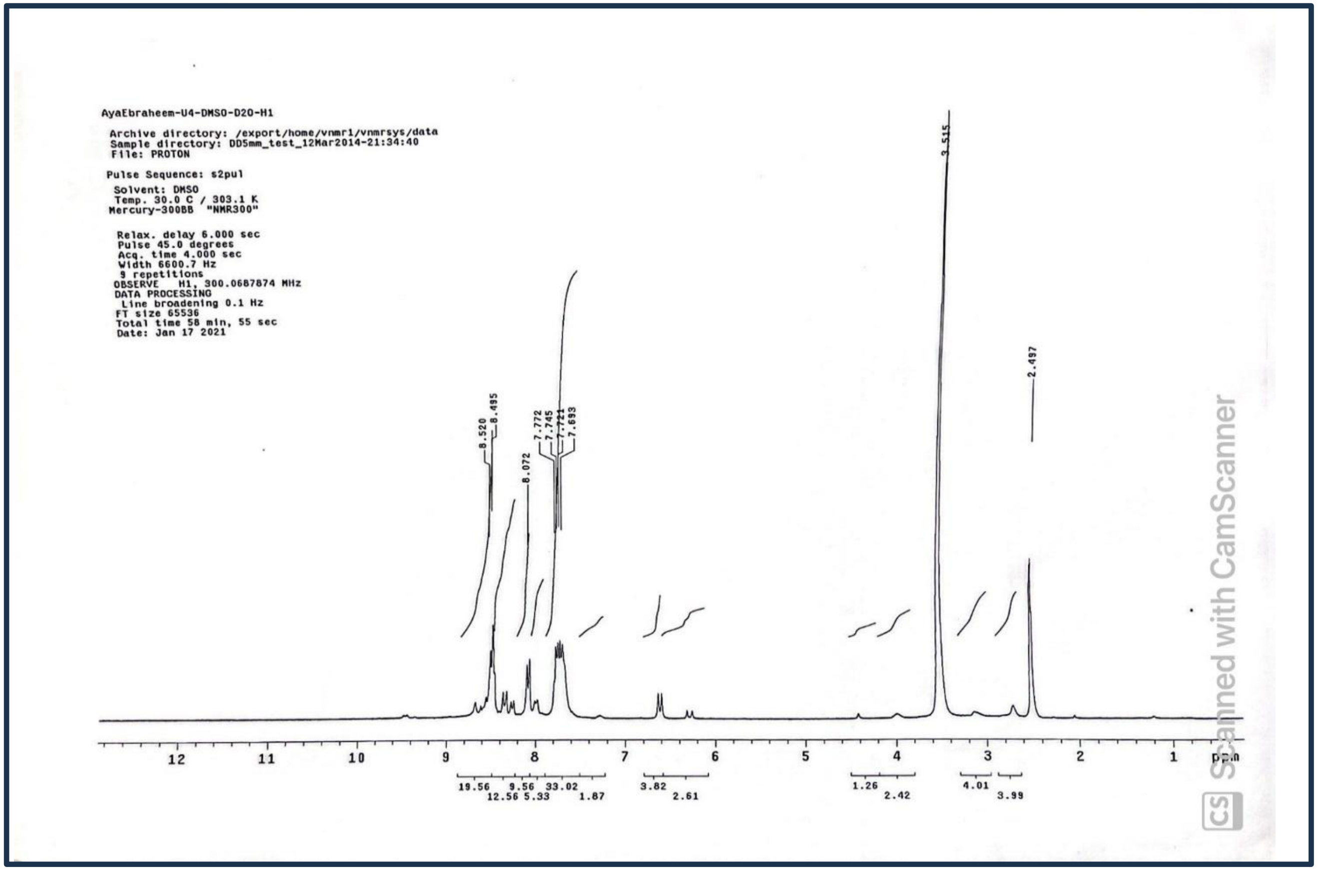

Scanned with CamScanner

Figure S30. ^{1}H NMR (300 MHz, DMSO-d_6) spectrum of compound 15

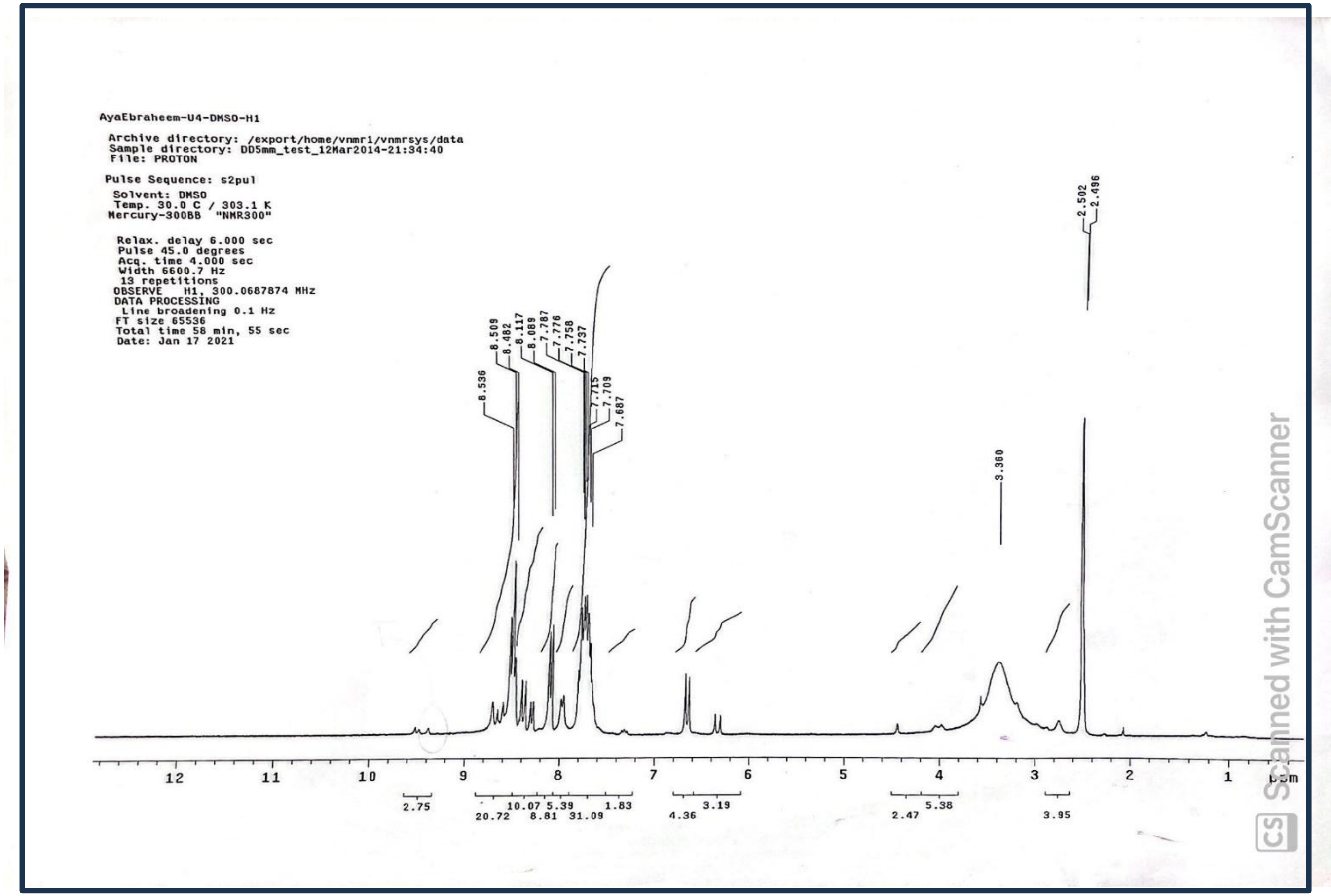

AyaEbraheem-U4-DMSO-H1
Archive directory: /export/home/vnmr1/vnmrsys/data
Sample directory: DD5mm_test_12Mar2014-21:34:40
File: PROTON
Pulse Sequence: s2pul
Solvent: DMSO
Temp. 30.0 C / 303.1 K
Mercury-300BB "NMR300"
Relax. delay 6.000 sec
Pulse 45.0 degrees
Acq. time 4.000 sec
Width 6600.7 Hz
13 repetitions
OBSERVE H1, 300.0687874 MHz
DATA PROCESSING
Line broadening 0.1 Hz
FT size 65536
Total time 58 min, 55 sec
Date: Jan 17 2021
8.536
8.509
8.482
8.117
8.089
7.787
7.776
7.758
7.737
7.715
7.709
7.687
3.360
2.502
2.496
12 11 10 9 8 7 6 5 4 3 2 1 ppm
2.75
20.72
10.07
8.81
5.39
31.09
1.83
4.36
3.19
2.47
5.38
3.95
Scanned with CamScanner

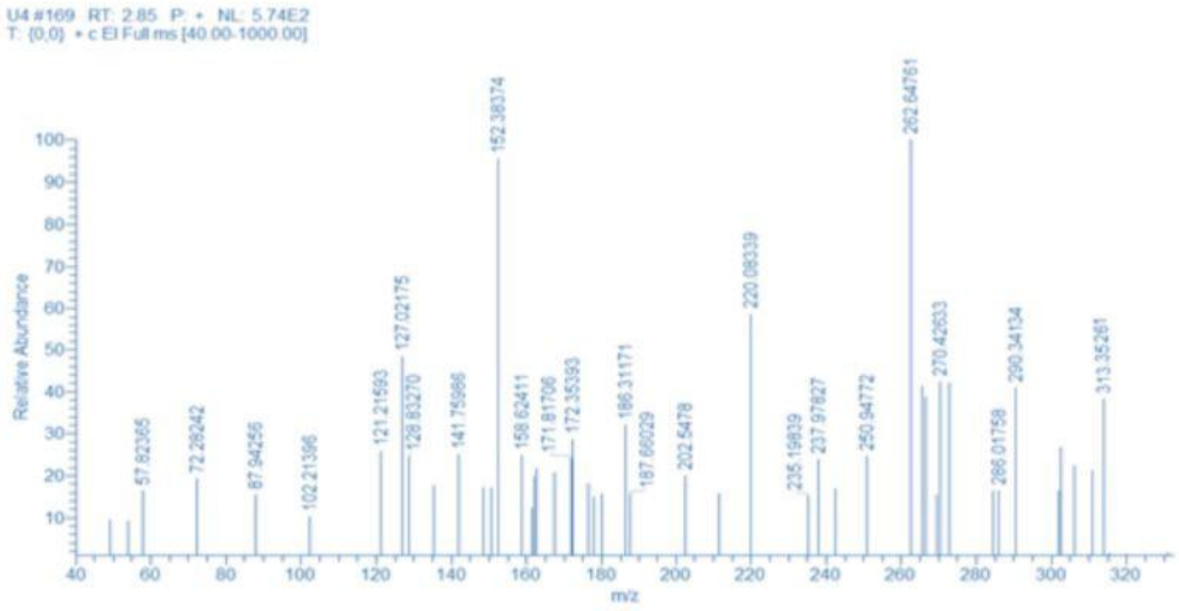

Figure S31. HR-Mass spectrum of compound **15**

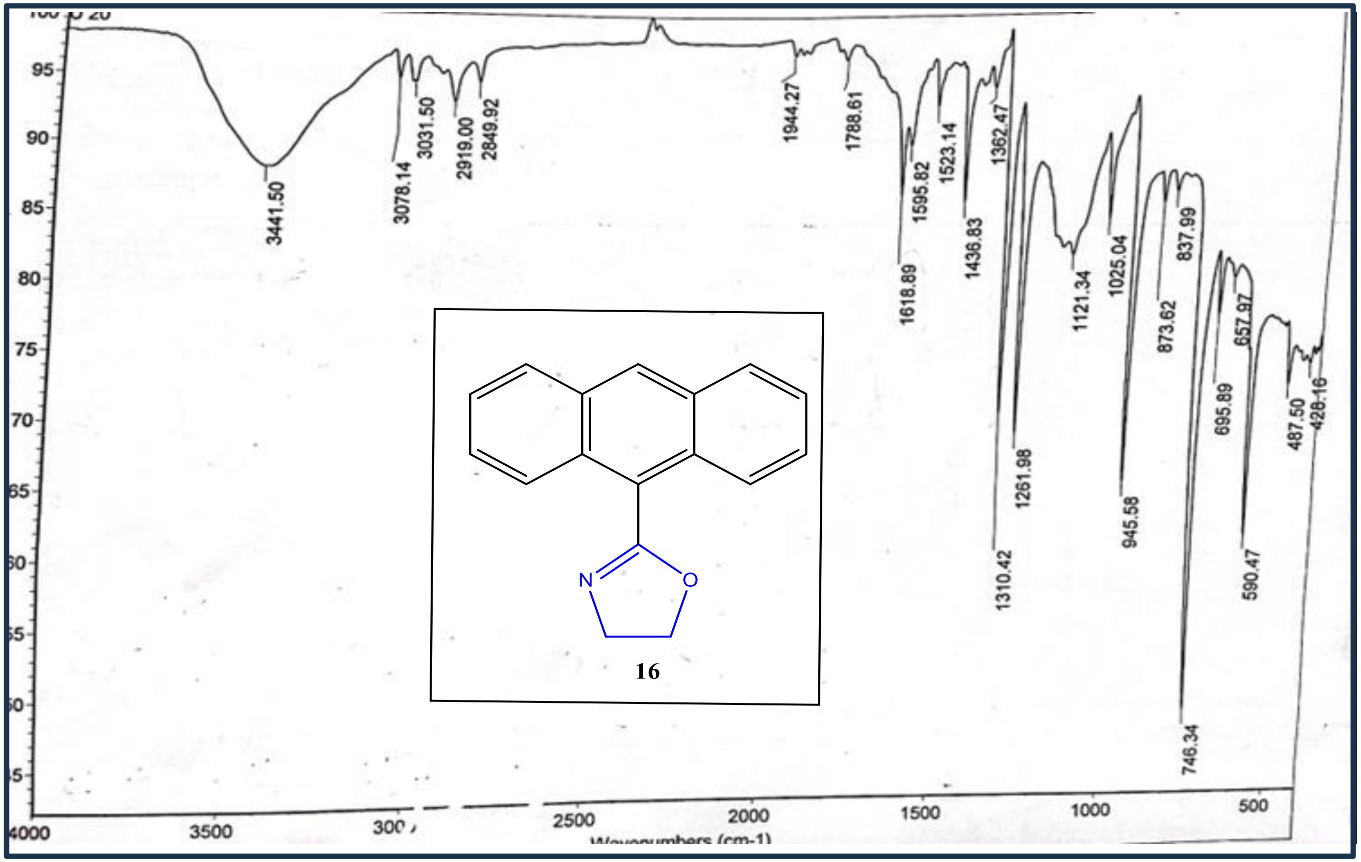

Figure S32. IR spectrum of compound **16**

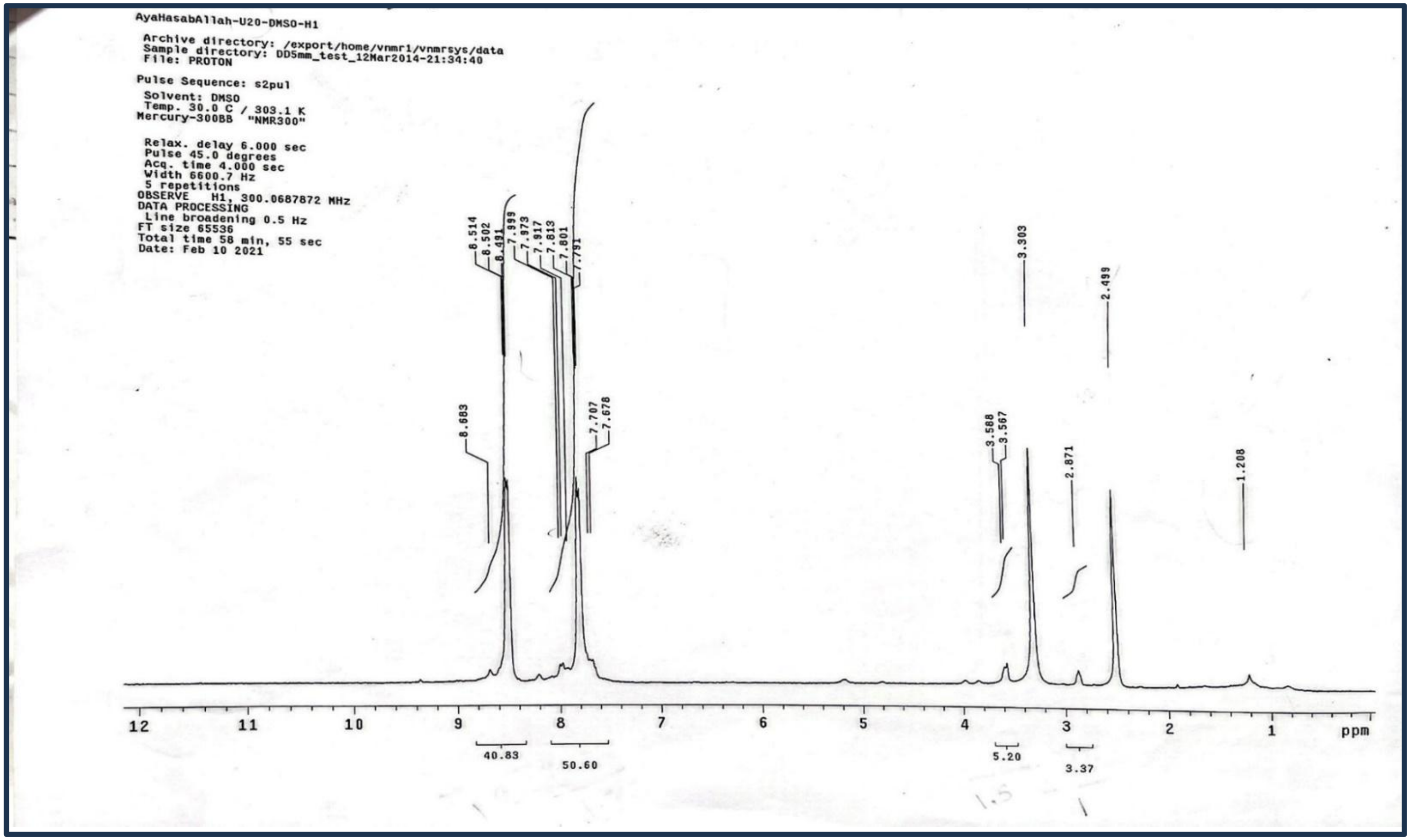

Figure S33. ^{1}H NMR (300 MHz, DMSO-d_6) spectrum of compound **16**

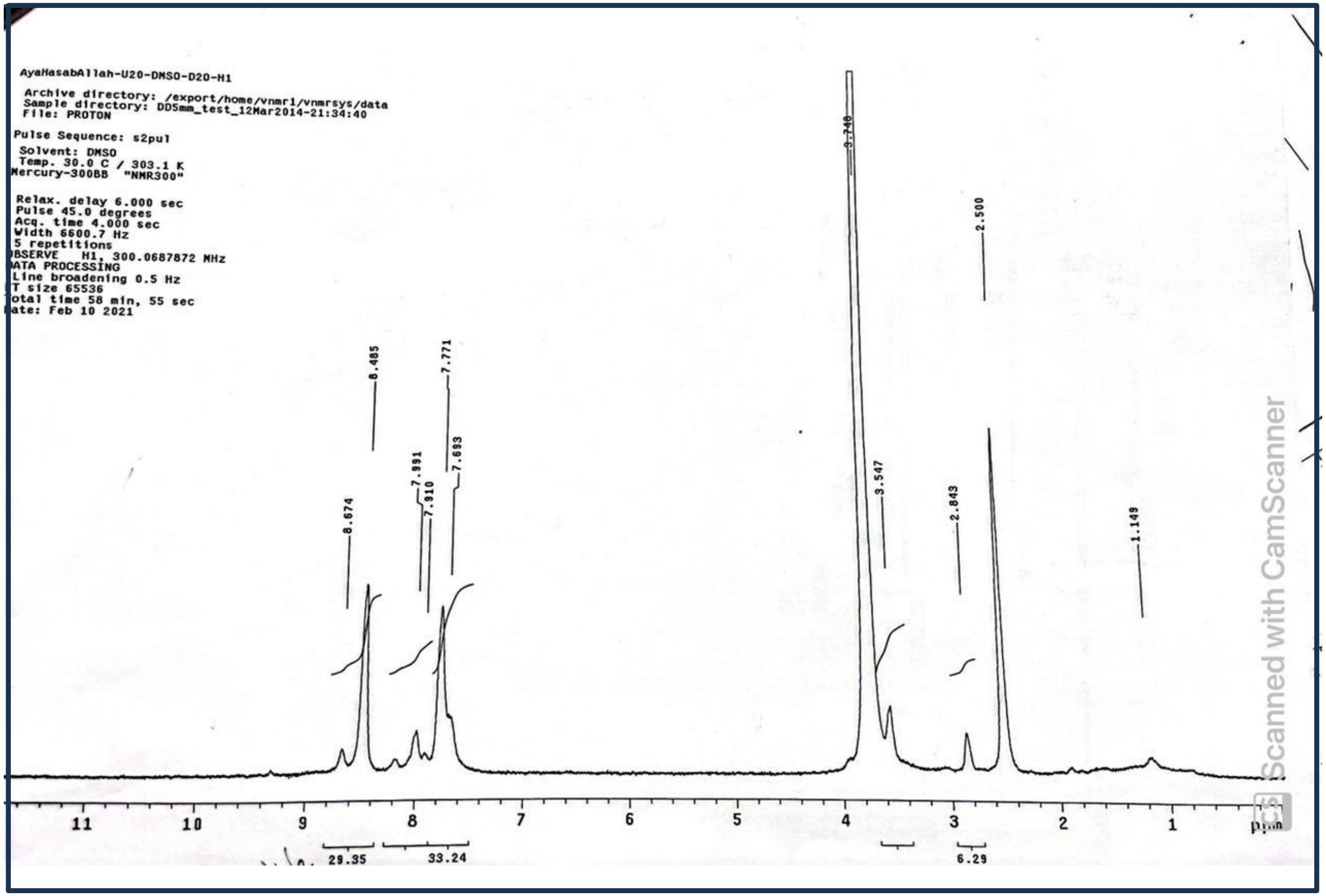
AyaHasabAllah-U20-DMSO-D20-H1
Archive directory: /export/home/vnmr1/vnmrsys/data
Sample directory: DD5mm_test_12Mar2014-21:34:40
File: PROTON
Pulse Sequence: s2pul
Solvent: DMSO
Temp. 30.0 C / 303.1 K
Mercury-300BB "NMR300"
Relax. delay 6.000 sec
Pulse 45.0 degrees
Acq. time 4.000 sec
Width 6600.7 Hz
5 repetitions
OBSERVE H1, 300.0687872 MHz
DATA PROCESSING
Line broadening 0.5 Hz
FT size 65536
Total time 58 min, 55 sec
Date: Feb 10 2021
8.674
8.485
7.991
7.910
7.771
7.693
3.740
3.547
2.843
2.500
1.149
29.35
33.24
6.29
ppm
Scanned with CamScanner

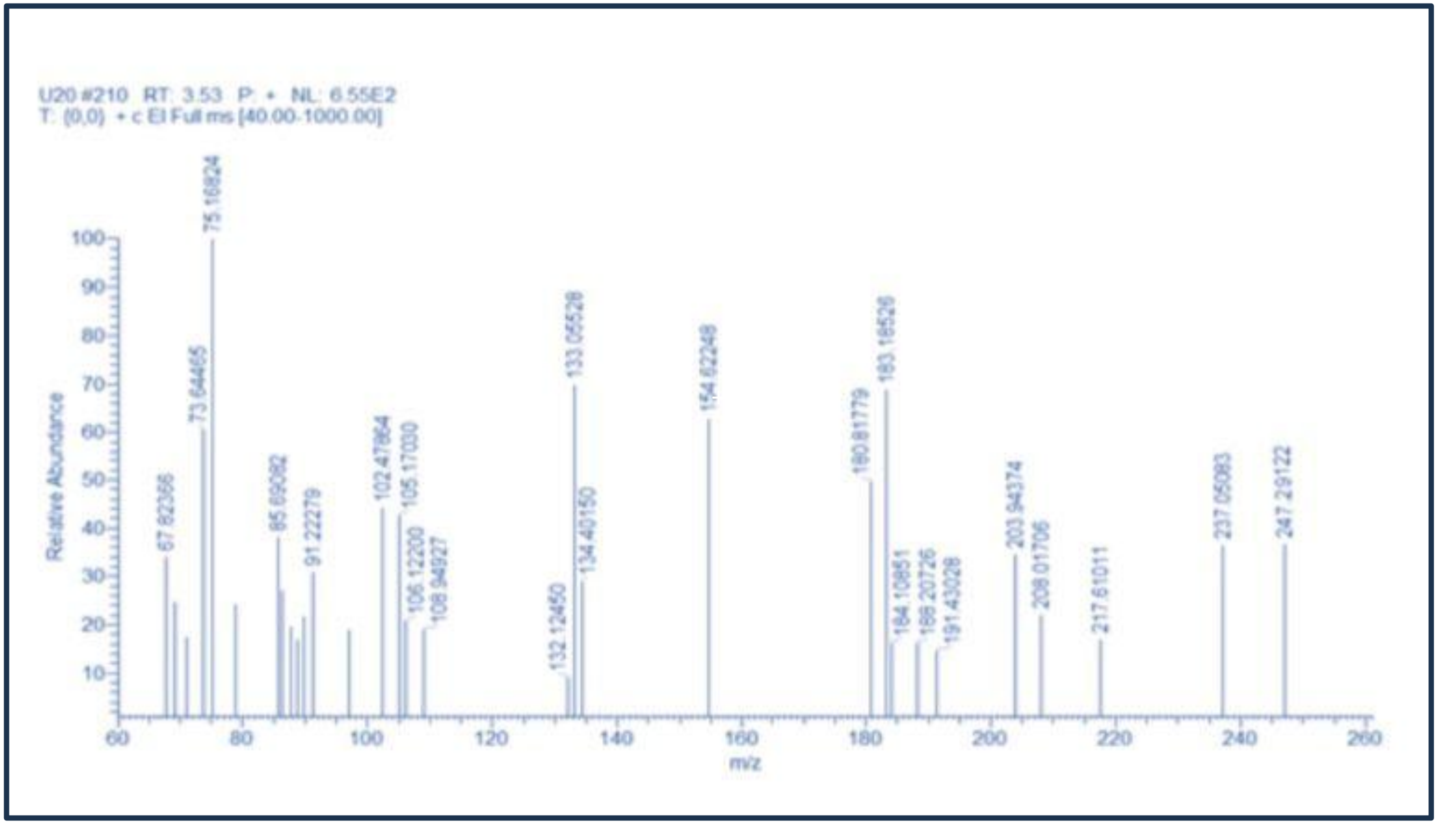

Figure S34. HR-Mass spectrum of compound **16**

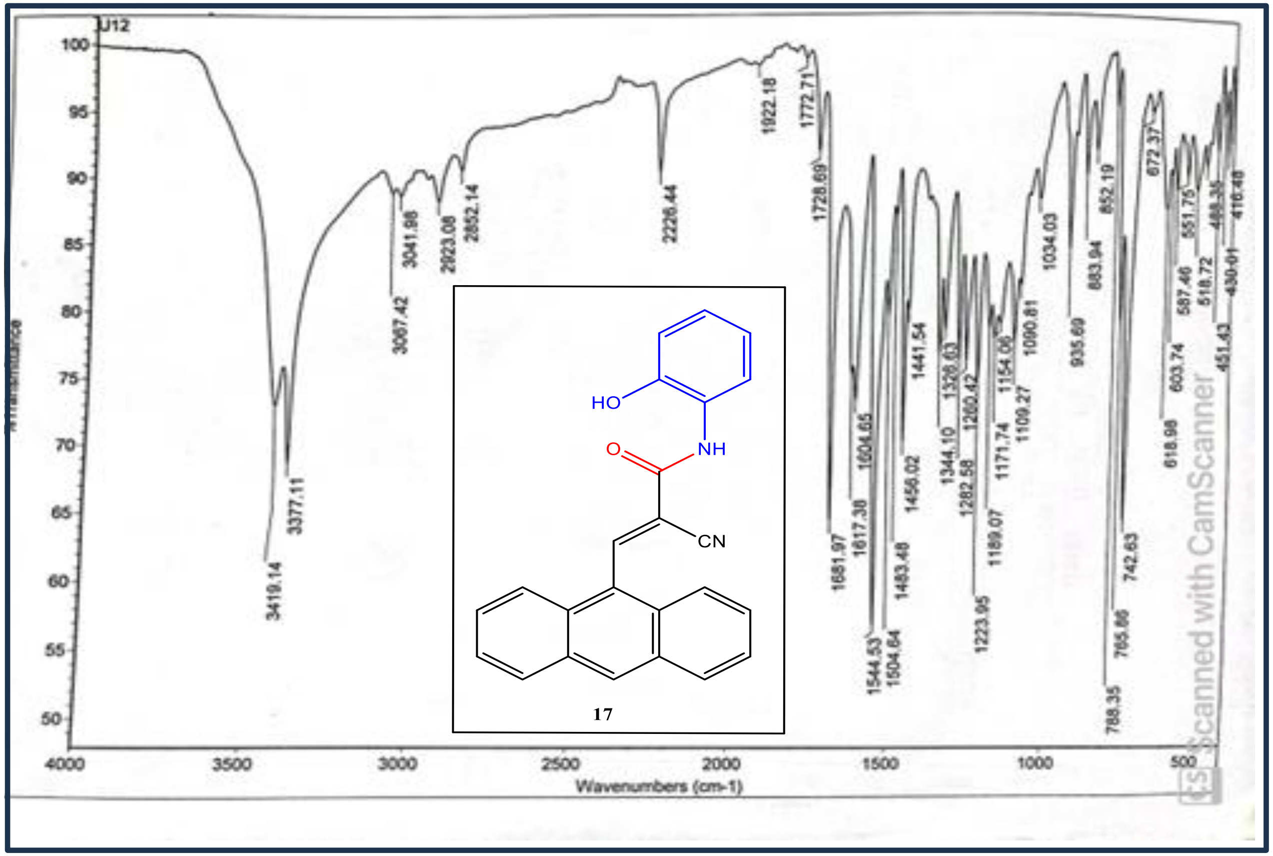

Figure S35. IR spectrum of compound **17**

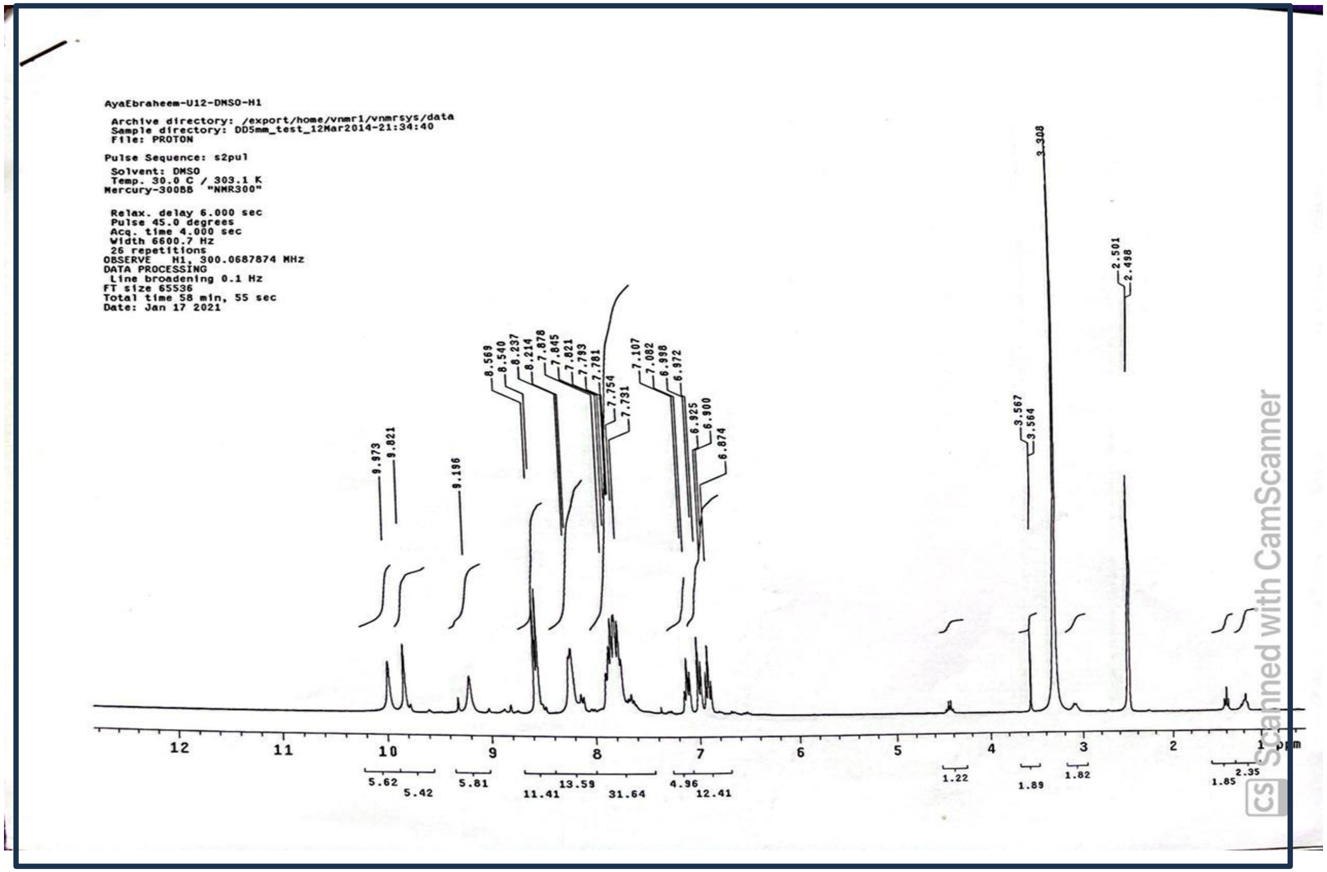

Figure S36. ^{1}H NMR (300 MHz, DMSO-d_6) spectrum of compound **17**

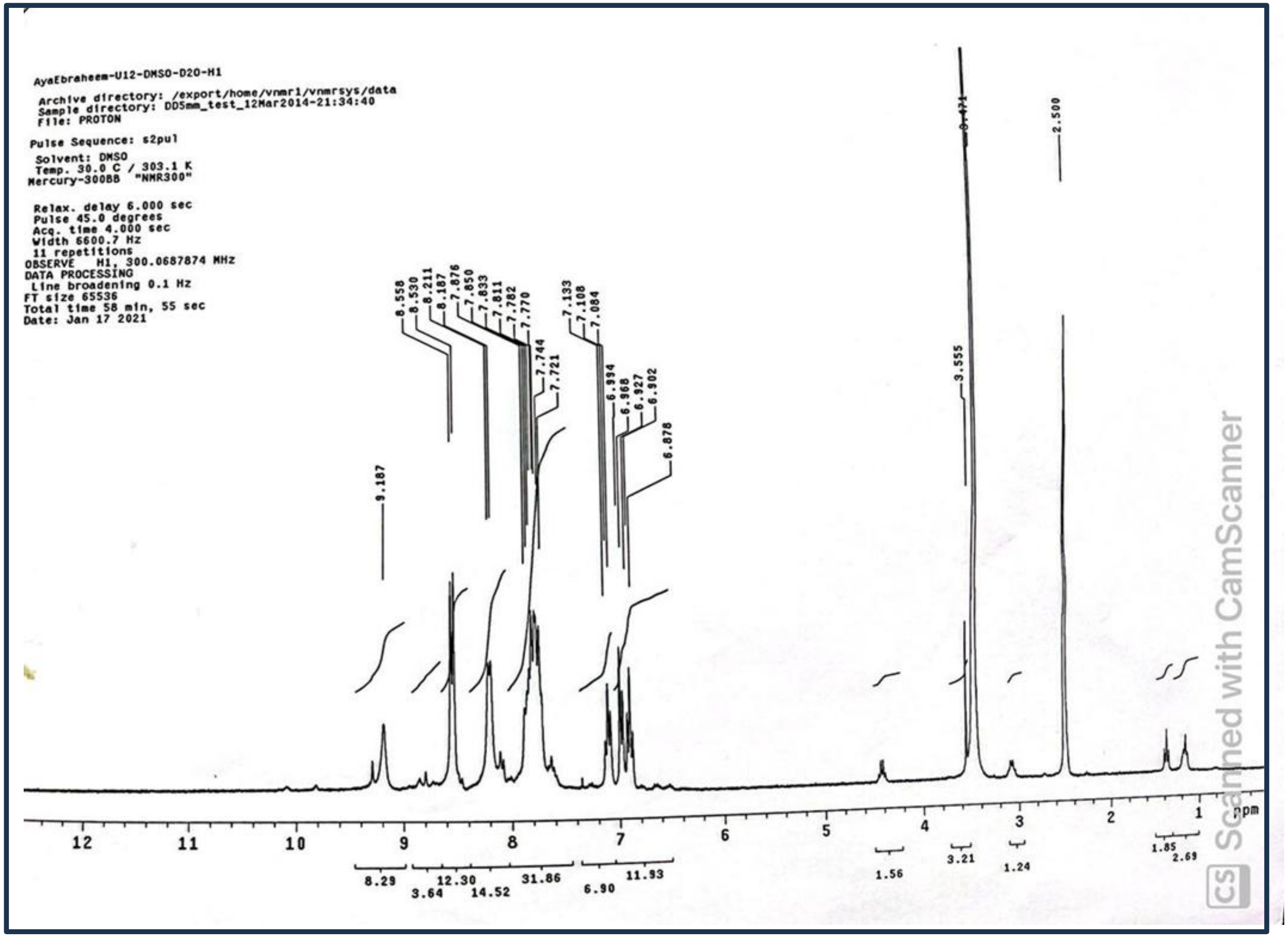
AyaEbraheem-U12-DMSO-D2O-H1
Archive directory: /export/home/vnmr1/vnmrsys/data
Sample directory: DD5mm_test_12Mar2014-21:34:40
File: PROTON
Pulse Sequence: s2pul
Solvent: DMSO
Temp. 30.0 C / 303.1 K
Mercury-300BB "NMR300"
Relax. delay 6.000 sec
Pulse 45.0 degrees
Acq. time 4.000 sec
Width 6600.7 Hz
11 repetitions
OBSERVE H1, 300.0687874 MHz
DATA PROCESSING
Line broadening 0.1 Hz
FT size 65536
Total time 58 min, 55 sec
Date: Jan 17 2021
9.187
8.558
8.530
8.211
8.187
7.876
7.850
7.833
7.811
7.782
7.770
7.744
7.721
7.133
7.108
7.084
6.994
6.968
6.927
6.902
6.878
3.555
2.500
ppm
12
11
10
9
8
7
6
5
4
3
2
1
8.29
3.64
12.30
14.52
31.86
6.90
11.93
1.56
3.21
1.24
1.85
2.69
Scanned with CamScanner

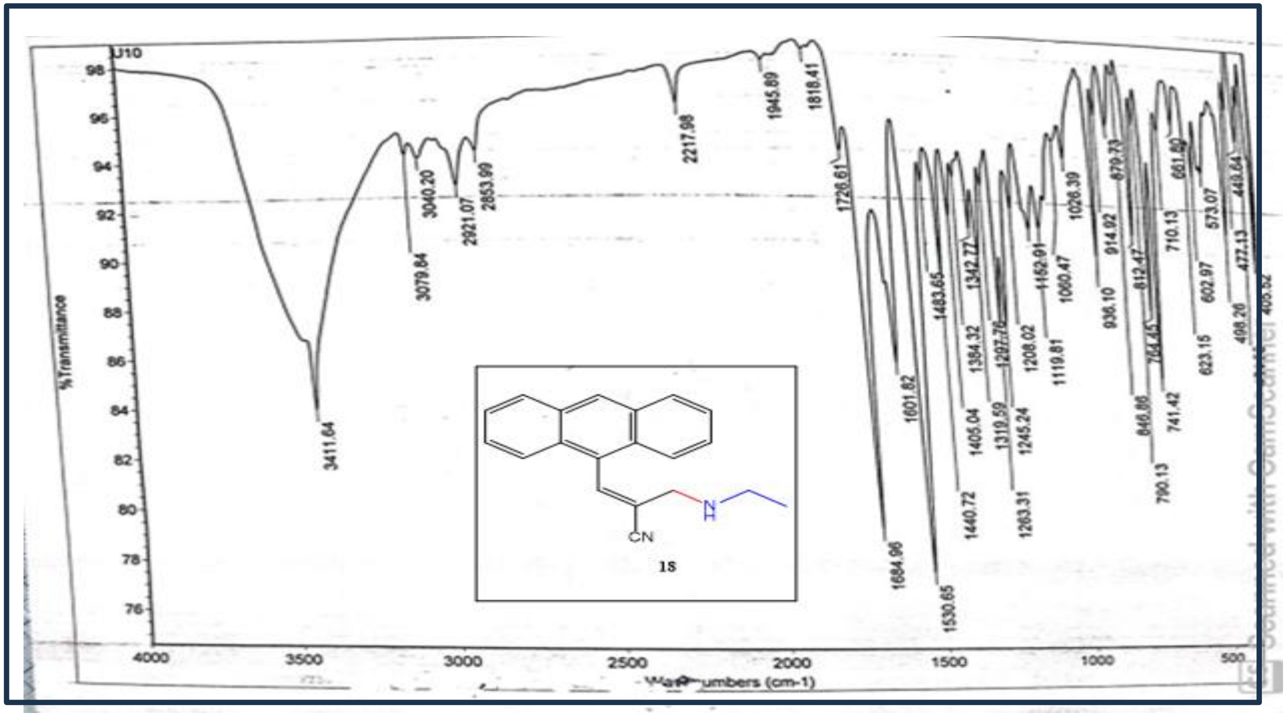

Figure S37. IR spectrum of compound **18**

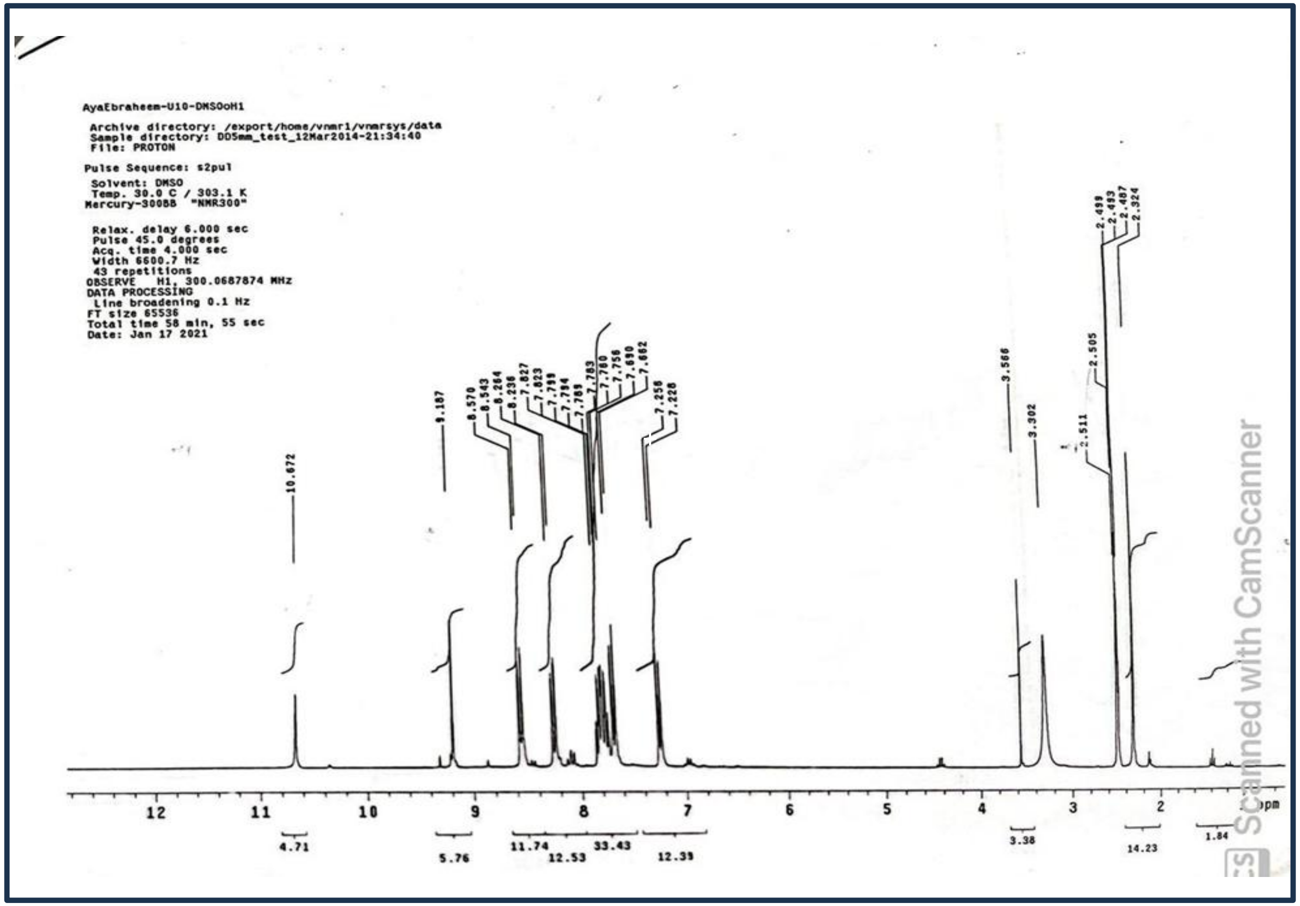

Figure S38. ^{1}H NMR (300 MHz, DMSO-d_6) spectrum of compound **18**

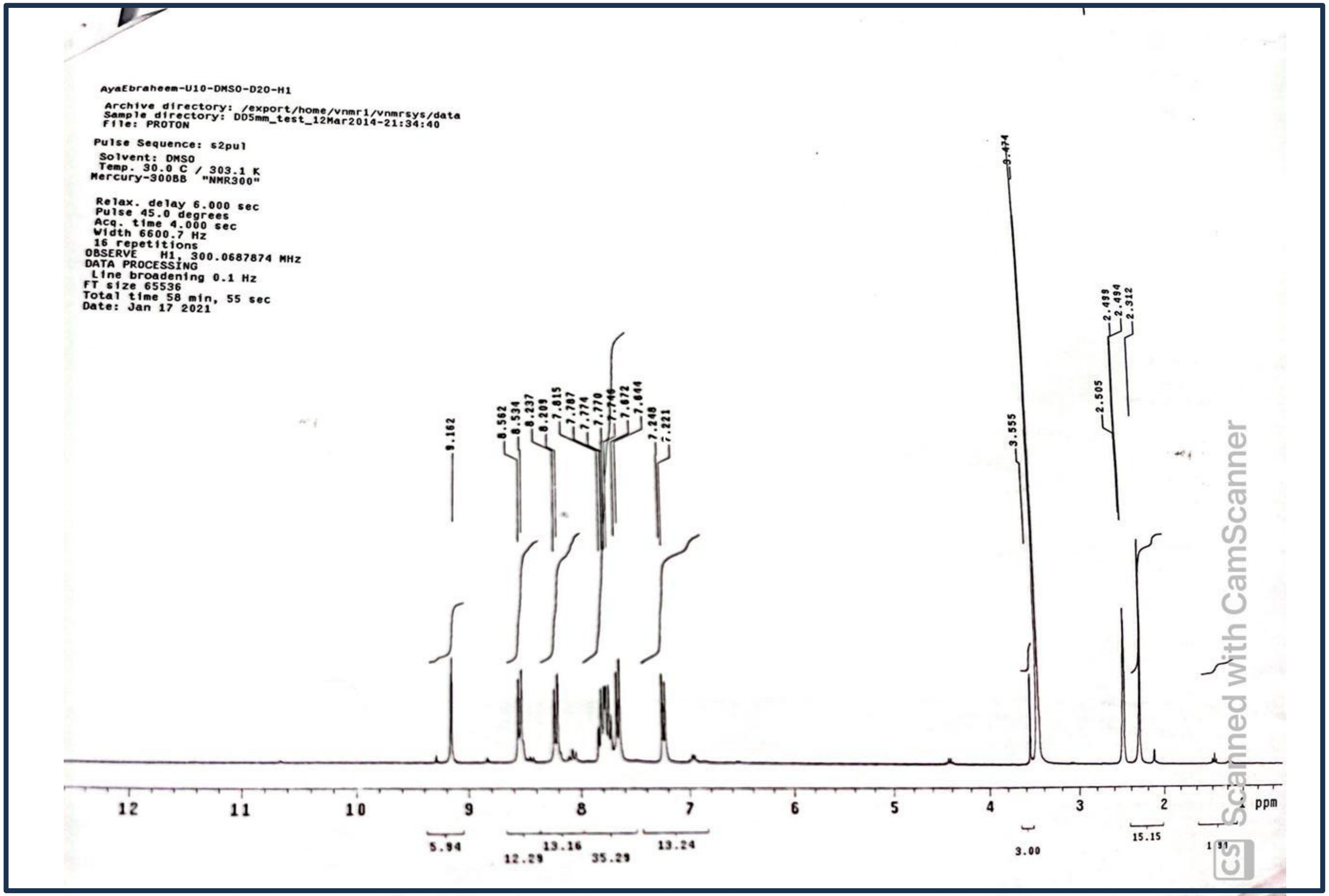
AyaEbraheem-U10-DMSO-D2O-H1
Archive directory: /export/home/vnmr1/vnmrsys/data
Sample directory: DD5mm_test_12Mar2014-21:34:40
File: PROTON
Pulse Sequence: s2pul
Solvent: DMSO
Temp. 30.0 C / 303.1 K
Mercury-300BB "NMR300"
Relax. delay 6.000 sec
Pulse 45.0 degrees
Acq. time 4.000 sec
Width 6600.7 Hz
16 repetitions
OBSERVE H1, 300.0687874 MHz
DATA PROCESSING
Line broadening 0.1 Hz
FT size 65536
Total time 58 min, 55 sec
Date: Jan 17 2021
9.162
8.562
8.534
8.237
8.209
7.815
7.787
7.774
7.770
7.746
7.672
7.644
7.248
7.221
3.555
2.505
2.499
2.494
2.312
12 11 10 9 8 7 6 5 4 3 2 ppm
5.94
12.29
13.16
35.29
13.24
3.00
15.15
Scanned with CamScanner

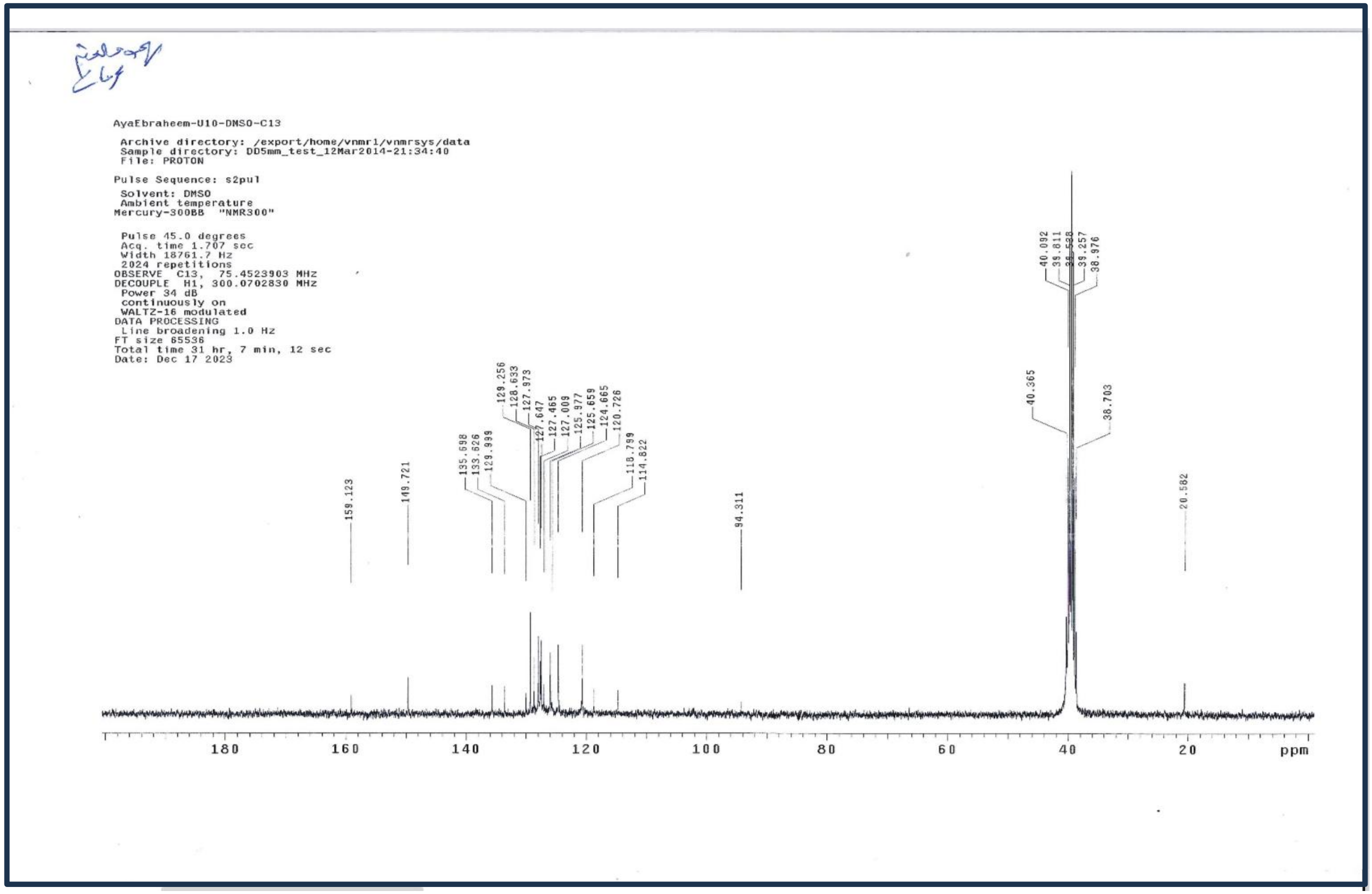

Figure S39. ^{13}C NMR (300 MHz, DMSO-d_6) spectrum of compound **18**

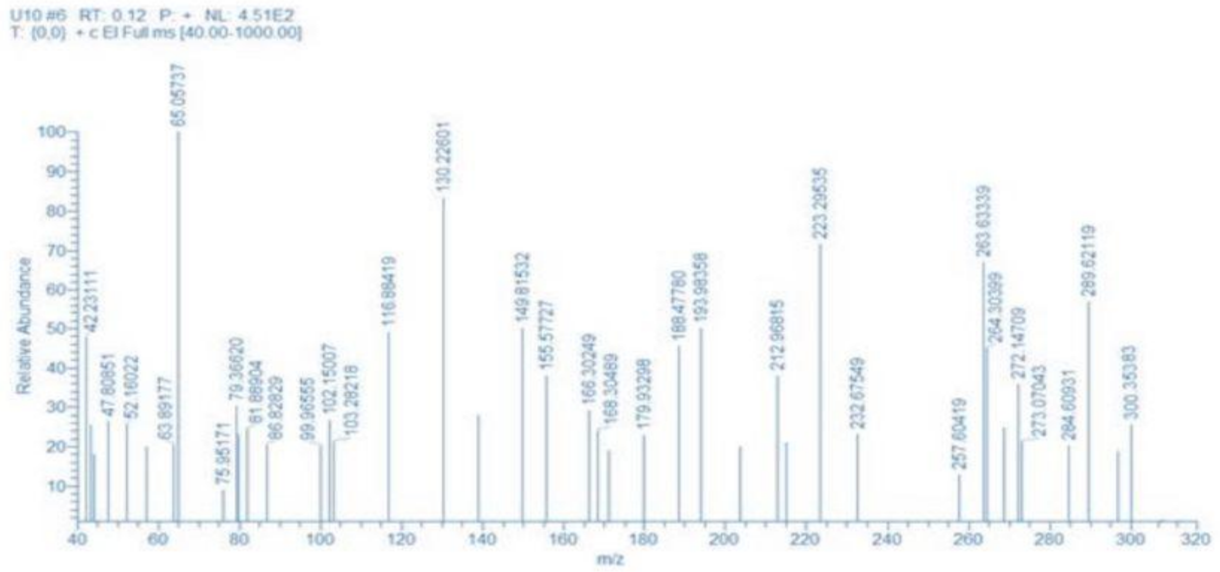

Figure S40. HR-Mass spectrum of compound **18**

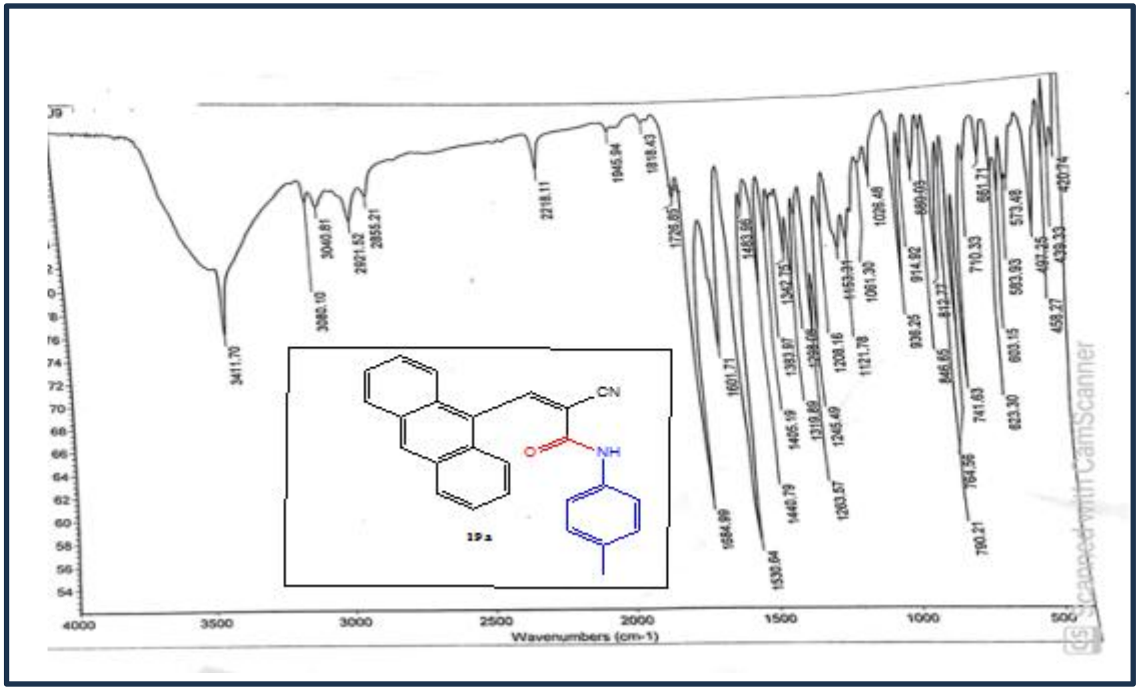

Figure S41. IR spectrum of compound **19a**

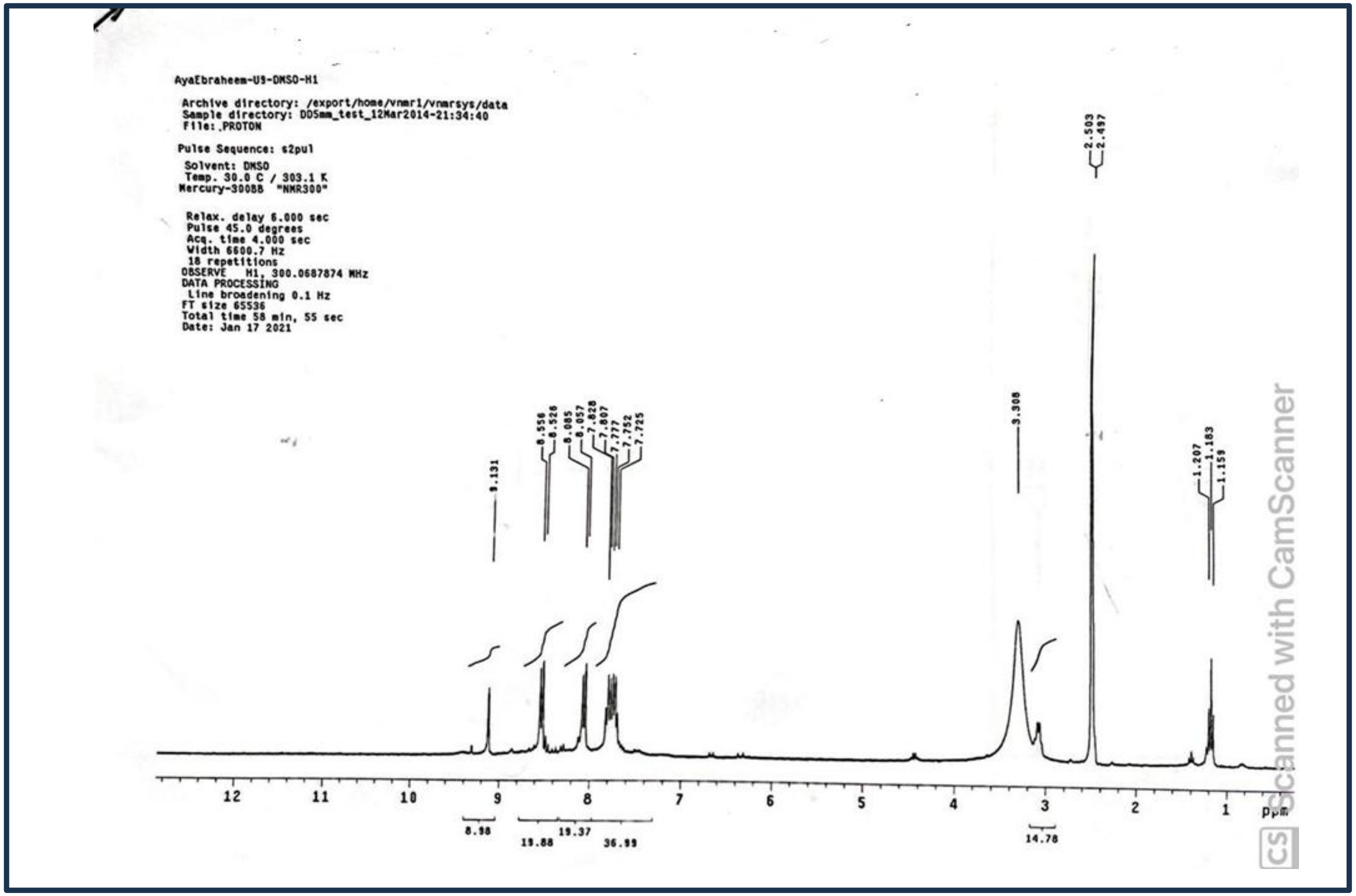

Figure S42. ^{1}H NMR (300 MHz, DMSO-d_6) spectrum of compound **19a**

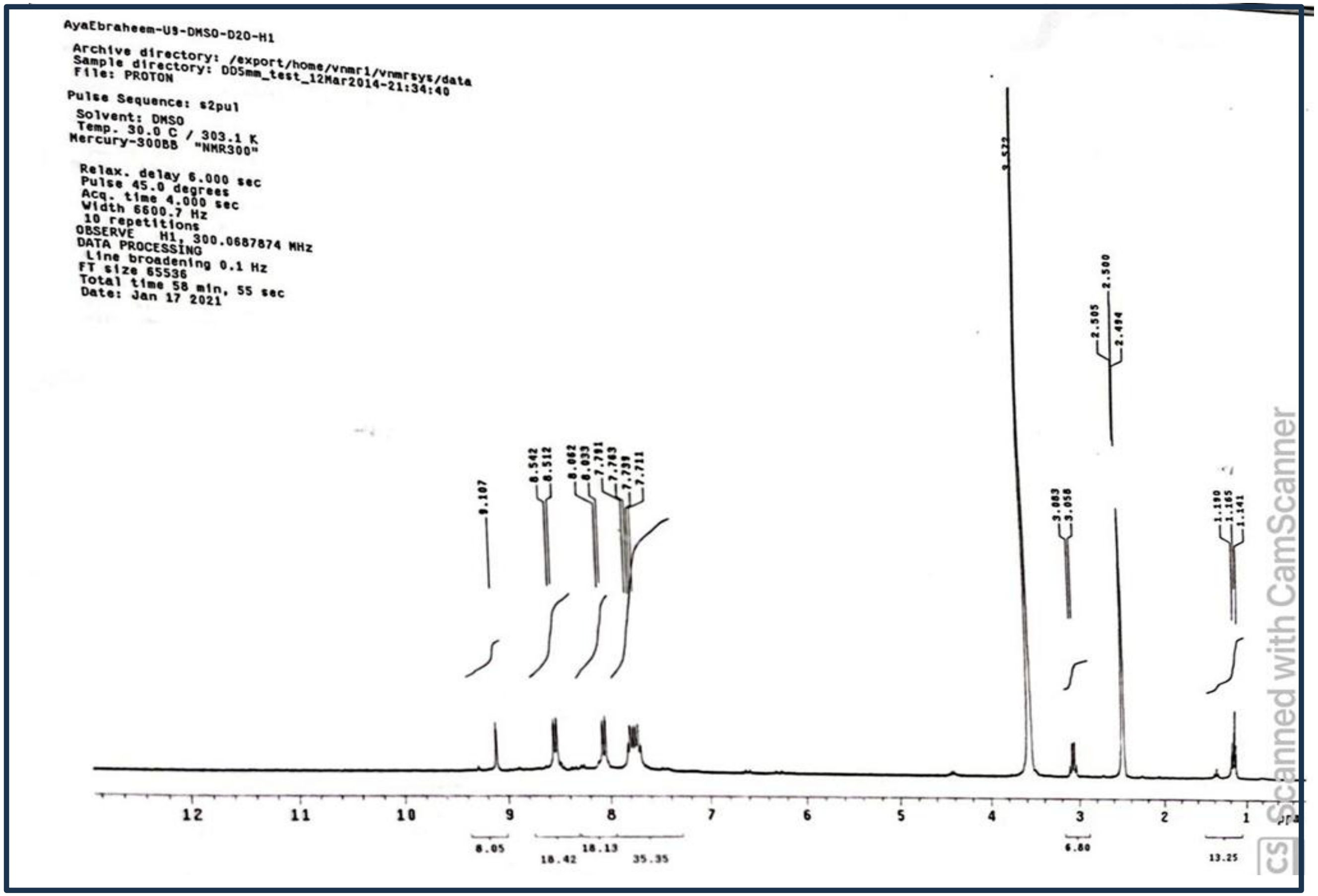
AyaEbraheem-U9-DMSO-D2O-H1
Archive directory: /export/home/vnmr1/vnmrsys/data
Sample directory: DD5mm_test_12Mar2014-21:34:40
File: PROTON
Pulse Sequence: s2pul
Solvent: DMSO
Temp. 30.0 C / 303.1 K
Mercury-300BB "NMR300"
Relax. delay 6.000 sec
Pulse 45.0 degrees
Acq. time 4.000 sec
Width 6600.7 Hz
10 repetitions
OBSERVE H1, 300.0687874 MHz
DATA PROCESSING
Line broadening 0.1 Hz
FT size 65536
Total time 58 min, 55 sec
Date: Jan 17 2021
Scanned with CamScanner

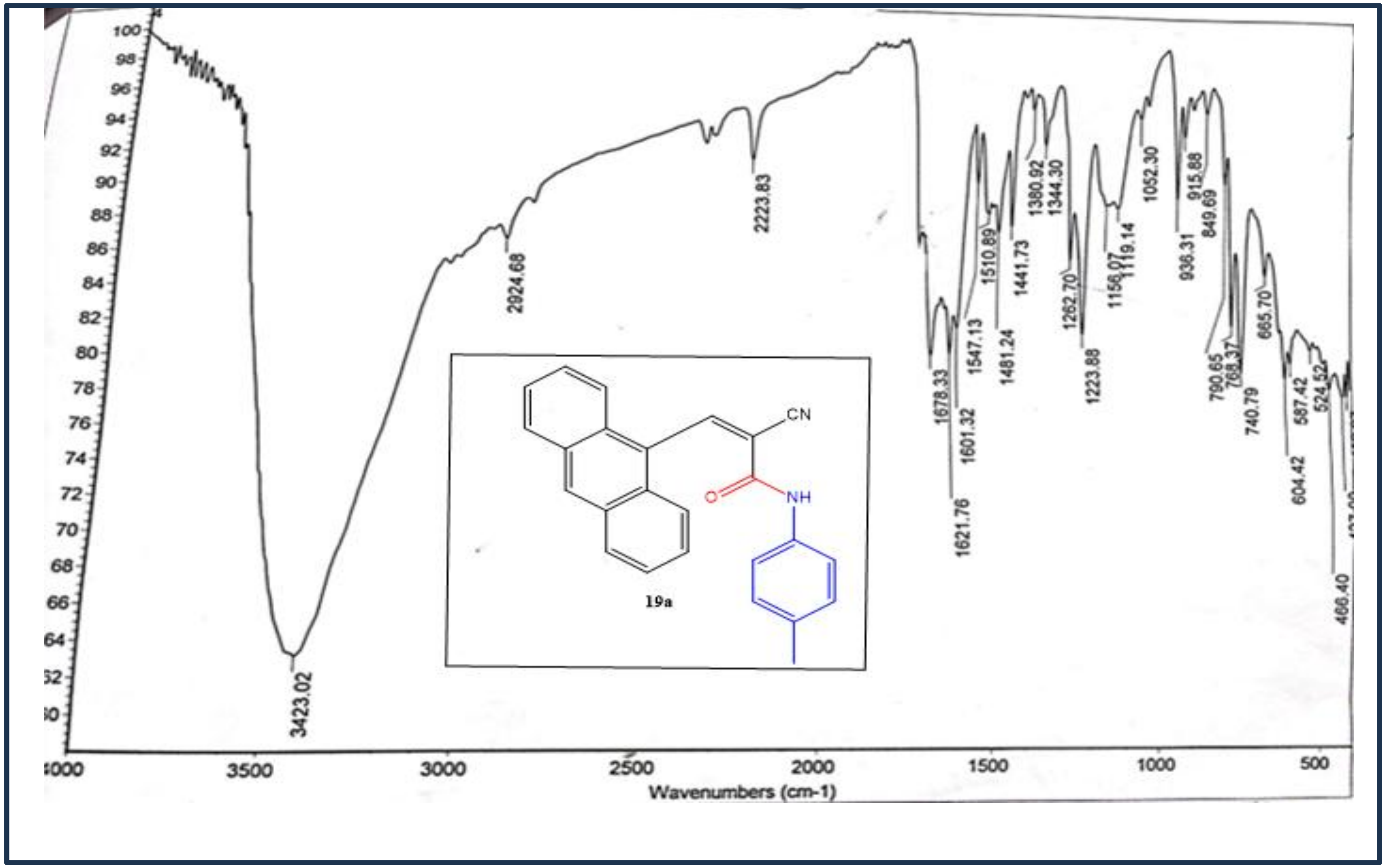

Figure S43. IR spectrum of compound **19b**

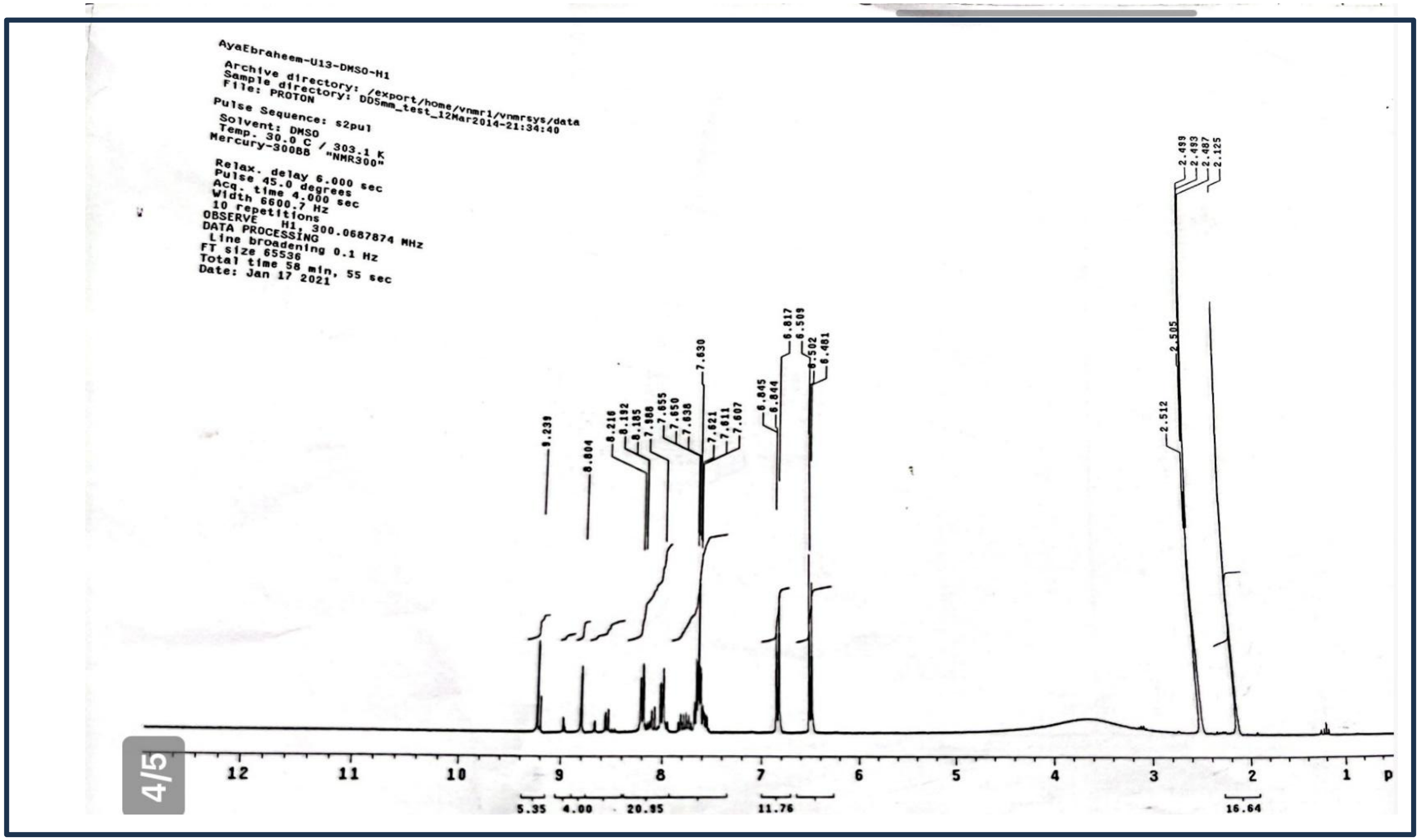

Figure S44. ^{1}H NMR (300 MHz, DMSO-d_6) spectrum of compound **19b**

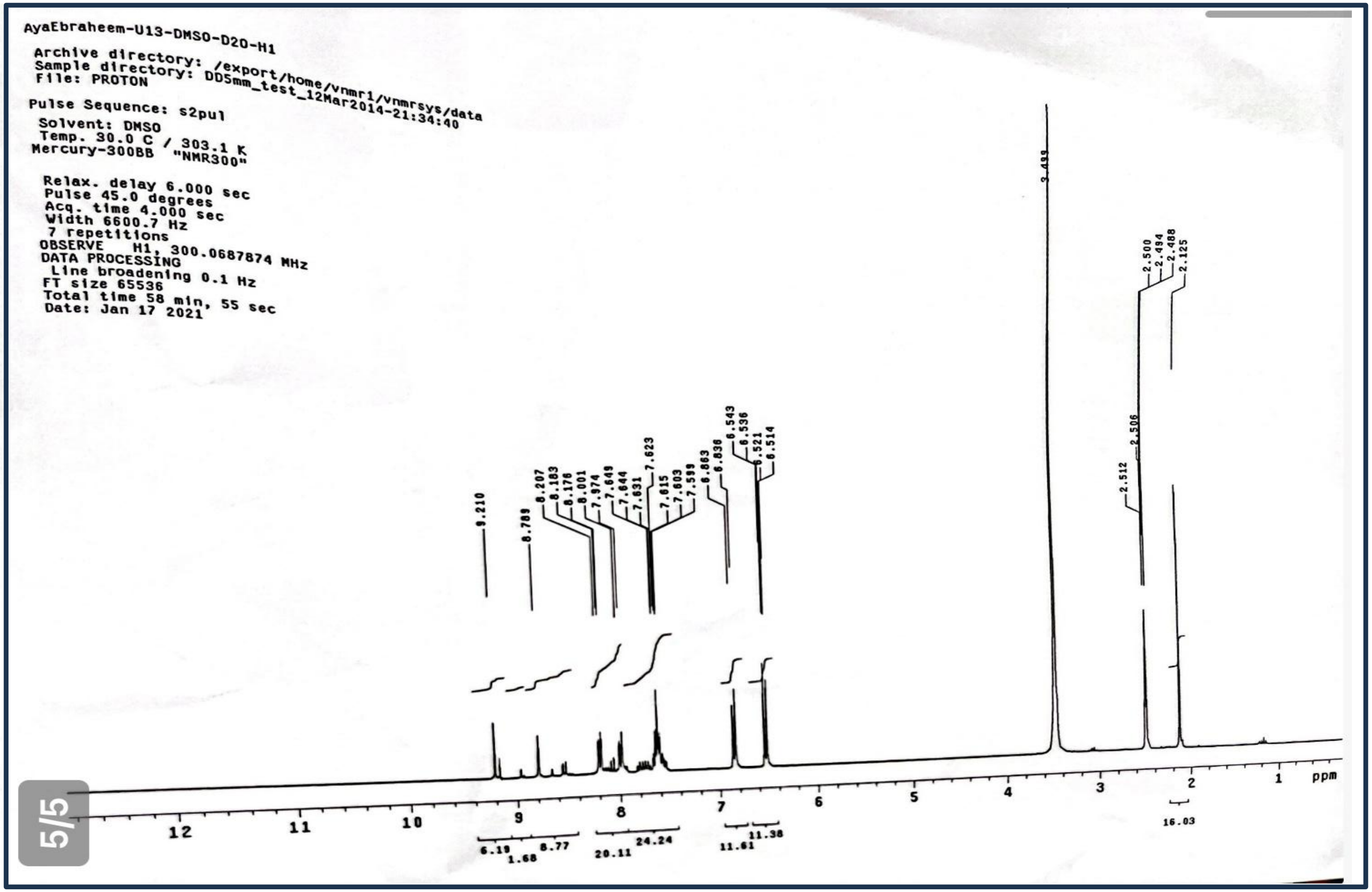
AyaEbraheem-U13-DMSO-D2O-H1
Archive directory: /export/home/vnmr1/vnmrsys/data
Sample directory: DD5mm_test_12Mar2014-21:34:40
File: PROTON
Pulse Sequence: s2pul
Solvent: DMSO
Temp. 30.0 C / 303.1 K
Mercury-300BB "NMR300"
Relax. delay 6.000 sec
Pulse 45.0 degrees
Acq. time 4.000 sec
Width 6600.7 Hz
7 repetitions
OBSERVE H1, 300.0687874 MHz
DATA PROCESSING
Line broadening 0.1 Hz
FT size 65536
Total time 58 min, 55 sec
Date: Jan 17 2021
ppm
5/5
